# A Randomized Trial Assessing the Safety and Immunogenicity of AS01 and AS02 Adjuvanted RTS,S Malaria Vaccine Candidates in Children in Gabon

**DOI:** 10.1371/journal.pone.0007611

**Published:** 2009-10-27

**Authors:** Bertrand Lell, Selidji Agnandji, Isabelle von Glasenapp, Sonja Haertle, Sunny Oyakhiromen, Saadou Issifou, Johan Vekemans, Amanda Leach, Marc Lievens, Marie-Claude Dubois, Marie-Ange Demoitie, Terrell Carter, Tonya Villafana, W. Ripley Ballou, Joe Cohen, Peter G. Kremsner

**Affiliations:** 1 Medical Research Unit, Albert Schweitzer Hospital, Lambaréné, Gabon; 2 GlaxoSmithKline, Biologicals, Rixensart, Belgium; 3 PATH Malaria Vaccine Initiative, Bethesda, Maryland, United States of America; 4 Bill & Melinda Gates Foundation, Seattle, Washington, United States of America; 5 Institute for Tropical Medicine, University of Tübingen, Tübingen, Germany; BMSI-A*STAR, Singapore

## Abstract

**Background:**

The malaria vaccine candidate antigen RTS,S includes parts of the pre-erythrocytic stage circumsporozoite protein fused to the Hepatitis B surface antigen. Two Adjuvant Systems are in development for this vaccine, an oil-in water emulsion – based formulation (AS02) and a formulation based on liposomes (AS01).

**Methods & Principal Findings:**

In this Phase II, double-blind study (NCT00307021), 180 healthy Gabonese children aged 18 months to 4 years were randomized to receive either RTS,S/AS01_E_ or RTS,S/AS02_D_, on a 0–1–2 month vaccination schedule. The children were followed-up daily for six days after each vaccination and monthly for 14 months. Blood samples were collected at 4 time-points. Both vaccines were well tolerated. Safety parameters were distributed similarly between the two groups. Both vaccines elicited a strong specific immune response after Doses 2 and 3 with a ratio of anti-CS GMT titers (AS02_D_/AS01_E_) of 0.88 (95% CI: 0.68–1.15) post-Dose 3. After Doses 2 and 3 of experimental vaccines, anti-CS and anti-HBs antibody GMTs were higher in children who had been previously vaccinated with at least one dose of hepatitis B vaccine compared to those not previously vaccinated.

**Conclusions:**

RTS,S/AS01_E_ proved similarly as well tolerated and immunogenic as RTS,S/AS02_D_, completing an essential step in the age de-escalation process within the RTS,S clinical development plan.

**Trial Registration:**

ClinicalTrials.gov. NCT00307021

## Introduction

The disastrous medical, social and economical burden of malaria in populations of sub-Saharan Africa is well recognized [1], [2]. The development of an effective malaria vaccine would be an important addition to existing malaria control strategies.

The pre-erythrocytic stage *Plasmodium falciparum* antigen RTS,S is the furthest advanced malaria vaccine candidate in clinical development [3]. A collaborative partnership involving several malaria research institutions worldwide, GlaxoSmithKline Biologicals (GSK), the PATH Malaria Vaccine Initiative and the Malaria Clinical Trial Alliance was developed with the goal to develop RTS,S/AS for the Expanded Program on Immunisation (EPI) of the World Health Organization [4].

RTS,S is a hybrid molecule recombinantly expressed in yeast, in which the central tandem repeat and carboxyl-terminal region of the circumsporozoite protein are fused to the N-terminal of the S-antigen of the Hepatitis B virus, creating a particle that also includes the unfused S-antigen. RTS,S is formulated in Adjuvant Systems which enhance the ability of the vaccine to induce a strong immune response. The AS02 Adjuvant System contains an oil-in-water emulsion, monophosphoryl lipid A (MPL), and QS21, a natural saponin molecule purified from the bark of the South American tree, Quillaja saponaria. Following Phase I studies, a Phase IIb efficacy trial conducted in about two thousand Mozambican children aged 1–4 years showed that the vaccine reduced the risk of clinical malaria (vaccine efficacy of 35%) and of severe malaria (vaccine efficacy of 49%) over a period of 18 months [5]. In infants from the same study area receiving the vaccine at 10, 14, and 18 weeks of age, the risk of infection was reduced by 65% over three months after the third and final dose and the risk of clinical disease by 35% over a six-month period following the first dose [6].

In addition to the oil-in-water emulsion of AS02, an alternative Adjuvant System based on liposomes and containing the same amounts of MPL and QS21 has been developed (AS01). An initial study in malaria-naïve adults showed that RTS,S/AS01 had a similar safety profile, a higher humoral immunogenicity, a favorable Th1 cell mediated immune profile and a trend towards higher vaccine efficacy in comparison to RTS,S/AS02 [7].

AS01 and AS02 have been formulated in both adult (AS01_B_, AS02_A_) and pediatric (AS01_E_, AS02_D_) dosages. In the large Phase II trial in Mozambique [8] the pediatric dosage was obtained by administering half of the adult dose (i.e. 0.25 mL of AS02_A_). In order to comply with EPI standards, the vaccine volume was changed to 0.5 mL. The equivalency of 0.25 mL AS02_A_ and 0.5 mL AS02_D_, both containing 25 µg RTS,S, in terms of safety and immunogenicity was shown in children in Mozambique [9].

As part of an age de-escalation step in the RTS,S clinical vaccine development plan, we conducted a phase II, randomized, double-blind study to assess the safety and immunogenicity of RTS,S/AS01_E_ and RTS,S/AS02_D_ in children aged 18 months to 4 years living in Gabon. The resulting data will make a significant contribution in deciding which Adjuvant System to use for a large scale large efficacy trial with RTS,S/AS. Furthermore, we hoped to obtain information on the immunogenic effect of the third dose, compared to two doses, and the effect of previous hepatitis B immunisation on the anti-CS immune response.

## Materials and Methods

The protocol for this trial [http://clinicaltrials.gov/: NCT00307021] and supporting CONSORT checklist are available as supporting information; see Checklist S1 and Protocol S1.

### Ethics statement

The study protocol was approved by the ethics committee of the International Foundation of the Albert Schweitzer Hospital of Lambaréné and the Western Institutional Review Board, USA. The trial was undertaken according to the International Conference on Harmonisation of Good Clinical Practice guidelines and was monitored by GSK Biologicals, Rixensart, Belgium. A local safety monitor and a data and safety monitoring board closely reviewed the conduct and results of the trial. After vaccination of Dose 1 and Dose 2 of the first 30 subjects, the data and safety monitoring board reviewed the seven day post vaccination safety data in order to decide on the continuation to the next dose.

### Study site

The study lasted from April 2006 to August 2007 and was conducted at the Medical Research Unit (MRU) of Albert Schweitzer Hospital in Lambaréné, located in the central part of Gabon. Malaria transmission is mainly attributable to *Plasmodium falciparum*. *Anopheles gambiae* is the main vector and transmission is perennial, moderate to high in intensity [10], with an average entomological inoculation rate (EIR) around 50 infective bites per person per year [11].The incidence of malaria in children was around 1.5 per child per year in 1999 [12], but has decreased in recent years [13]. Long-lasting insecticide-treated bednets are provided free of charge for pregnant women and children under 12 months of age during national health campaigns. A study carried out in 2005 revealed a high proportion of the population sleeping under bednets, however these often had never been impregnated (94%) and had holes (20%) [14].

Coverage of routine EPI vaccination in the study area is above national average [15] with a recent survey showing 87% of children having received three doses of DTP at 4 months of age and 74% had received measles vaccination at 9 months of age (Bertrand Lell, unpublished data).

### Study design

This was a Phase II randomized, double-blind trial to describe safety, reactogenicity and immunogenicity of RTS,S/AS01_E_ and RTS,S/AS02_D_ when administered intramuscularly with a 0, 1, 2 month vaccination schedule in children aged 18 months to 4 years. The primary objectives were to assess the occurrence of serious adverse events from the time of first vaccination until one month post Dose 3 and to establish the non-inferiority of anti circumsporozoite antibodies titers of RTS,S/AS01_E_ compared to RTS,S/AS02_D_ at one month post Dose 3. A SAE was defined per protocol as any untoward medical occurrence that was fatal, life-threatening, required hospitalization, led to disability or incapacity, or was judged by investigators as being medically important enough to be reported as serious. In order to maximize data capture about seizures, all seizures occurring within 30 days of vaccination had to be reported as SAEs. Data on seizures occurring within 7 days post vaccination were collected in a standard way according to Brighton collaboration guidelines [16].

Secondary objectives included reactogenicity, anti-HBs antibody response and anti-CS response, and the 1 year post last dose safety and immunogenicity follow up.

### Study vaccines

RTS,S/AS02_D_ (0.5 mL) and RTS,S/AS01_E_ (0.5 mL) comprise the RTS,S antigen presented as a lyophilized pellet, which is reconstituted prior to injection with 0.5 mL of AS02_D_ or AS01_E_ liquid Adjuvant Systems containing MPL and QS21 immunostimulants. The RTS,S pellet and Adjuvant Systems are in sterile glass vials, stored between +2 and +8°C.

### Study population

We included children aged 18 months to 4 years (up to but not including 5th birthday) who were permanent residents in Lambaréné. Written informed consent or, in case of illiteracy, a thumb print in presence of a literate witness was obtained for all screened children from both parents/guardians, or at least one parent in case the other could not be reached. Major exclusion criteria were history of allergic disease, a weight for age Z-score less than −2, and clinically significant chronic or acute disease.

### Randomization and blinding

Eligible children were allocated to a treatment group on the day of first vaccination. The randomization list, designed as a single block randomization, was generated at GSK Biologicals, Rixensart, using SAS® statistical software. Safety and immunogenicity endpoints were evaluated in a double-blind manner as the investigators and their parent(s)/guardian(s) were unaware which vaccine was administered to a particular child. Only two study nurses and an observer responsible for vaccine storage, preparation and quality control were aware of the vaccine assignment. They were not involved in other aspects of the trial or patient care. Code break envelopes with individual vaccine allocation were kept locally with a local safety monitor. A second copy was kept at GSK Biologicals, Rixensart, Belgium. There was no case of emergency unblinding during the study. Vaccine preparation, vaccination and clinical observation were performed in separated rooms. Vaccine preparation consisted of reconstituting the lyophilized antigen using the liquid Adjuvant System fraction, stored in separate vials, to a final volume of 0.5 mL.

### Study procedures

Before screening, the parents or guardian was issued a bednet containing an impregnation kit and instructions on its use. Screening procedures included a brief medical history, examination and blood sampling for hematology (hemoglobin, white cell count, and platelets), and biochemistry (ALT, creatinine, and bilirubin). The past Hepatitis B immunization status as documented on the children's national immunization cards was recorded. Vaccine administration was by slow IM injection in the left deltoid. After vaccination, children were observed for at least one hour by investigators trained in pediatric emergency care and thereafter visited by field workers daily for the next six days. General symptoms and those specific to RTS,S vaccination in particular were assessed. These ‘solicited symptoms’ (local symptoms: pain, swelling; generalized symptoms: drowsiness, fever, irritability, loss of appetite) were graded and recorded on diary cards. Grade 3 symptoms were defined, for pain, when a child cries when limb is moved or spontaneously painful; for swelling when larger than 20 mm; and for general symptoms, as those that prevent normal activities. Unsolicited adverse events were documented over a period of 30 days post vaccination and serious adverse events were recorded up to one year post last vaccine dose. Local standard medical care was provided free of charge throughout the study.

### Laboratory methods

As part of safety assessments, venous blood samples were collected for hematology (hemoglobin, white cell count, platelets; ABX Pentra 60 analyzer, France) and biochemistry (ALT, creatinine and bilirubin; Cobra Mira Plus analyzer, Switzerland) determination on Day 6 after Dose 1 and one month post Dose 3.

Blood samples for the measurement of anti-circumsporozoite protein (CS) and antibody to the hepatitis B surface antigen (anti-HBsAg) were collected at screening and one month post Dose 2 and 3, and at study month 14. Serological responses to CS repeats (anti R32LR) were assessed by standard ELISA methodology using a plate adsorbed R32LR antigen with a standard reference antibody as a control with concentrations reported as EU/mL. Antibody titers to hepatitis B surface antigen were measured using an ELISA immunoassay, developed at GSK Biologicals, Rixensart with concentrations reported in mIU/mL. All ELISA assays were performed in a GLP ICH validated laboratory.

### Populations analyzed

The ITT cohort for analysis of safety included all subjects who received at least one vaccine dose. The ATP cohort for analysis of immunogenicity included all evaluable subjects (i.e., those meeting all eligibility criteria, complying with the procedures defined in the protocol, having no elimination criteria during the study) for whom data concerning immunogenicity endpoint measures were available.

### Statistical analysis

A sample size of 75 evaluable subjects per group was determined to have 90% power to demonstrate non-inferiority of RTS,S/AS01_E_ versus RTS,S/AS02_D_ in terms of anti-CS immune response (upper limit of 95% CI of the GMT ratio RTS,S/AS02_D_ versus RTS,S/AS01_E_ below 3.0), assuming a log standard deviation of 0.9 in both groups, and an alpha level of 2.5%. To allow for loss to follow-up, 90 children were included in each group. The Geometric Mean Titers (GMTs) calculations were performed by taking the anti-log of the mean of the log10 titer transformations. The 95% CI of the ratio of anti-CS GMTs was calculated using a one-way ANOVA model on the logarithm transformation of the titers. Antibody titers below the cut-off of the assay were given an arbitrary value of half the cut-off for the purpose of GMT calculation. Subjects with anti-HBs antibody titers greater than 10 mIU/mL were considered protected.

## Results

**Figure 1:**
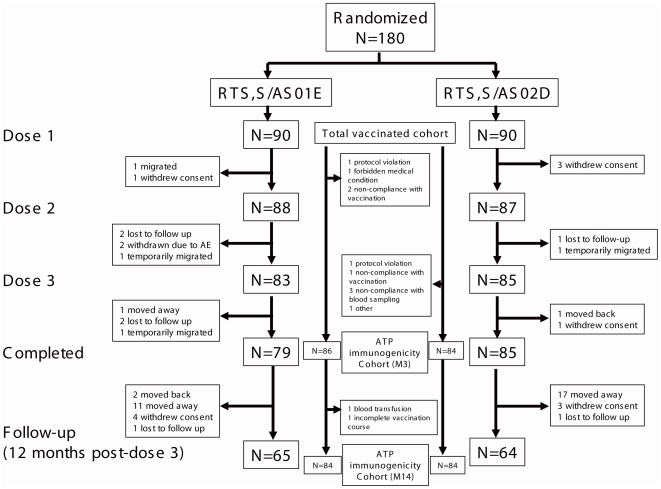
Subject disposition.

Out of 280 children screened for eligibility, 180 were randomized into two intervention groups. The demographic characteristics were similar in the two groups, with a mean age of 39.6 (SD: 11.5) months and 39.3 (SD: 11.2) months and with 36 (40%) and 52 (58%) females in the RTS,S/AS01_E_ and the RTS,S/AS02_D_ group, respectively. Figure 1 shows the disposition of subjects in the study.

### Safety

The vaccine safety was analyzed based on the 180 children of the ITT population. Between first vaccination and 1 month post Dose 3, three children in the RTS,S/AS01_E_ group and four in the RTS,S/AS02_D_ group experienced at least one serious adverse event (Table 1),. Two children were excluded from further vaccination due to a serious adverse event, diagnosed with conditions that would have precluded study participation if found before enrolment: one case of newly diagnosed sickle cell, and one case of a simple febrile convulsion that occurred 18 days postvaccination, in the context of an uncharacterized acute febrile illness that evolved favorably over 3 days. The other serious adverse events included anemia, asthma, gastroenteritis, pneumonia, and incarcerated umbilical hernia. Between first vaccination and study Month 14, 16 subjects experienced at least one serious adverse events (8 in each group) (Table 1). None of these events were considered related to the study vaccine and all subjects recovered.

**Table 1:** Listing of SAEs that occurred during the whole study period.

Group	Subject No.	Gender	Age at onset (Month)	Event (Preferred term)	Onset (days post Dose)	Vaccine relatedness
AS01_E_	9	M	33	Febrile convulsion	18d post D2	No
				Pyrexia	17d post D2	No
	66	M	58	Hydrocele	272d post D3	No
				Phimosis	272d post D3	No
	104	M	37	Constipation	97d post D3	No
	115	M	57	Bronchitis	286d post D3	No
	125	F	28	Pneumonia	216d post D3	No
	181	M	30	Anaemia	7d post D3	No
				Asthma	7d post D3	No
				Pneumonia	6d post D3	No
	234	F	42	Anaemia	101d post D3	No
				Malaria	96d post D3	No
	277	F	51	Hepatitis A	2d post D2	No
				Sickle cell anaemia	5d post D2	No
AS02_D_	1	M	33	Umbilical hernia, obstructive	21d post D2	No
	72	F	34	Asthma	81d post D3	No
				Lower respiratory tract infection	77d post D3	No
				Pharyngitis	77d post D3	No
	88	F	25	Gastroenteritis	26d post D3	No
			28	Burns second degree	122d post D3	No
	117	F	40	Anaemia	47d post D3	No
				Cerebral malaria	42d post D3	No
			42	Plasmodium falciparum infection	92d post D3	No
	138	F	27	Gastroenteritis	30d post D3	No
	141	M	22	Gastroenteritis	9d post D3	No
	161	M	30	Asthma	126d post D3	No
			33	Pneumonia	230d post D3	No
	198	M	38	Epilepsy	84d post D3	No
			44	Epilepsy	238d post D3	No
			45	Epilepsy	273d post D3	No

Solicited adverse events are summarized in Table 2. Mild local solicited symptoms were frequent with pain being reported following 48% and 54% of doses of RTS/AS01_E_ and RTS,S/AS02_D_ respectively and swelling being present following 32% and 37% of doses of RTS/AS01E and RTS,S/AS02_D_. Grade 3 symptoms were rare, being present only after two vaccine doses administered. These were two cases of swelling over 20 mm, one in each study group. There was an increase in the frequency of local symptoms for subsequent doses for both groups. General solicited symptoms were considerably less frequent, with loss of appetite observed most frequently, after 44 (17%) doses of RTS,S/AS01_E_ and 46 (18%) of RTS,S/AS02_D_ doses. Frequencies of general solicited symptoms were similar between the intervention groups and no increase in frequency for progressive doses was found. No grade 3 solicited generalized symptoms were observed following vaccination. Previous Hepatitis B vaccination did not significantly affect the incidence of solicited symptoms following vaccination with either study vaccine (data not shown).

**Table 2:** Solicited symptoms per dose during a 7-days post-vaccination period.

	RTS,S/AS01_E_		RTS,S/AS02_D_
	N	n (%; 95%CI)	N	n (%; 95%CI)
**Generalized symptoms:**				
Drowsiness, overall	261	17 (7%; 4–10%)	262	13 (5%; 3–8%)
Fever, overall	261	15 (6%; 3–9%)	262	23 (9%; 6–13%)
Irritability, overall	261	6 (2%; 1–5%)	262	8 (3%; 1–6%)
Loss of appetite, overall	261	44 (17%; 13–22%)	262	46 (18%; 13–23%)
**Local symptoms:**				
Pain, overall	261	126 (48%; 42–55%)	262	142 (54%; 48–60%)
Dose 1	90	19 (21%; 13–31%)	90	32 (36%; 26–46%)
Dose 2	88	34 (39%; 28–50%)	87	34 (39%; 29–50%)
Dose 3	83	73 (88%; 79–94%)	85	76 (89%; 81–95%)
Swelling, overall	261	83 (32%; 26–38%)	262	96 (37%; 31–43%)
Dose 1	90	5 (6%; 2–13%)	90	13 (14%; 8–23%)
Dose 2	88	21 (24%; 15–34%)	87	22 (25%; 17–36%)
Dose 3	83	57 (69%; 58–78%)	85	61 (72%; 61–81%)

N: number of vaccine doses administered.

Overall, unsolicited adverse events in the 30 days post vaccination period occurred in 78.9% of the participants of the RTS,S/AS01_E_ group and in 82.2% of the RTS,S/AS02_D_ group (Table 3). The most frequently observed unsolicited symptoms were upper respiratory tract infection and other common pediatric conditions. None were considered to be causally related to the study vaccines.

**Table 3:** Occurrence of unsolicited symptoms within the 30-day (Days 0–29) post-vaccination period.

	RTS,S/AS01_E_				RTS,S/AS02_D_			
	N = 90		95% CI		N = 90		95% CI	
Preferred Term	n	%	LL	UL	n	%	LL	UL
At least one event	71	78.9	69.0	86.8	74	82.2	72.7	89.5
Diarrhoea	5	5.6	1.8	12.5	9	10.0	4.7	18.1
Pyrexia	5	5.6	1.8	12.5	5	5.6	1.8	12.5
Acarodermatitis	5	5.6	1.8	12.5	6	6.7	2.5	13.9
Bronchitis	9	10.0	4.7	18.1	11	12.2	6.3	20.8
Gastroenteritis	4	4.4	1.2	11.0	9	10.0	4.7	18.1
Helminthic infection	17	18.9	11.4	28.5	11	12.2	6.3	20.8
Nasopharyngitis	41	45.6	35.0	56.4	41	45.6	35.0	56.4
Otitis media	5	5.6	1.8	12.5	2	2.2	0.3	7.8
Staphylococcal skin infection	10	11.1	5.5	19.5	13	14.4	7.9	23.4
Tinea capitis	6	6.7	2.5	13.9	5	5.6	1.8	12.5
Upper respiratory tract infection	5	5.6	1.8	12.5	4	4.4	1.2	11.0

Percentage of subjects reporting the occurrence of unsolicited symptoms classified by MEDDRA Preferred Term within the 30-day (Days 0–29) post-vaccination period, for events that occurred in over 5% of the patient in at least one of the study groups.

N = number of subjects with at least one administered vaccine dose.

n/% = number/percentage of subjects reporting at least once the symptom.

95% CI = exact 95% confidence interval; LL = Lower Limit, UL = Upper Limit.

Out of range hematological and biochemical values were observed in 21 measurements. No Grade 3 abnormalities were observed. The abnormal values were all judged not to be clinically significant and there was no imbalance between vaccine groups.

### Immunogenicity

The antibody response was evaluated in the ATP population, consisting of 170 children, balanced by group. The primary immunogenicity endpoint of the trial was met: at 30 days post Dose 3, the ratio of GMT titers (AS02_D_/AS01_E_) was 0.88 (95% CI: 0.68–1.15), with an upper confidence interval below the pre-defined non-inferiority cut-off of three. Both vaccines elicited a strong specific immune response after Dose 2 and Dose 3 (Table 4). For both vaccines, the antibody titers were significantly higher after the third dose compared to the first two doses. There was a trend towards higher anti-CS GMTs in the recipients of RTS,S/AS01_E_ compared to the RTS,S/AS02_D_ group at post Dose 2 and post Dose 3, but these were of similar magnitude at study Month 14.

**Table 4:** Anti-CS and anti-HBs antibody titers after each dose and at 12 months post Dose 3.

	RTS,S/AS01_E_			RTS,S/AS02_D_		
	N	mean	95%CI	N	mean	95%CI
**Anti-CS antibodies:**						
**Screening, all**	85	**0.3**	**0.3–0.3**	84	**0.3**	**0.3–0.3**
- pre HBV	40	0.3	0.3–0.3	40	0.3	0.3–0.3
- no pre HBV	37	0.3	0.3–0.3	39	0.3	0.2–0.3
**Post Dose 2, all**	74	**80**	**63–101**	74	**58**	**46–73**
- pre HBV	35	109	79–152	34	81	57–114
- no pre HBV	31	50	35–71	36	44	33–59
**Post Dose 3, all**	73	**207**	**172–250**	73	**183**	**151–223**
- pre HBV	35	238	187–302	36	201	145–278
- no pre HBV	31	170	122–238	33	164	128–209
**Month 14, all**	58	**16**	**12–21**	54	**18**	**13–26**
- pre HBV	29	15	10–23	29	18	10–32
- no pre HBV	23	17	10–28	21	17	10–29
**Anti-HBs antibodies:**						
**Screening, all**	85	**45**	**25–80**	84	**19**	**12–29**
- pre HBV	40	284	127–637	40	61	30–124
- no pre HBV	37	8	5–11	39	6	4–9
**Post Dose 2, all**	74	**9228**	**4676–18210**	74	**3838**	**2036–7237**
- pre HBV	35	79610	43152–146869	34	22946	10115–52053
- no pre HBV	31	1095	562–2135	36	770	385–1540
**Post Dose 3, all**	73	**26330**	**16578–41821**	73	**20068**	**13636–29533**
- pre HBV	35	94879	56708–158741	36	48279	28412–82038
- no pre HBV	31	7982	5534–11511	33	8292	5233–13138
**Month 14, all**	58	**6769**	**4338–10561**	54	**5236**	**3421–8012**
- pre HBV	29	17851	10235–31136	29	8408	4467–15825
- no pre HBV	23	2539	1723–3746	21	3100	1708–5625

Values are geometric mean titers and 95% confidence intervals expressed in EU/mL. N values for pre HBV and no pre HBV do not match up to the total due to some missing values on the pre HBV status.

**Figure 2:**
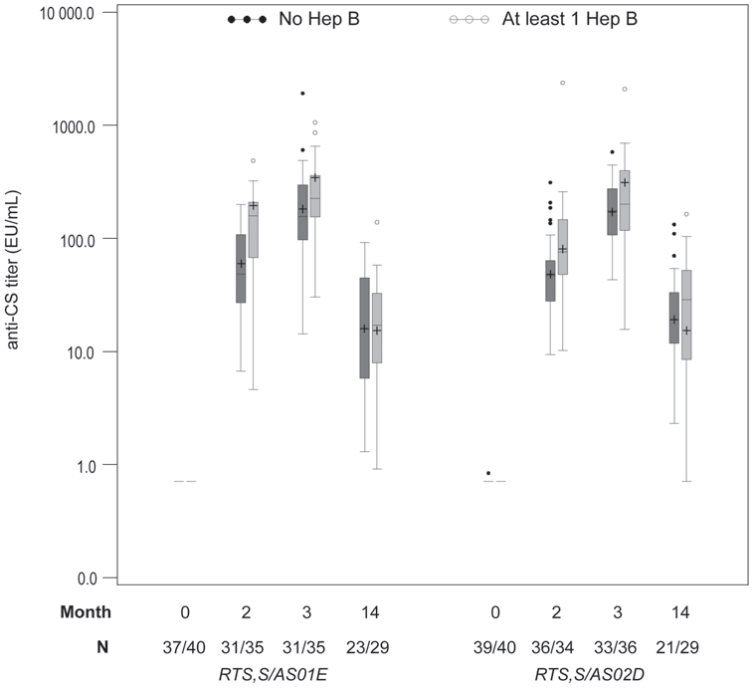
The effect of previous hepatitis B vaccination on anti-CS antibodies. Boxplot graph of anti-CS responses. Represents Q25, median, Q75, highest and lowest observation below/above 1.5 times interquartile range and individual data points below/above 1.5 times interquartile range. GMTs are indicated by +.

In both study groups, an equivalent proportion of children had previously received hepatitis B vaccination. After Dose 2 and Dose 3 of experimental vaccine, anti-CS antibody GMTs were higher in the children who had been previously vaccinated with at least one dose of hepatitis B vaccine compared to those who had not been previously vaccinated (Table 4, Figure 2). No difference in GMTs was apparent at study Month 14.

**Figure 3:**
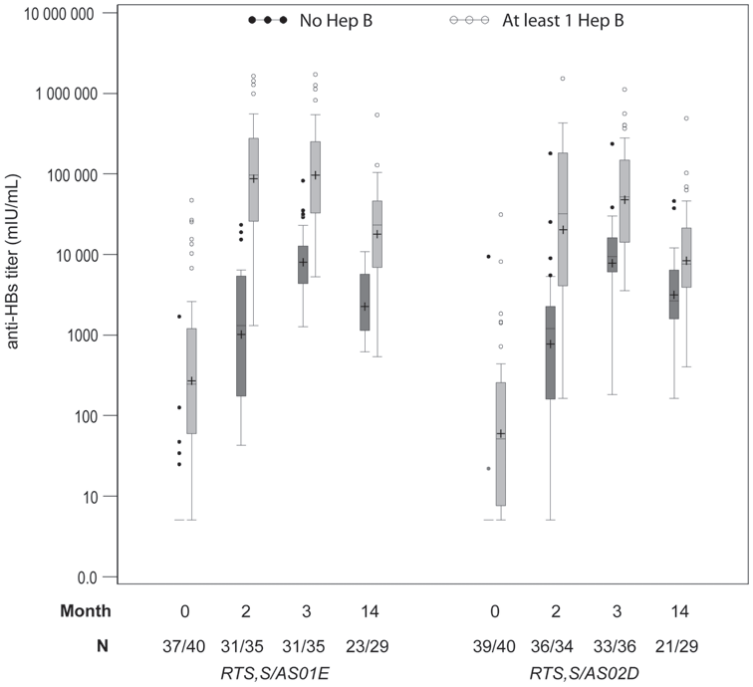
The effect of previous hepatitis B vaccination on anti-HBs antibodies. Boxplot graph of anti-HBs responses. Represents Q25, median, Q75, highest and lowest observation below/above 1.5 times interquartile range and individual data points below/above 1.5 times interquartile range. GMTs are indicated by +.

Both RTS,S/AS01_E_ and RTS,S/AS02_D_ were highly immunogenic for anti-HBs antibodies. Over 98% of the children had anti-HBs titers above the seroprotection level following Dose 2 and all children were protected following Dose 3, with no difference between groups. There was a trend towards higher anti-HBs GMTs in the recipients of RTS,S/AS01_E_ compared to the RTS,S/AS02_D_ group, following both Dose 2 and Dose 3, with a AS02_D_/AS01_E_ GMT ratio of 0.76 (95% CI: 0.42–1.39) after the third dose (Table 4, Figure 3).

Children who had previously received a hepatitis B vaccine had higher anti-HBs GMTs at screening than those who were not vaccinated and the difference remained statistically significant at all three timepoints for either vaccine. Post Dose 3, GMTs for recipients of RTS,S/AS01_E_ were 94879 mIU/mL and 7982 mIU/mL and for recipients of RTS,S/AS02_D_ were 48279 mIU/mL and 8292 mIU/mL with previous hepatitis B vaccination and without previous hepatitis B vaccination, respectively (Table 4).

## Discussion

A series of clinical studies performed with more than a thousand vaccine recipients have established the RTS,S/AS02 candidate vaccine to have an promising safety profile and to be well-tolerated. These studies were conducted in malaria-naïve and semi-immune adults as well as African children, with the first demonstration of efficacy for a malaria candidate vaccine [5], [8], [17]–[20]. Pre-clinical data suggests that RTS,S/AS01 may be a better malaria vaccine candidate [21]. A challenge study conducted in the United States showed that RTS,S/AS01, as compared to RTS,S/AS02, induced higher levels of anti-CS antibodies and CS-specific T-cells, and a trend towards higher protection against infection following experimental sporozoite challenge. The safety profile of both vaccines was similarly favorable [7]. The safety and high immunogenicity of the RTS,S/AS01 formulation was confirmed in malaria-exposed adults in Kenya [22]. Here, the first use of RTS,S associated with the AS01 Adjuvant System in children is reported.

In the present study, both vaccines were well tolerated. In both groups, patterns of non-serious and serious adverse events were in accordance with general morbidity observed in this population. There were no unsolicited adverse events related to vaccination. Vaccination induced no hematological or biochemical abnormalities. The vast majority of solicited symptoms consisted of mild pain at the injection site, confirming results of previous RTS,S/AS01 and RTS,S/AS02 studies. The frequency of local solicited symptoms (pain and swelling) was dose related and progressively increased from Dose 1 to 3 in both vaccines groups. This trend had been reported in previous trials with RTS,S/AS02 [19], [20], as well as with other vaccines [23], showing that this is not specific to the malaria candidate vaccine or specific Adjuvant System technology, and is probably linked to a local reaction between antigen and immune effectors like antibodies and primed T-cells. Importantly, these reactions remained moderate in intensity even after the third dose. In summary, all safety data generated in this trial show that both formulations of the RTS,S/AS candidate vaccine have a favorable safety profile. Larger studies are needed to allow detection of potential rare events.

Our study clearly confirms our primary hypothesis, i.e., the non-inferiority of RTS,S/AS01_E_ compared to RTS,S/AS02_D_ in its ability to induce anti-CS antibodies. In fact, an early trend towards higher anti-CS GMTs in recipients of RTS,S/AS01_E_ was found. The trend toward a stronger immune response using the AS01_E_ Adjuvant System was also observed for anti-HBsAg titers. The results also show the enhancement effect of a third vaccine dose compared to a two-dose regimen in eliciting an anti-CS immune response. The relationship between anti-CS immune response and protection induced against malaria is complex and not yet fully characterized, as no protective threshold has been defined. While a consistent association between higher (vs. lower) anti-CS response and protection against *P. falciparum* infection was seen in challenge studies [18], [24], [25] and in field trials with active detection of infection [6], such an association was not found with malaria disease, as captured in a passive case detection system [8]. More thorough investigation of immune responses to RTS,S/AS candidate vaccines, including in efficacy studies, are underway and may contribute to better characterisation of the relationship between the immunity induced and protection.

According to national guidelines of Gabon, Hepatitis B vaccine is administered routinely as part of the EPI programme. However, supply chain ruptures, migration, and other factors lead to a partial immunization of a large proportion of the population and almost half of the children in this study had not received any hepatitis B vaccine at recruitment. Interestingly, we found that children with prior immunization against hepatitis B had a stronger immune response post-vaccination not only against HBsAg but against CS as well. Neonatal hepatitis B vaccination may therefore be favorable to early RTS,S-induced protection against malaria in case of integration of an RTS,S-based vaccine in an EPI program that includes neonatal hepatitis B vaccination.

It is unclear how previous HBs-induced immune responses enhance the CS-specific response but it is most likely related to the covalently bound CS segment and HBs fusion protein present in RTS,S. The following non exclusive hypothetical mechanisms may occur: (i) circulating anti-HBs antibodies may be favorable to CS antigen capture by antigen-presenting cells and following T-cell and B-cell priming; (ii) HBs-primed B-cells expressing anti-HBs surface antibodies may capture the RTS,S antigen and, given their antigen presentation capacities, be efficient antigen presenting cells for CS-specific T-cell priming; (iii) HBs-specific CD4 memory T-cells induced by previous vaccination may provide more rapid T-cell help to CS-specific B cells upon antigen re-stimulation by the HBsAg present in RTS,S.

Having been previously vaccinated with at least one Hepatitis B vaccine dose was associated with increased anti-HBs responses post vaccination dose 2 and dose 3, with both RTS,S formulations, indicating the absence of exhaustion of the immune response upon repeated exposure to the HBs vaccine antigen.

As a result of the encouraging result of the present trial, given the favorable safety profile and the indication of superior immunogenicity of RTS,S/AS01_E_ compared to RTS,S/AS02_D_, further development of this vaccine candidate is ongoing. Studies aiming at confirmation of safety, further age de-escalation, schedule optimization, assessment of efficacy against malaria, and interaction of RTS,S/AS01_E_ with EPI vaccines are currently being performed in various parts of Africa.

## Supporting Information

Checklist S1 CONSORT Checklist
(0.06 MB DOC)

Protocol S1 Trial Protocol
(0.79 MB PDF)

## References

[1] Breman JG, Alilio MS, Mills A (2004). Conquering the intolerable burden of malaria: what's new, what's needed: a summary.. Am J Trop Med Hyg.

[2] Sachs J, Malaney P (2002). The economic and social burden of malaria.. Nature.

[3] Girard MP, Reed ZH, Friede M, Kieny MP (2007). A review of human vaccine research and development.. Malaria Vaccine.

[4] Malkin E, Dubovsky F, Moree M (2006). Progress towards the development of malaria vaccines.. Trends Parasitol.

[5] Alonso PL, Sacarlal J, Aponte JJ, Leach A, Macete E (2005). Duration of protection with RTS,S/AS02A malaria vaccine in prevention of Plasmodium falciparum disease in Mozambican children: Single-blind extended follow-up of a randomised controlled trial.. Lancet.

[6] Aponte JJ, Aide P, Renom M, Mandomando I, Bassat Q (2007). Safety of the RTS,S/AS02D candidate malaria vaccine in infants living in a highly endemic area of Mozambique: a double blind randomised controlled phase I/IIb trial.. Lancet.

[7] Kester KE, Cummings JF, Ofori-Anyinam O, Ockenhouse CF, Krzych U (2009). Randomized, Double-Blind, Phase 2a Trial of Falciparum Malaria Vaccines RTS,S/AS01B and RTS,S/AS02A in Malaria-Naive Adults: Safety, Efficacy, and Immunologic Associates of Protection.. J Infect Dis.

[8] Alonso PL, Sacarlal J, Aponte JJ, Leach A, Macete E (2004). Efficacy of the RTS,S/AS02A vaccine against Plasmodium falciparum infection and disease in young African children: Randomised controlled trial.. Lancet.

[9] Macete EV, Sacarlal J, Aponte JJ, Leach A, Navia MM (2007). Evaluation of two formulations of adjuvanted RTS, S malaria vaccine in children aged 3 to 5 years living in a malaria-endemic region of Mozambique: a Phase I/IIb randomized double-blind bridging trial.. Trials.

[10] Wildling E, Winkler S, Kremsner PG, Brandts C, Jenne L (1995). Malaria epidemiology in the province of Moyen Ogoov, Gabon.. Trop Med Parasitol.

[11] Sylla EH, Lell B, Kun JF, Kremser PG (2001). Plasmodium falciparum transmission intensity and infection rates in children in Gabon.. Parasitol Res.

[12] Lell B, May J, Schmidt-Ott RJ, Lehman LG, Luckner D (1999). The role of red blood cell polymorphisms in resistance and susceptibility to malaria.. Clin Infect Dis.

[13] Grobusch MP, Lell B, Schwarz NG, Gabor J, Dornemann J (2008). Intermittent preventive treatment against malaria in infants in Gabon–a randomized, double-blind, placebo-controlled trial.. J Infect Dis.

[14] Goesch JN, Schwarz NG, Decker M, Oyakhirome S, Borchert LB (2008). Socio-economic status is inversely related to bed net use in Gabon.. Malar J.

[15] United Nations Population Fund. Enquête Démographique Et De Santé Gabon 2000..

[16] Bonhoeffer J, Menkes J, Gold MS, de Souza-Brito G, Fisher M (2004). Generalized convulsive seizure as an adverse event following immunization: case definition and guidelines for data collection, analysis, and presentation.. Vaccine.

[17] Bojang KA, Milligan PM, Pinder M, Vigneron L, Alloueche A (2001). Efficacy of RTS,S/AS02 malaria vaccine against Plasmodium falciparum infection in semi-immune adult men in The Gambia: a randomised trial.. Lancet.

[18] Kester KE, McKinney DA, Tornieporth N, Ockenhouse CF, Heppner DG (2007). RTS,S Malaria Vaccine Evaluation Group. A phase I/IIa safety, immunogenicity, and efficacy bridging randomized study of a two-dose regimen of liquid and lyophilized formulations of the candidate malaria vaccine RTS,S/AS02A in malaria-naïve adults.. Vaccine.

[19] Doherty JF, Pinder M, Tornieporth N, Carton C, Vigneron L (1999). A phase I safety and immunogenicity trial with the candidate malaria vaccine RTS,S/SBAS2 in semi-immune adults in The Gambia.. Am J Trop Med Hyg.

[20] Macete E, Aponte JJ, Guinovart C, Sacarlal J, Ofori-Anyinam O (2007). Safety and immunogenicity of the RTS,S/AS02A candidate malaria vaccine in children aged 1–4 in Mozambique.. Trop Med Int Health.

[21] Stewart VA, McGrath SM, Walsh DS, Davis S, Hess AS (2006). Pre-clinical evaluation of new adjuvant formulations to improve the immunogenicity of the malaria vaccine RTS,S/AS02A.. Vaccine.

[22] Polhemus ME, Remich SA, Ogutu BR, Waitumbi JN, Otieno L (2009). Evaluation of RTS,S/AS02A and RTS,S/AS01B in adults in a high malaria transmission area.. PLoS One.

[23] Pichichero ME (1996). Acellular pertussis vaccines. Towards an improved safety profile.. Drug Saf.

[24] Stoute JA, Kester KE, Krzych U, Wellde BT, Hall T (1998). Long term efficacy and immune responses following immunization with the RTS,S malaria vaccine.. J Infect Dis.

[25] Kester KE, Cummings JF, Ockenhouse CF, Nielsen R, Hall BT (2008). RTS,S Malaria Vaccine Evaluation Group. Phase 2a trial of 0, 1, and 3 month and 0, 7, and 28 day immunization schedules of malaria vaccine RTS,S/AS02 in malaria-naïve adults at the Walter Reed Army Institute of Research.. Vaccine.

