# Supplementary material for: A Randomized Trial Assessing the Safety and Immunogenicity of AS01 and AS02 Adjuvanted RTS,S Malaria Vaccine Candidates in Children in Gabon
Source: PLoS One. 2009 Oct 27;4(10):e7611. doi: 10.1371/journal.pone.0007611 (PMC2763199; doi:10.1371/journal.pone.0007611)
Supplement: Protocol S1 — Trial Protocol (0.79 MB PDF) [file pone.0007611.s002.pdf]

GlaxoSmithKline Biologicals
Rue de l'Institut 89, 1330 Rixensart,
Belgium

**Study vaccines**	GlaxoSmithKline Biologicals' candidate *Plasmodium falciparum* malaria vaccine RTS,S/AS01E (0.5 mL dose) and GlaxoSmithKline Biologicals' candidate *Plasmodium falciparum* malaria vaccine RTS,S/AS02D (0.5 mL dose)
**eTrack study number**	105874
**eTrack abbreviated title**	Malaria-046
**Investigational New Drug (IND) number**	***BB-IND 12937***
**Date of approval**	Final Version 07 December 2005
Administrative Change 1	***Final Version 16 February 2006***
**Title**	A Phase II randomized, double-blind bridging study of the safety and immunogenicity of GlaxoSmithKline Biologicals' candidate *Plasmodium falciparum* malaria vaccine RTS,S/AS01E (0.5 mL dose) to RTS,S/AS02D (0.5 mL dose) administered IM according to a 0, 1, 2-month vaccination schedule in children aged 18 months to 4 years living in Gabon.

GlaxoSmithKline Biologicals

Co-ordinating author Conor Cahill, Scientific Writer

Contributing authors

- W Ripley Ballou, Vice President – Clinical R&D, Early Development
- Sarah Benns, Scientific Writer
- Ozzie Berger, US Regulatory Affairs
- Joe Cohen, Vice President R&D – Vaccines For Emerging Diseases Program
- Marie-Ange Demoitié, Clinical Read-Outs Manager; R&D: Vaccines for Emerging Diseases Program
- Marie Claude Dubois, Project Manager – Disease Area Programme Vaccines for Emerging Diseases
- Amanda Leach, Clinical Development Manager
- Marc Lievens, Statistician
- Philip Moris, Scientist, Human CMI
- Ally Olotu, WHO Trainee
- Isabelle Ramboer, Central Study Coordinator
- Joelle Thonnard, Manager, Clinical Safety & Pharmacovigilance
- Marie Chantal Uwamwezi, Scientist – Regulatory Development Unit III
- Johan Vekemans, Clinical Development Manager

GSK Biologicals' Protocol DS V 12.2

Copyright 2005 the GlaxoSmithKline group of companies. All rights reserved.
Unauthorized copying or use of this information is prohibited.

**eTrack study number**	105874
**eTrack abbreviated title**	Malaria-046
**IND number**	***BB-IND 12937***
**Date of approval**	Final Version 07 December 2005
Administrative Change 1	***Final Version 16 February 2006***
**Title**	A Phase II randomized, double-blind bridging study of the safety and immunogenicity of GlaxoSmithKline Biologicals' candidate *Plasmodium falciparum* malaria vaccine RTS,S/AS01E (0.5 mL dose) to RTS,S/AS02D (0.5 mL dose) administered IM according to a 0, 1, 2-month vaccination schedule in children aged 18 months to 4 years living in Gabon.

Albert Schweitzer Hospital, Medical Research Unit Lambaréné, Gabon, Contributing Authors

- Saadou Issifou, Head of Unit
- Bertrand Lell, Principal Investigator
- Michel Anoumou Missinou, Quality Contol Officer
- Benjamin Mordmüller, Study Immunologist

University of Tubingen, School of Tropical Medicine, Tubingen, Germany Contributing Author

- Peter Kremsner, Program Coordinator

Malaria Vaccine Initiative at Program for Appropriate Technology in Health Contributing Authors

- Filip Dubovsky
- Aric Gregson
- Carolyn Petersen
- Marla Silman

**eTrack study number**	105874
**eTrack abbreviated title**	Malaria-046
**IND number**	***BB-IND 12937***
**Date of approval**	Final Version 07 December 2005
Administrative Change 1	***Final Version 16 February 2006***
**Title**	A Phase II randomized, double-blind bridging study of the safety and immunogenicity of GlaxoSmithKline Biologicals' candidate *Plasmodium falciparum* malaria vaccine RTS,S/AS01E (0.5 mL dose) to RTS,S/AS02D (0.5 mL dose) administered IM according to a 0, 1, 2-month vaccination schedule in children aged 18 months to 4 years living in Gabon.

Sponsor signatory approval

~~**Sponsor signatory:**~~	~~Johan Vekemans~~<br>~~Clinical Development Manager~~
~~**Signature:**~~	______________________________
~~**Date:**~~	______________________________

eTrack study number 105874

eTrack abbreviated title Malaria-046

IND number ***BB-IND 12937***

1. Principal Investigator

Betrand Lell

2. Medical Monitor

Johan Vekemans,
Clinical Development Manager,
GlaxoSmithKline Biologicals,
Rue de l'Institut 89, 1330 Rixensart,
Belgium.
Tel: +32.2.656.56.59
Fax: +32.2.656.80.44
email: johan.vekemans@gskbio.com

3. Central Study Coordinator

Isabelle Ramboer,
Central Study Coordinator,
GlaxoSmithKline Biologicals,
Rue de l'Institut 89, 1330 Rixensart,
Belgium.
Tel: +32.2.656.68.20
Fax: +32.2.656.80.44
email: isabelle.ramboer@gskbio.com

eTrack study number 105874

eTrack abbreviated title Malaria-046

IND number ***BB-IND 12937***

4. Study Contact for Reporting of a Serious Adverse Event

All four of the contacts listed below must be informed of each SAE

Study Contact at GSK Biologicals for Reporting Serious Adverse Events Manager Clinical Safety Vaccines

GSK Biologicals Clinical Safety Physician,
GlaxoSmithKline Biologicals,
Rue de l'Institut 89, 1330 Rixensart,
Belgium.
Tel: +32.2.656.87.98
Fax: +32.2.656.80.09
Mobile phone for 7/7 day availability:
+32.477.40.47.13
email: rix.ct-safety-vac@gskbio.com

Central Study Coordinator

Isabelle Ramboer,
Central Study Coordinator,
GlaxoSmithKline Biologicals,
Rue de l'Institut 89, 1330 Rixensart,
Belgium.
Tel: +32.2.656.68.20
Fax: +32.2.656.80.44
email: isabelle.ramboer@gskbio.com

Malaria Vaccine Initiative

Carolyn Petersen,
Malaria Vaccine Initiative, PATH,
7500 Old Georgetown Road, 12th Floor,
Bethesda, MD 20814,
USA.
Tel: +1.240.395.27.16
or Tel: +1.240.395.27.00
Fax: +1.240.395.25.91
email: sae@malariavaccine.org

alternate email:
cpetersen@malariavaccine.org

Local Safety Monitor

~~Jenny Dörnemann,~~ Grégoire Adzoda
Hôpital Albert Schweitzer,
B.P. ~~118, Lambaréné,~~ ***13901, Libreville***
Gabon.

Tel: ~~+241.077.79.225~~
+00 241 07 35 83 33.
Fax: +241.581.196
~~email: jennydoernemann@gmx.de~~
gadzoda@yahoo.fr

Administrative Change, 16 February 2006

**eTrack study number**	105874
**eTrack abbreviated title**	Malaria-046
**IND number**	***BB-IND 12937***

5. Study Contact for Emergency Code Break

GSK Biologicals Clinical Safety Physician,
GlaxoSmithKline Biologicals,
Rue de l'Institut 89, 1330 Rixensart,
Belgium.
Tel: +32.2.656.87.98
Fax: +32.2.656.80.09
Mobile phone for 7/7 day availability: +32.477.40.47.13

6. Study Center

Laboratoire de Recherches
Hôpital Albert Schweitzer
B.P.118
Lambaréné, Gabon

Investigator Agreement

**eTrack study number**	105874
**eTrack abbreviated title**	Malaria-046
**IND number**	***BB-IND 12937***
**Title**	A Phase II randomized, double-blind bridging study of the safety and immunogenicity of GlaxoSmithKline Biologicals' candidate *Plasmodium falciparum* malaria vaccine RTS,S/AS01E (0.5 mL dose) to RTS,S/AS02D (0.5 mL dose) administered IM according to a 0, 1, 2-month vaccination schedule in children aged 18 months to 4 years living in Gabon.

I agree:

- To assume responsibility for the proper conduct of the study at this site.
- To conduct the study in compliance with this protocol, any mutually agreed future protocol amendments, and with any other study conduct procedures provided by GlaxoSmithKline Biologicals (GSK Biologicals).
- To ensure that all persons assisting me with the study are adequately informed about the GSK Biologicals investigational product(s) and other study-related duties and functions as described in the protocol.
- Not to implement any changes to the protocol without agreement from the sponsor and prior review and written approval from the Institutional Review Board (IRB) or Independent Ethics Committee (IEC), except where necessary to eliminate an immediate hazard to the subjects, or where permitted by all applicable regulatory requirements (for example, for administrative aspects of the study).
- That I am thoroughly familiar with the appropriate use of the vaccine(s), as described in this protocol, and any other information provided by the sponsor, including, but not limited to, the following: the current Investigator's Brochure (IB) or equivalent document, IB supplement (if applicable), prescribing information (in the case of a marketed vaccine).
- That I am aware of, and will comply with, "Good Clinical Practice" (GCP) and all applicable regulatory requirements.
- To ensure that all persons assisting me with the study are adequately informed about the GSK Biologicals investigational product(s) and other study-related duties and functions as described in the protocol.
- That I have been informed that certain regulatory authorities require the sponsor to obtain and supply, as necessary, details about the investigator's ownership interest in the sponsor or the investigational product, and more generally about his/her financial ties with the sponsor. GSK Biologicals will use and disclose the information solely for the purpose of complying with regulatory requirements.

Hence I:

- Agree to supply GSK Biologicals with any necessary information regarding ownership interest and financial ties (including those of my spouse and dependent children).
- Agree to promptly update this information if any relevant changes occur during the course of the study and for 1 year following completion of the study.
- Agree that GSK Biologicals may disclose any information it has about such ownership interests and financial ties to regulatory authorities.
- Agree to provide GSK Biologicals with an updated Curriculum Vitae and other FDA required documents.

Investigator name: Bertrand Lell

~~Investigator signature~~ **~~Date~~**

Synopsis

Title A Phase II randomized, double-blind bridging study of the safety and immunogenicity of GlaxoSmithKline Biologicals' candidate *Plasmodium falciparum* malaria vaccine RTS,S/AS01E (0.5 mL dose) to RTS,S/AS02D (0.5 mL dose) administered IM according to a 0, 1, 2-month vaccination schedule in children aged 18 months to 4 years living in Gabon.

Indication/Study population Primary immunization of healthy male and female children aged 18 months to 4 years at enrolment, if eligible according to inclusion and exclusion criteria.

Rationale The RTS,S/AS01E candidate malaria vaccine is being developed for the routine immunization of infants living in malaria-endemic areas as part of the Expanded Program of Immunization (EPI). The RTS,S/AS01E candidate malaria and hepatitis B vaccine consists of sequences of the circumsporozoite (CS) protein and hepatitis B surface antigen (HBsAg) with the proprietary adjuvant AS01E (proprietary liposomes, MPL® and QS21 immunostimulants).

Previous malaria vaccine studies in children have been conducted with the same antigen, but administered with an adjuvant formulation from the AS02 adjuvant family which consists of an oil-in-water emulsion, MPL and QS21. The following table details the various vaccine formulations that have been trialled in humans (or are planned for human trials).

	Freeze-dried fraction	Liquid fraction				
**Formulation**	**RTS,S (µg)**		**MPL (µg)**	**QS21 (µg)**	**Dose volume**	
RTS,S/AS02A (0.5 mL dose)	50	Oil-in-water emulsion	50	50	0.5 mL	Efficacy against infection, Gambian adults (Malaria-005)
RTS,S/AS02A (0.25 mL dose)	25	Oil-in-water emulsion	25	25	0.25 mL	Efficacy against clinical disease, Mozambican children (Malaria-026)
RTS,S/AS02D	25	Oil-in-water emulsion	25	25	0.5 mL	Pediatric formulation (Malaria-034, -038, -040)
RTS,S/AS01B	50	Liposomes	50	50	0.5 mL	Efficacy in challenge model (Malaria-027)
RTS,S/AS01E	25	Liposomes	25	25	0.5 mL	Proposed pediatric formulation

The RTS,S/AS02A vaccine (0.25 mL dose) has demonstrated efficacy against infection and a range of illnesses caused by *P. falciparum* in children aged 1 to 4 years in Mozambique (Malaria-026). Up to 18 months post Dose 3 the VE determined as the time to the first clinical episode of malaria was 33%. VE against multiple attacks of malaria was 32% and against severe malaria was 49% over the 18 months of the trial.

The RTS,S/AS01 vaccines have been developed in parallel with the RTS,S/AS02 vaccines with the aim of improving the immune response and increasing vaccine efficacy. A recent challenge study with RTS,S/AS01B in malaria-naïve adults (Malaria-027) has shown encouraging results, indicating a similar safety profile to that of RTS,S/AS02A, higher humoral immunogenicity, a favorable Th1 cell-mediated immune profile and a trend towards higher vaccine efficacy.

RTS,S/AS01E (0.5 mL dose) is the proposed pediatric formulation which is composed of the same active constituents in the same quantities as a 0.25 mL dose of RTS,S/AS01B.

In light of those promising results, a study comparing the safety, immunogenicity and efficacy of RTS,S/AS02A with RTS,S/AS01B is underway in Kenyan adults (Malaria-044; BB-IND11220). Final study data reviewed by the Data Safety Monitoring Board from this trial will be available prior to starting Malaria-046. Age de-escalation will proceed with the proposed study which will be the first study to examine the administration of RTS,S/AS01E to children.

This trial is designed to evaluate safety, reactogenicity and immunogenicity of RTS,S antigen formulated in the adjuvant AS01E. The comparator group will receive RTS,S/AS02D.

This product development plan is conducted under a partnership agreement with the Malaria Vaccine Initiative at PATH (MVI) and is guided by a joint MVI/GSK Steering Committee.

This study is overseen by a formally constituted DSMB operating under a charter.

Objectives **Coprimary**

- For RTS,S/AS01E when administered as 3 doses intramuscularly on a 0, 1, 2-month schedule to children aged 18 months to 4 years living in a malaria-endemic area:
  - to assess safety until one month post Dose 3

- to demonstrate non-inferiority to RTS,S/AS02D in terms of anti-CS antibody response one month post Dose 3.

Secondary

- For RTS,S/AS01E when administered as 3 doses intramuscularly on a 0, 1, 2-month schedule to children aged 18 months to 4 years living in a malaria-endemic area:
  - to assess reactogenicity until one month post Dose 3
  - to demonstrate non-inferiority to RTS,S/AS02D in terms of anti-HBs antibody response one month post Dose 3
  - to describe seroprotection against hepatitis B up to one month post Dose 3
  - to describe the anti-CS response up to one month post Dose 3.

Tertiary

- To assess, for RTS,S/AS01E when administered as 3 doses intramuscularly on a 0, 1, 2-month schedule to children aged 18 months to 4 years living in a malaria-endemic area:
  - safety between 1 month post Dose 3 until 12 months post Dose 3.
  - humoral immune response to CS antigen at 12 months post Dose 3
  - humoral immune response to HBs antigen at 12 months post Dose 3.

Exploratory

- For RTS,S/AS01E and RTS,S/AS02D when administered as 3 doses intramuscularly on a 0, 1, 2-month schedule to children aged 18 months to 4 years living in a malaria-endemic area:
  - to evaluate T-cell-mediated immune response (CMI) to CS antigen up to 12 months post Dose 3
  - to evaluate B-cell memory immune response to CS antigen up to 12 months post Dose 3
  - to describe the anti-CS response up to one month post Dose 3 according to documented HBV immunization status at screening

Study design

- Experimental design: Phase II, single center, double-blind (observer blind, participant blind), randomized (1:1 ratio) trial with two groups in one study site.

- Healthy male and female children aged 18 months to 4 years of age will be screened; those determined to be eligible, based on the inclusion and exclusion criteria, will be enrolled in the study.
- Route of administration: all vaccines will be administered by the intramuscular route to the left deltoid.
- Each child will be observed for at least 60 minutes after vaccination to evaluate and treat any acute adverse events (AEs).
- There will be a 7-day follow-up period for solicited AEs post-vaccination: Day 0 evaluation will be carried out by the study physician at the study center. Subsequently, trained field workers will visit the children to solicit AEs on days 1 to 6 after each vaccination (evaluation on Day 6 post Dose 1 will be carried out by the study physician at the study center).
- There will be a 30-day (day of vaccination and 29 subsequent days) follow-up after each vaccine dose for reporting unsolicited symptoms.
- Serious adverse events (SAEs) will be recorded throughout the study period. Prior to vaccination, any SAEs due directly to study procedures will be captured. All SAEs will be captured, beginning with the administration of the first dose and ending 14 months after Dose 1 of study vaccine. After the double-blind phase of the study, all enrolled children will be visited at home monthly by field workers until study conclusion to ensure complete identification of all SAEs.
- Safety review process: safety data from the first 40 children to receive Dose 1 and Dose 2 will be reviewed by the DSMB to authorize progression to the next sequential dose.
- This study will not capture cases of malaria for the analysis of efficacy. All cases of malaria presenting during the unsolicited period will be captured as unsolicited events. Cases at anytime during the study that meet the criteria for a SAE will be reported as SAEs.
- The access of the study population to HIV voluntary counseling and testing and HIV antiretroviral therapy according to national recommendations will be ensured, in collaboration with the government services.
- An insecticide impregnated bednet will be offered to all parent(s)/guardian(s) who present their child for screening.
- Blood for safety monitoring of hematology, renal and hepatic function will be measured at screening, one week post Dose 1, one month post Dose 3 and 12 months post Dose 3.

- Anti-CS antibody titers will be determined at screening, one month post Dose 2, one month post Dose 3 and 12 months post Dose 3.
- Anti-HBs antibody titers will be determined at screening, one month post Dose 2, one month post Dose 3 and 12 months post Dose 3.
- CMI and B-cell memory immune responses will be determined at screening, one month post Dose 2, one month post Dose 3 and 12 months post Dose 3.
- Timing of final analysis: 1 month post Dose 3. An annex safety analysis will be performed at the end of the study, one year post Dose 3.
- Data collection: conventional Case Report Form (CRF).

Number of subjects

180 subjects will be enrolled. It is expected that approximately 150 subjects will be evaluable at study end.

Primary endpoints

Safety

- Occurrence of SAEs from the time of first vaccination (Study Month 0) until one month post Dose 3 (Study Month 3).

Immunogenicity

- Anti-CS antibody titers one month post Dose 3.

Secondary endpoints

Safety and Reactogenicity

- Occurrence of solicited general and local reactions over a 7-day follow-up period (day of vaccination and 6 subsequent days) after each vaccination.
- Occurrence of unsolicited symptoms after each vaccination over a 30-day follow-up period (day of vaccination and 29 subsequent days).

Immunogenicity

- Anti-CS antibody titers prior to vaccination, one month post Dose 2 and one month post Dose 3.
- Anti-HBs antibody titers prior to vaccination, one month post Dose 2 and one month post Dose 3.

**Tertiary endpoints**	**Immunogenicity**<br><br>*At Study Month 14:*<br><br>• Anti-HBs antibody titers determined 12 months post Dose 3.<br><br>• Anti-CS antibody titers determined at 12 months post Dose 3.<br><br>**Safety**<br><br>• Occurrence of SAEs from 1 month post Dose 3 until 12 months post Dose 3.
**Exploratory endpoints**	**Cell-Mediated Immunity**<br><br>• Frequency of CS-specific T-cells prior to vaccination, one month post Dose 2, one month post Dose 3 and 12 months post Dose 3.<br><br>• Frequency of CS-specific B-cell memory prior to vaccination, one month post Dose 2, one month post Dose 3 and 12 months post Dose 3.<br><br>• Anti-CS antibody titers determined at 12 months post Dose 3 according to documented HBV immunization status at screening

TABLE OF CONTENTS

PAGE

SYNOPSIS **9**

LIST OF ABBREVIATIONS **19**

GLOSSARY OF TERMS **22**

1. INTRODUCTION **25**
1.1. Malaria 25
1.2. Hepatitis 25
1.3. RTS,S candidate vaccine 26
1.3.1. AS02 and AS01 adjuvants 26
1.4. The RTS,S/AS02 candidate malaria vaccine; key clinical efficacy, safety and immunogenicity data 27
1.5. The RTS,S/AS01B candidate malaria vaccine; preliminary clinical safety, efficacy and immunogenicity data 29
1.6. Rationale for the study design 30
1.6.1. Rationale for CMI and B-cell memory testing 30

2. OBJECTIVES **31**
2.1. Coprimary objectives 31
2.2. Secondary objectives 31
2.3. Tertiary objectives 32
2.4. Exploratory Objectives 32

3. STUDY DESIGN OVERVIEW **32**

4. STUDY COHORT **33**
4.1. The Albert Schweitzer Hospital Medical Research Unit 33
4.2. Geography and study population 34
4.3. Malaria epidemiology and control in the study area 35
4.4. Healthcare in the study area 35
4.4.1. HIV services 36
4.5. Number of subjects 36
4.6. Inclusion criteria for enrolment 36
4.7. Exclusion criteria for enrolment 36
4.8. Elimination criteria during the study 37
4.9. Contraindications to subsequent vaccination 38
4.9.1. Indications for deferral of vaccination 38
4.9.2. Absolute contraindications to further vaccination 38

5. CONDUCT OF STUDY **39**
5.1. Ethics and regulatory considerations 39
5.1.1. Institutional Review Board/Independent Ethics Committee (IRB/IEC) 39
5.1.2. Informed consent 40
5.1.3. Safety monitoring plan 42
5.1.3.1. Data Safety Monitoring Board (DSMB) 43
5.1.3.1.1. Data Reviewed by the DSMB 43

5.1.3.2. Local Safety Monitor (LSM) 43
5.1.3.2.1. Data Reviewed by the LSM 44
5.1.3.3. Safety monitoring reports 44
5.1.3.4. Process for the suspension of progression to the next sequential vaccine dose 45
5.1.3.5. Process if the trial is suspended on the basis of safety reports on first 40 subjects to receive each of the first two vaccine doses 45
5.2. Storage of study documentation at investigator's site 46
5.3. Recruitment/Screening 46
5.3.1. Community information 46
5.3.2. Screening of volunteers 46
5.4. Vaccination process 47
5.5. Home follow-up visits for assessment of reactogenicity (7-day follow-up period) 48
5.6. Monitoring of hematological and biochemical laboratory parameters 49
5.7. Surveillance for SAEs (all subjects) 49
5.7.1. At health facilities 49
5.7.2. In the community 50
5.8. Provision of health care 50
5.8.1. Management and treatment of malaria in all subjects 51
5.8.1.1. Insecticide impregnated bednets 51
5.9. Subject identification 52
5.10. Outline of study procedures 53
5.11. Detailed description of study stages/visits 54
5.12. Sample handling and analysis 58
5.12.1. Treatment and storage of biological samples 58
5.12.2. Laboratory assays 58
5.12.3. Serology, CMI and B-cell memory plan 59

6. INVESTIGATIONAL PRODUCTS AND ADMINISTRATION 60
6.1. Study vaccines 60
6.1.1. RTS,S/AS01E vaccine 61
6.1.1.1. RTS,S antigen presentation: 61
6.1.1.2. AS01E adjuvant: 61
6.1.2. RTS,S/AS02D vaccine 61
6.1.2.1. RTS,S antigen presentation: 61
6.1.2.2. AS02D adjuvant: 62
6.2. Dosage and administration 62
6.2.1. RTS,S/AS01E 62
6.2.2. RTS,S/AS02D 62
6.3. Storage 63
6.4. Treatment allocation and randomization 63
6.4.1. Randomization of supplies 63
6.4.2. Randomization of subjects 63
6.5. Method of blinding and breaking the study blind 63
6.6. Replacement of unusable vaccine doses 64
6.7. Packaging 64
6.8. Vaccine accountability 64
6.9. Concomitant medication/treatment 64

7. HEALTH ECONOMICS 65

8. ADVERSE EVENTS AND SERIOUS ADVERSE EVENTS 65
8.1. Definition of an adverse event..66
8.2. Definition of a serious adverse event 66
8.3. Clinical laboratory parameters and other abnormal assessments qualifying as adverse events and serious adverse events..........................67
8.4. Time period, frequency, and method of detecting adverse events and serious adverse events 68
8.4.1. Solicited adverse events 69
8.5. Evaluating adverse events and serious adverse events.............................69
8.5.1. Assessment of intensity 69
8.5.2. Assessment of causality 71
8.6. Follow-up of adverse events and serious adverse events and assessment of outcome 73
8.7. Prompt reporting of serious adverse events to GSK Biologicals.................74
8.7.1. Time frames for submitting serious adverse event reports to GSK Biologicals 74
8.7.2. Completion and transmission of serious adverse event reports to GSK Biologicals 74
8.8. Regulatory reporting requirements for serious adverse events 75
8.9. Post-study adverse events and serious adverse events.............................76
8.10. Pregnancy 76
8.11. Treatment of adverse events 76

9. SUBJECT COMPLETION AND WITHDRAWAL..76
9.1. Subject completion 76
9.2. Subject withdrawal..76
9.2.1. Subject withdrawal from the study 77
9.2.2. Subject withdrawal from investigational product..........................77

10. DATA EVALUATION: CRITERIA FOR EVALUATION OF OBJECTIVES............78
10.1. Coprimary endpoints...78
10.1.1. Safety 78
10.1.2. Immunogenicity...78
10.2. Secondary endpoints 78
10.2.1. Safety and reactogenicity..78
10.2.2. Immunogenicity...78
10.3. Tertiary endpoints 78
10.3.1. Immunogenicity...78
10.3.2. Safety 78
10.4. Exploratory endpoints 79
10.5. Estimated sample size 79
10.5.1. Sample size for the primary safety endpoint 79
10.6. Study cohorts to be evaluated...79
10.6.1. Total Vaccinated Cohort 79
10.6.2. According to protocol (ATP) cohort for analysis of safety 80
10.6.3. According to protocol (ATP) cohort for analysis of immunogenicity...80
10.7. Derived and transformed data...80
10.8. Final analyses...80
10.8.1. Analyses of safety..81
10.8.1.1. Primary endpoint 81
10.8.1.2. Secondary safety endpoints 81

10.8.2. Analyses of immunogenicity....82
10.8.2.1. Anti-CS antibodies....82
10.8.2.2. Anti-HBs antibodies....82
10.8.3. Exploratory analysis....82
10.9. Annex analysis at study conclusion....82
10.9.1. Analyses of safety....82
10.9.2. Analyses of immunogenicity....83
10.9.2.1. Anti-HBs antibodies....83
10.9.2.2. Anti-CS antibodies....83
10.9.2.3. Exploratory analysis....83
10.10. Planned interim analysis....83

11. ADMINISTRATIVE MATTERS....83

12. REFERENCES....84

APPENDICES

Appendix A World Medical Association Declaration of Helsinki....89

Appendix B Administrative Matters....93

Appendix C Overview of the Recruitment Plan....99

Appendix D Handling of Biological Samples Collected by the Investigator....100

Appendix E Shipment of Biological Samples....103

Appendix F Laboratory Assays....106

Appendix G Vaccine supplies, packaging and accountability....107

Appendix H Administrative change to the protocol....109

List of Abbreviations

AE	Adverse event
ALT	Alanine aminotransferase
anti-CS	Antibody to the *P. falciparum* circumsporozoite (CS) repeat domain
anti-HBsAg	Antibody to the hepatitis B surface antigen
ATP	According to protocol
CI	Confidence interval
CMI	Cell-mediated immunity
CRA	Clinical research associate
CRF	Case report form
CS	Circumsporozoite protein of *P. falciparum*
CSC	Central study coordinator
DSMB	Data safety monitoring board
EIA	Enzyme immunosorbent assay
EIR	Entomological inoculation rate
EISR	Expedited investigator safety report
ELISA	Enzyme linked immunosorbent assay
EPI	Expanded program on immunization
FDA	Food and Drug Administration, United States
GCP	Good clinical practice
GMT	Geometric mean titer
GSK	GlaxoSmithKline
HAS	Albert Schweitzer Hospital (Hôpital Albert Schweitzer)
HBs	Hepatitis B surface antibody
HBsAg	Hepatitis B surface antigen

HBV	Hepatitis B virus
HIV	Human immunodeficiency virus
IB	Investigator's brochure
ICF	Informed consent form
IEC	Independent ethics committee
IFN-γ	Interferon gamma
IM	Intramuscular
IND	Investigational new drug
IRB	Institutional review board
IU	International unit
ITN	Insecticide-treated bednet
kg	Kilogram
LSM	Local safety monitor
MedDRA	Medical Dictionary for Regulatory Activities
mg	Milligram
mL	Milliliter
MPL®	3-deacylated monophosphoryl lipid A
MRU	Medical Research Unit, Albert Schweitzer Hospital
MVI	Malaria Vaccine Initiative
P. falciparum	*Plasmodium falciparum*
PATH	Program for Appropriate Technology in Health
PFS	Pre-filled syringe
PPP	Pre-patent period
QS 21	'*Quillaja saponaria* 21': a triterpene glycoside purified from the bark of the soap bark tree, *Quillaja saponaria*
RAP	Report and Analysis Plan

RTS	Hybrid protein comprising HBs (hepatitis B surface antigen) and CSP portions
RTS,S	Particulate antigen, containing both RTS and HBs proteins
SAE	Serious adverse event
SOP	Standard operating procedure
SP	sulfadoxine-pyrimethamine
ULN	Upper Limit of Normal
VCT	Voluntary Counseling and Testing
VE	Vaccine efficacy
WHO	World Health Organization
WRAIR	Walter Reed Army Institute of Research

Glossary of Terms

Adverse event:	Any untoward medical occurrence in a patient or clinical investigation subject, temporally associated with the use of a medicinal product, whether or not considered related to the medicinal product.<br><br>An AE can therefore be any unfavorable and unintended sign (including an abnormal laboratory finding), symptom, or disease (new or exacerbated) temporally associated with the use of a medicinal product. For marketed medicinal products, this also includes failure to produce expected benefits (i.e. lack of efficacy), abuse or misuse.
Blinding:	A procedure in which one or more parties to the trial are kept unaware of the treatment assignment in order to reduce the risk of biased study outcomes. In a single-blind trial, the investigator and/or his staff are aware of the treatment assignment but the subject is not. In an observer-blind study, the subject and the study personnel involved in the clinical evaluation of the subjects are blinded while other study personnel may be aware of the treatment allocation. When the investigator and sponsor staff who are involved in the treatment or clinical evaluation of the subjects and review/analysis of data are also unaware of the treatment assignments, the study is double blind. Partially blind is to be used for study designs with different blinding levels between different groups, e.g. double blinded consistency lots which are open with respect to the control group. The level of blinding is maintained throughout the conduct of the trial, and only when the data are cleaned to an acceptable level of quality will appropriate personnel be unblinded or when required in case of a serious adverse event.
Central Study Coordinator:	An individual assigned by and centrally located at GSK Biologicals at Rixensart who is responsible for assuring proper conduct of a clinical study.
Data Safety Monitoring Board (DSMB):	The DSMB is an independent committee appointed to oversee ethical and safety aspects of the conduct of the study. See Section 5.1.3.1 for a full overview of the role and structure of the DSMB.
Eligible:	Qualified for enrollment into the study based upon strict adherence to inclusion/exclusion criteria.
eTrack	GSK's clinical trials tracking tool.

Evaluable:	Meeting all eligibility criteria, complying with the procedures defined in the protocol, and, therefore, included in the according-to-protocol (ATP) analysis (see Sections 4.7, 4.8 and 10.6 for details on criteria for evaluability).
Investigational product:	A pharmaceutical form of an active ingredient or placebo being tested or used as a reference in a clinical trial, including a product with a marketing authorization when used in a way different from the approved form, or when used for an unapproved indication, or when used to gain further information about an approved use. The investigational products for this study are RTS,S/AS02D and RTS,S/AS01E.
Local Safety Monitor (LSM):	The overall role of the Local Safety Monitor, an experienced physician based in-country, will be to support the study investigators and to act as a link between the investigators and the Data Safety Monitoring Board (DSMB) (see Section 5.1.3.1 for further details on the LSM).
Medical Monitor:	An individual medically qualified to assume the responsibilities of the sponsor (GSK Biologicals) especially in regards to the ethics, clinical safety of a study and the assessment of adverse events.
Pre-patent Period (PPP)	The time in days between experimental sporozoite challenge and first detection of parasitemia by peripheral blood thick smear.
Protocol amendment:	ICH defines a protocol amendment as: "A written description of a change(s) to or formal clarification of a protocol". GSK Biologicals further details this to include a change to an approved protocol that affects the safety of subjects, scope of the investigation, study design, or scientific integrity of the study.
Protocol administrative change:	A protocol administrative change addresses changes to only logistical or administrative aspects of the study.<br><br>N.B. Any change that falls under the definition of a protocol amendment (e.g. a change that affects the safety of subjects, scope of the investigation, study design, or scientific integrity of the study) MUST be prepared as an amendment to the protocol.
Randomization:	Process of random attribution of treatment to subjects in order to reduce bias of selection.
Solicited adverse event:	Adverse events (AEs) to be recorded as endpoints in the clinical study. The presence/occurrence/intensity of these events is actively solicited from the subject or an observer during a specified post-vaccination follow-up period.

Study Monitor:	An individual assigned by the sponsor who is responsible for assuring proper conduct of a clinical study.
Subject:	Term used throughout the protocol to denote an individual that has been contacted in order to participate in the clinical study, either as a recipient of the investigational product(s) or as a control.
Treatment:	Term used throughout the clinical study to denote a set of investigational products or marketed products intended to be administered to a subject, identified by a unique number, according to the study randomization or treatment allocation.
Treatment number:	A unique number identifying a treatment to a subject, according to the study randomization or treatment allocation.
Unsolicited adverse event:	Any adverse event (AE) reported in addition to those solicited during the clinical study. Also any “solicited” symptom with onset outside the specified period of follow-up for solicited symptoms will be reported as an unsolicited adverse event.

1. INTRODUCTION

1.1. Malaria

Four species of the *Plasmodium* protozoan parasite are the etiologic agents of malaria in humans (*P. falciparum*, *P. vivax*, *P. ovale* and *P. malariae*). Of these four parasites, *P. falciparum* is the major cause of severe morbidity and mortality.

There can be no doubt of the importance of *P. falciparum* malaria as a major cause of human suffering and economic drain across sub-Saharan Africa [Breman 2001a; Gallup 2001]. In this region, it causes the deaths of between 0.5 and 2.0 million children every year and is the most common reason for admission to hospital, leading each year to about 300 million clinical episodes in children under five years [Breman 2001a].

The incidence of malaria in much of Africa is increasing for a variety of reasons: changes in agricultural practices, armed conflicts, migration of refugees, increasing drug resistance to conventional anti-malarial drugs, and insecticide resistance of the anopheline mosquito vectors. It is estimated that the number of cases of clinical malaria will more than double over the next 20 years without effective control. The burden of malaria at the country level correlates closely with the rate of economic development even after adjustment for confounding factors, indicating that malaria is an important constraint on economic progress [Breman 2001b].

Clinical manifestations of *P. falciparum* disease appear as a result of the parasite infection of the red blood cell (RBC). Initial symptoms may include fever, chills, headache, joint and muscle pain, sweating, and vomiting. Acute complications may result from hemolysis leading to anemia and the propensity of infected RBCs to become adhesive and to be sequestered in capillaries thus causing local inflammatory reactions and damage to vital organs, leading to cerebral, hepatic, renal or pulmonary malaria. In *P. falciparum* malaria, an untreated acute attack can progress very rapidly and death may occur within a short timeframe.

Efforts to develop vaccines that target each stage of the parasite life cycle, to identify protective antigens and to understand the nature of the protective immune responses have been ongoing for the past three decades. The approach of GlaxoSmithKline (GSK) Biologicals has been to focus on vaccines that target the free sporozoite and intra-hepatic stages of the parasites (i.e. the pre-erythrocytic stages).

1.2. Hepatitis

Hepatitis B is an infection of the liver due to hepatitis B virus (HBV); it is an important public health problem across the developing world. World-wide approximately 350 million people carry HBV and about 1 million chronic carriers die annually [Vryheid 2001]. The likelihood of an infection becoming chronic is dependent upon the age at infection: 90% if infected in infancy, 30% to 50% if infected between the ages of 1 to 4 years, and low in adulthood. For those that become chronically infected during childhood

the risk of death from HBV-related liver cancer or cirrhosis in adult life is approximately 25% [World Health Organization 2003].

1.3. RTS,S candidate vaccine

GSK Biologicals and the Walter Reed Army Institute of Research (WRAIR) are developing a candidate antigen against malaria caused by *P. falciparum*: RTS,S. The vaccine consists of sequences of the circumsporozoite (CS) protein and the hepatitis B surface antigen (HBsAg) adjuvanted with AS02 (proprietary oil-in-water emulsion formulated with MPL® and QS21 immunostimulants) or AS01 (liposome formulation with MPL and QS21 immunostimulants).

The HBsAg contained in the RTS,S candidate malaria vaccine is encoded by the hepatitis B virus S protein gene that is identical to the gene used to express HBsAg in GSK Biologicals' Engerix-B® vaccine against hepatitis B. As a result, vaccines containing RTS,S also provide protection against hepatitis B.

In parallel to the continued development of RTS,S/AS02 in children in endemic countries, GSK Biologicals and WRAIR have continued to pursue strategies to improve the vaccine efficacy (VE) and duration of efficacy. One such strategy is the combination of the RTS,S antigen with the AS01 adjuvant. Both the AS02 and AS01 adjuvant formulations have a number of similar key components (Table 1).

Table 1 Formulations of RTS,S

Formulation	RTS,S (µg)	MPL (µg)	QS21 (µg)	Volume (mL)	Key clinical trials
RTS,S/AS02A (0.5 mL dose)	50	50	50	0.5	Efficacy demonstrated in adults in an endemic country (Malaria-005[a])
RTS,S/AS02A (0.25 mL dose)	25	25	25	0.25	Efficacy demonstrated in children in an endemic area (Malaria-026[b])
RTS,S/AS02D (0.5 mL dose)	25	25	25	0.5	Bridged to RTS,S/AS02A (0.25 mL dose) (Malaria-034[c])
RTS,S/AS01B (0.5 mL dose)	50	50	50	0.5	Efficacy demonstrated in malaria-naïve adults (Malaria-027[d])
RTS,S/AS01E (0.5 mL dose)	25	25	25	0.5	Proposed pediatric formulation

a GSK data on file, Malaria-005, Clinical Study Protocol, 1997; Bojang, 2001
b GSK data on file, Malaria-026, Clinical Study Report, 2004; Alloueche, 2004
c 0, Malaria-034, Clinical Study Report, 2004
d GSK data on file, Malaria-027, Clinical Study Protocol, 2003

1.3.1. AS02 and AS01 adjuvants

The GSK proprietary adjuvant system 2 (AS02) is composed of a proprietary oil-in-water emulsion and the immunostimulants QS21 (a triterpene glycoside purified from the bark of *Quillaja saponaria*) and MPL. RTS,S/AS02D is composed of the same active constituents in the same quantities as in a 0.25 mL dose of RTS,S/AS02A, but is formulated to supply a 0.5 mL dose. AS01B utilizes liposomes in place of proprietary oil-in-water emulsion. RTS,S/AS01E, the pediatric formulation of RTS,S/AS01B, is

composed of the same active constituents, but with half the quantities as in RTS,S/AS01B.

1.4. The RTS,S/AS02 candidate malaria vaccine; key clinical efficacy, safety and immunogenicity data

In the clinical trial setting to date the RTS,S/AS02A candidate vaccine has been administered to both malaria-naïve adult subjects (subjects who live in countries where there is no naturally occurring malaria transmission) and naturally exposed adults and children (subjects who live in countries where malaria transmission occurs naturally). The numbers of doses of RTS,S-containing vaccines and the number of recipients is tabulated in Table 2. A comprehensive summary of the results of reported trials to date can be found in the Malaria Vaccine Investigator Brochure [GSK data on file].

Table 2 Approximate number of doses of RTS,S/AS02A administered to date with number of recipients

Subject population	Recipients	Doses administered
Malaria-naïve adults [a]	247	596
Naturally-exposed adults [b]	266	842
Naturally-exposed children [b]	1292	3746

a subjects who took part in studies conducted in the USA and Belgium, where there is no naturally occurring transmission of malaria
b subjects who took part in studies conducted in malaria-endemic countries in Africa.

Early clinical development of the RTS,S malaria candidate vaccine was initiated in malaria-naïve adults in collaboration with the WRAIR in which confirmation of the efficacy, safety and immunogenicity of the RTS,S/AS02A vaccine formulation was demonstrated [Stoute 1997; Kester 2001]. Two doses of RTS,S/AS02A (0.5 mL) provided protection to 37.8% healthy non-immune volunteers against homologous sporozoite challenge (pooled results for WRMAL-004 [GSK data on file] & -005 [GSK data on file]); three doses demonstrated protection of 43.2% of subjects (pooled results for WRMAL-004 & Malaria-012 [GSK data on file]). In subjects not protected, the prepatent period (PPP) was significantly prolonged in the RTS,S/AS02A group compared to control (WRMAL-004, WRMAL-005 & Malaria-012). Protective efficacy was low following re-challenge six months after Dose 3 of RTS,S/AS02A, but a statistically significant difference between PPP for vaccinees compared to infectivity control was observed (WRMAL-003 [GSK data on file]). A strong humoral immune response to the RTS,S/AS02A vaccine in terms of anti-CS and anti-HBs antibodies was demonstrated in all the adult studies in malaria-naïve individuals. Evaluations of the CMI response showed consistently that administration of RTS,S/AS02A induced strong cellular Th1 T-cell responses, specific to the vaccine antigen [Lalvani 1999; Stoute 1998; Epstein 2004; Sun 2003].

The RTS,S/AS02A vaccine progressed to evaluation in subjects under conditions of natural transmission. In adult males from The Gambia, VE against infection adjusted for covariates was 71% (95% CI: 46 to 85; $p < 0.001$) during the first 2 months and 34%

(95% CI: 8.0 to 53, p=0.014) for the entire 15 week surveillance period (Malaria-005). VE, adjusted for covariates, following a booster dose given during a second year malaria season was 47% (95% CI: 3 to 71; p=0.039). Protection was not limited to the NF54 parasite genotype from which the vaccine was derived [Alloueche 2003]. Following review of SAEs and safety surveillance over approximately 5 years, no safety signal was apparent (Malaria-016, -017 & -018 [GSK data on file]). A strong humoral immune response to the RTS,S/AS02A vaccine in terms of anti-CS and anti-HBs antibodies was demonstrated. Overall, the kinetics of the humoral immune response induced by vaccination with RTS,S/AS02A were similar in malaria-naïve and experienced populations, while the absolute GMT values appeared to be higher in malaria-naïve volunteers. The vaccine induced and boosted Th1-like cellular immunity to several T-cell epitopes in a population naturally exposed to malaria.

The RTS,S/AS02A candidate vaccine progressed to clinical evaluation in children. Two age de-escalation and dose comparison trials (which compared doses with 0.1µg, 0.25µg and 0.5µg of antigen) enrolled a total of 225 children aged 1 to 11 years from The Gambia in which a similar pattern and intensity of reactogenicity following vaccination with RTS,S/AS02A to that of previous studies in semi-immune adults was demonstrated (Malaria-015 [GSK data on file] & -020 [GSK data on file]; [Bojang 2005]). RTS,S/AS02A was highly immunogenic for both anti-CS and anti-HBs antibodies, irrespective of pre-vaccination HBs serostatus. From these trials, the 0.25 mL dose was selected due to equivalent immunogenicity and slightly less reactogenicity in the 0.25 mL group compared to the 0.5 mL group; immunogenicity was consistently lowest in the 0.1 mL group. The safety and immunogenicity of the RTS,S/AS02A 0.25 mL dose was further confirmed in another study, Malaria-025 [GSK data on file], conducted in 1 to 4 year old children in Mozambique.

Subsequently, a large safety, immunogenicity and efficacy trial in children aged 1 to 4 years from an area of high transmission was conducted in Mozambique, enrolling a total of 2022 subjects (Malaria-026 [GSK data on file]; [Alonso 2004]; [Alonso 2005]). In this study, 3 doses of RTS,S/AS02A (0.25 mL dose) were administered according to a 0, 1, 2-month schedule to approximately 1000 children. The primary endpoint of this trial investigated vaccine efficacy against the onset of clinical malaria disease. Up to six months post Dose 3, the VE determined as the time to the first clinical episode (after adjustment for covariates) was 29.9% (95% CI: 11.0 to 44.8; p=0.004). For the entire study period, up to 18 months post Dose 3, VE for the same endpoint was 32.8% (95% CI: 20.1 to 43.4; p=0.0001).

The trial also offered the opportunity to investigate VE against multiple attacks of malaria and severe malaria. Against multiple attacks, VE was 27.4% (95% CI: 6.2 to 43.8; p=0.014) up to six months post Dose 3. For the entire study period, VE was 32.4% (95% CI: 17.6 to 44.5; p=0.0001). Against severe malaria, VE was 57.7% (95% CI: 16.2 to 80.6; p=0.019) over six months post Dose 3: for the entire study period VE was 48.6% (95% CI 12.3 to 71.0; p=0.02)

The proportion of children experiencing a SAE was similar in the RTS,S/AS02A and control groups; no SAE was judged to be related to vaccination. No difference in the pattern of morbidity notified as SAEs was observed between recipients of RTS,S/AS02A

and control vaccines; the pattern of morbidity was similar to that previously observed at the study site and described in the region. At least 97% of recipients of RTS,S/AS02A were seropositive for anti-CS antibodies and at least 97% of recipients of RTS,S/AS02A were seroprotected for anti-HBs antibodies 18 months post Dose 3 [Alonso, 2005].

In parallel, a 0.5 mL variant of the 0.25 mL dose of RTS,S/AS02A (RTS,S/AS02D) was developed for compatibility with standard auto-disable EPI syringe. RTS,S/AS02D, was shown in children aged 3 to 5 years in a malaria-endemic region of Mozambique to be safe and exhibit non-inferior immunogenicity to RTS,S/AS02A (0.25 mL dose) (Malaria-034); no SAE was reported in recipients of RTS,S/AS02D; non-inferiority of the anti-CS and anti-HBs antibody responses induced by the RTS,S/AS02D formulation as compared to the RTS,S/AS02A (0.25 mL dose) formulation was demonstrated.

1.5. The RTS,S/AS01B candidate malaria vaccine; preliminary clinical safety, efficacy and immunogenicity data

Recent preliminary results of a challenge study conducted in healthy malaria-naïve adults have been encouraging (Malaria-027 [GSK data on file]), indicating that RTS,S/AS01B may be more efficacious than RTS,S/AS02A. In this double-blind**,** randomized phase I/IIa human challenge study, the safety, reactogenicity, immunogenicity and preliminary efficacy after early sporozoite challenge and rechallenge, of RTS,S/AS01B and RTS,S/AS02A were assessed. Two sequential cohorts of approximately 52 subjects each were enrolled; each cohort was evenly divided into 2 groups receiving either the RTS,S/AS01B or RTS,S/AS02A vaccine (up to 48 infectivity controls for challenge and re-challenge phases were additionally enrolled). Subjects were vaccinated at 0, 1, 2 months followed by a challenge 14-30 days after Dose 3. Protected individuals were invited to be rechallenged approximately 6 months after Dose 3 to evaluate persistence of efficacy. To date, preliminary results for Cohort 1 are available.

Following primary challenge, the point efficacy of VE was significantly greater in both vaccine groups compared to infectivity control. Although not statistically significant, VE was higher in the RTS,S/AS01B group compared to RTS,S/AS02A (58.8% [95% CI: 32.1, 81.6] vs 37.5% [95% CI: 11.8, 59.4], p=0.2085). In non-protected subjects, the mean time to infection (pre-patent period; PPP) following challenge was similar in both vaccine groups and longer than in the infectivity control.

Following re-challenge, 2/5 subjects (40.0%) in the RTS,S/AS01B group and 4/5 subjects (80.0%) in the RTS,S/AS02A group were infected; all infectivity control subjects became infected. VE was significantly greater in the RTS,S/AS01B group compared to infectivity control (p=0.061) and greater compared to RTS,S/AS02A (60.0% [95% CI: 3.2, 95.1] vs 20.0% [95% CI: -60.2, 71.6], respectively). The PPP in non-protected subjects was longer in the RTS,S/AS01B group compared RTS,S/AS02A; PPP was longer in both vaccine groups compared to control.

The reactogenicity and safety profile of RTS,S/AS01B was comparable to RTS,S/AS02A. Both vaccines were shown to be safe, immunogenic and efficacious; two SAEs were reported (tendon rupture, cerebral infarction), both in the RTS,S/AS01B group, neither of which was considered to be related to study vaccine; anti-CS antibody

responses were greater in recipients of RTS,S/AS01B than of RTS,S/AS02A; anti-CS CD4+ T-cell responses were stronger in the RTS,S/AS01B group compared to RTS,S/AS02A; there was a trend towards improved efficacy with RTS,S/AS01B compared to RTS,S/AS02A.

1.6. Rationale for the study design

RTS,S/AS01 has been developed as a potential improvement to the RTS,S/AS02 vaccines. Preliminary results from the Malaria-027 trial indicate that the RTS,S/AS01B formulation may be more efficacious than RTS,S/AS02A while retaining its acceptable reactogenicity profile (refer to Section 1.5). RTS,S/AS01E is a pediatric formulation of RTS,S/AS01B, designed to be compatible with the requirement that all vaccines administered under the EPI of WHO have a dose volume of 0.5 mL.

This trial is designed to assess the safety of RTS,S/AS01E and demonstrate non-inferiority of the anti-CS immune response to RTS,S/AS02D in children aged 18 months to 4 years. This approach is implemented to ensure safety and immunogenicity of the new formulation before proceeding to trials in younger children and infants.

There are two reasons why the RTS,S/AS01E candidate malaria and hepatitis B vaccine is being developed for delivery through the infant Expanded Program on Immunization (EPI) of the World Health Organization (WHO). Firstly, the vaccine is being developed to prevent severe malaria disease which occurs from about 4 months of age coinciding with the time that maternally-acquired immunity wanes [Snow 1990; Snow 1994]. Secondly the EPI has been highly successful at increasing the coverage of basic vaccines across the developing world. With the goal of integration into the EPI infant regimen, a 0, 1, 2 month schedule will be evaluated in children and the final dose volume is adjusted to 0.5 mL to be compatible with the standard syringes used by EPI.

1.6.1. Rationale for CMI and B-cell memory testing

Neutralizing-antibody and CMI responses are thought to be essential immune effector mechanisms for protecting people vaccinated with RTS,S/AS02 and RTS,S/AS01. It is hypothesized that these responses act to limit hepatocyte invasion, destroy infected hepatocytes and/or limit intracellular parasite development.

RTS,S/AS02A has been shown to be a powerful inducer of antigen-specific humoral and CMI responses in preclinical and clinical studies [Malaria Investigator's Brochure 2005 GSK data on file]. Recent preliminary results of a challenge study conducted in healthy malaria-naïve adults (Malaria-027 [GSK data on file]) shows that RTS,S/AS01B induces more potent CMI responses characterized predominantly by CD4+ type 1 responses and similar antibody levels as compared to RTS,S/AS02A. This may be associated with increased vaccine efficacy on the part of RTS,S/AS01B; immunological monitoring performed during this trial revealed an association between protection against *P. falciparum* and CS-specific humoral and cellular response (Dr Kent Kester, WRAIR, personal communication, November 2005; publication in preparation).

CMI and B-cell memory immune responses induced by a vaccine antigen formulated in AS01 adjuvant have never been measured in children. We will compare CMI responses in children vaccinated with RTS,S/AS01E and RTS,S/AS02D, as this may reflect differences in vaccine efficacy. Responses will be measured on cells taken prior to vaccination, one month post Dose 2, one month post Dose 3, and 12 months post Dose 3. Antigen-specific B-cell memory immune response will also be characterized, as this may provide useful data regarding the respective ability of both adjuvants to induce long-term humoral immunity.

In order to limit the blood volumes collected from children, CMI and B-cell memory immune responses will not be determined in the same children. Half of the children will have CMI assessed, and the other half will have B-cell memory immune response characterized. Details of the assay methodology can be found in Appendix F. Attribution will be determined by a randomization list and balanced by vaccine assignment.

2. OBJECTIVES

2.1. Coprimary objectives

- For RTS,S/AS01E when administered as 3 doses intramuscularly on a 0, 1, 2-month schedule to children aged 18 months to 4 years living in a malaria-endemic area:
  - to assess safety until one month post Dose 3
  - to demonstrate non-inferiority to RTS,S/AS02D in terms of anti-CS antibody response one month post Dose 3.

Refer to Section 10.1 for definition of the coprimary endpoints.

2.2. Secondary objectives

- For RTS,S/AS01E when administered as 3 doses intramuscularly on a 0, 1, 2-month schedule to children aged 18 months to 4 years living in a malaria-endemic area:
  - to assess reactogenicity until one month post Dose 3
  - to demonstrate non-inferiority to RTS,S/AS02D in terms of anti-HBs antibody response one month post Dose 3
  - to describe seroprotection against hepatitis B up to one month post Dose 3
  - to describe the anti-CS response up to one month post Dose 3.

Refer to Section 10.2 for definitions of secondary endpoints.

2.3. Tertiary objectives

- To assess, for RTS,S/AS01E when administered as 3 doses intramuscularly on a 0, 1, 2-month schedule to children aged 18 months to 4 years living in a malaria-endemic area:
  - safety between 1 month post Dose 3 until 12 months post Dose 3.
  - humoral immune response to CS antigen at 12 months post Dose 3
  - humoral immune response to HBs antigen at 12 months post Dose 3.

Refer to Section 10.3 for definitions of tertiary endpoints.

2.4. Exploratory Objectives

- For RTS,S/AS01E and RTS,S/AS02D when administered as 3 doses intramuscularly on a 0, 1, 2-month schedule to children aged 18 months to 4 years living in a malaria-endemic area:
  - to evaluate T-cell-mediated immune response (CMI) to CS antigen up to 12 months post Dose 3
  - to evaluate B-cell memory immune response to CS antigen up to 12 months post Dose 3.

Refer to Section 10.4 for definitions of exploratory endpoints.

3. STUDY DESIGN OVERVIEW

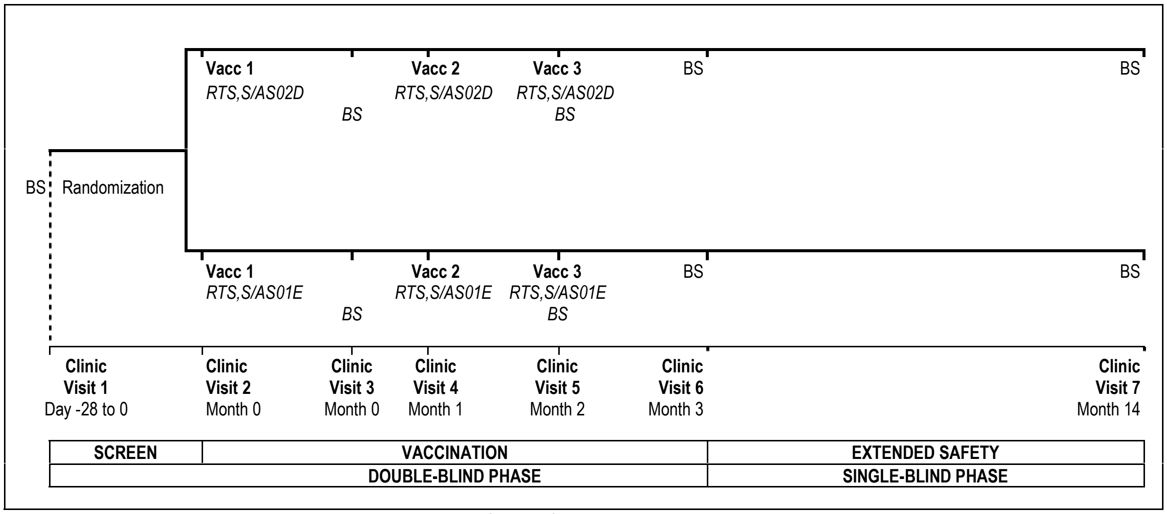

KEY: BS; Blood Sample. **Vacc;** Vaccination

- Experimental design: Phase II, single center, double-blind (observer blind, participant blind), randomized (1:1 ratio) trial with two groups in one study site.
- Healthy male and female children aged 18 months to 4 years of age will be screened; those determined to be eligible, based on the inclusion and exclusion criteria, will be enrolled in the study.

- Route of administration: all vaccines will be administered by the intramuscular route to the left deltoid.
- Each child will be observed for at least 60 minutes after vaccination to evaluate and treat any acute adverse events (AEs).
- This study will not capture cases of malaria for the analysis of efficacy. All cases of malaria presenting during the unsolicited period will be captured as unsolicited events. Cases at anytime during the study that meet the criteria for a SAE will be reported as SAEs.
- The access of the study population to HIV voluntary counseling and testing and HIV antiretroviral therapy according to national recommendations will be ensured, in collaboration with the government services.
- An insecticide impregnated bednet will be offered to all parent(s)/guardian(s) who present their child for screening.
- Blood for safety monitoring of hematology, renal and hepatic function will be measured at screening, one week post Dose 1, one month post Dose 3 and 12 months post Dose 3.
- Anti-CS antibody titers will be determined at screening, one month post Dose 2, one month post Dose 3 and 12 months post Dose 3.
- Anti-HBs antibody titers will be determined at screening, one month post Dose 2, one month post Dose 3 and 12 months post Dose 3.
- CMI and B-cell memory immune responses will be determined at screening, one month post Dose 2, one month post Dose 3 and 12 months post Dose 3.
- Timing of final analysis: 1 month post Dose 3. An annex safety analysis will be performed at the end of the study, one year post Dose 3.
- Data collection: conventional Case Report Form (CRF).

4. STUDY COHORT

4.1. The Albert Schweitzer Hospital Medical Research Unit

This study will be conducted at the Medical Research Unit (MRU) of the Albert Schweitzer Hospital (HAS – Hôpital Albert Schweitzer) in Lambaréné, Gabon. The HAS was founded in 1913. It is run by the International Foundation of the HAS in collaboration with the Ministry of Health of Gabon.

The MRU of the HAS was established in 1981. The scientific focus is on malaria research, and main areas of interest are parasite biology, pathophysiology, pathogen-host interaction, host immune response, clinical aspects and chemotherapy of malaria. Other areas of research comprise schistosomiasis, filariases and mechanisms of allergy development.

There are three research groups within the MRU: Clinical Trials, Molecular Epidemiology and Immunology. Currently the groups are staffed by approximately 30

temporary and permanent staff affiliated to an academic institution. Among these are several post-doctoral scientists as well as PhD and MD students.

The Clinical Trials group has a staff of four medical doctors, all of whom have had additional training in specific aspects of clinical trials (basic and advanced courses on clinical research, publication workshops, biostatistics and epidemiology). Technical staff comprises three technicians, who are responsible for routine laboratory diagnostics of pediatric out-patients and study subjects.

Annually, around ten students do research for their thesis in medicine at the unit. Such students come from both the University of Libreville and European universities.

The laboratory of the unit is well-equipped with a photometer, a cytometer, sterile workbenches and -80°C freezers. Implementation of a quality-control system is currently being planned. Hematological, biochemical and serological testing can all be carried out at HAS.

The unit has good information technology capability. Internet connectivity is provided via a satellite link with the MIMCOM network (Multilateral Initiative on Malaria Communications). An information technology manager is responsible for database management and the computer network. Several unit staff have a background in epidemiology and basic statistical analysis. When necessary, more sophisticated analytical support has been provided by the Department of Parasitology or the Department of Biometry, University of Tübingen, Germany.

The MRU receives no annual core budget, financing itself entirely through research projects and grant money. In the past the MRU has received funding from the European Union, the Medical Faculty of the University of Tübingen, the German Ministry of Research, the US National Institute of Health, WHO/TDR (Special Programme for Research and Training in Tropical Diseases), and the pharmaceutical industry. Currently a large proportion of the funding comes from the German MIM initiative as well as some other German research programmes.

The MRU has conducted many clinical trials to ICH/GCP standard. The internal review board, the Ethics Committee of the International Foundation of the HAS is registered with the US Department of Health and Human Services.

4.2. Geography and study population

The HAS is situated in Lambaréné, a town of approximately 20 000 inhabitants. Lambaréné is located in the Moyen Ogooué province of Gabon near the equator in the central African rain forest. There is little variation in temperature and humidity throughout the year, however there is substantially less rainfall in July and August.

The population consists of several Bantu groups, mainly belonging to the Fang, Eshira and Myéné group. Intermarriage between members of different tribes is common. Local languages are mainly spoken on the family level but have little importance in official life, where French is used. According to United Nations Children's Fund, the adult literacy

rate in 2000 was 71% (for males 80%, for females 62%). Timber and palm oil industries exist in town. Fishery and hunting is performed on a smaller scale but is of major importance. People in the study area live in clusters of houses close to roads with easy access to taxis and with telecommunications services.

4.3. Malaria epidemiology and control in the study area

Malaria transmission in the study area is intense and there is little seasonal variability in transmission rates or parasite prevalence [Wildling 1995; Sylla 2001]. *Plasmodium falciparum* is the predominant species and responsible for 95% of all infections. The main vectors are *Anopheles gambiae* and *An. moucheti*. The entomological inoculation rate lies around 50 infective bites per person per year [Sylla 2000].

The level of chloroquine resistance is high, reaching 100% both in vitro and in vivo [Borrmann 2002]. Resistance of local parasite strains to sulfadoxine/pyrimethamine is moderate and rising steadily. A recent study in Lambaréné found a treatment failure of 21% in children on Day 14 [Alloueche 2004]. The policy of the government of Gabon is to treat all children with malaria with Artemisinin-based Combination Therapy (ACT).

On average, children aged 2 to12 years experience about 1.5 malarial attacks per year, with large variability among individuals [Lell 1999]. Anemia is common: around 70% of children tested at HAS MRU have Hb < 9g/dL; about 17% of children have Hb < 5g/dL [Lell unpublished].

There is no widely implemented national or local bednet programme. Although part of the population sleep under bednets, these are rarely impregnated, installed properly or well-maintained.

4.4. Healthcare in the study area

Primary healthcare in the study area is available through dispensaries throughout Lambaréné. These offer vaccination services, mother and child healthcare and midwifery services (including delivery). In addition to the HAS, there is a government-run district hospital in the center of Lambaréné offering district level care. No consultation fees are levied at the District General Hospital, but patients are charged for each procedure carried out.

HAS is located on the outskirts of Lambaréné town but well integrated into the care of the community. There are approximately 200 staff at the HAS, headed by 8 physicians. In total, approximately 200 beds are available to departments of internal medicine, surgery, pediatrics and obstetrics. In addition there is a dental clinic, a community health service where mother and child vaccinations are administered, and the MRU. The pediatric clinical service of HAS is led by two specialist pediatricians. The pediatric ward can offer a number of diagnostic and clinical services that are unavailable elsewhere in Lambaréné. Medical care is available at HAS 24 hours a day.

The pediatric ward has the ability to provide district level clinical care. Emergency services are also available. There are basic blood bank services; donations are screened

for infection with HIV, hepatitis B virus, hepatitis C virus, loa loa and syphilis. X-ray and ultrasound facilities are available. Cases requiring more extreme intensive care, as well as cases of renal insufficiency, endocrine abnormalities or suspected malignancies can be transferred by ambulance the Centre Hospitalier de Libreville, a 3 to 4 hours drive. A consultation fee is levied by HAS, but this fee is inclusive of all charges for procedures.

4.4.1. HIV services

The HIV seroprevalence is an estimated 4% [Debat Zoguereh 2004]. At HAS, HIV testing is performed using Determine® (Abbott Laboratories, Illinois, USA). A positive or inconclusive test is validated using ImmunoComb® (Orgenics, Yavne, Israel). The HAS is one of the centers of the national programme for HAART (highly active antiretroviral therapy) distribution. Voluntary counseling and CD4 count testing are available. Treatment for the patient is provided free of charge.

4.5. Number of subjects

Children will be recruited by non-coercive methods according to existing policies of the study site (see Appendix C).

The necessary number of children will be screened in order to enroll approximately 180 eligible subjects, 90 to each group. See Section 10.5 for a detailed description of the criteria used in the estimation of sample size.

4.6. Inclusion criteria for enrolment

All subjects must satisfy the following criteria at study entry:

- A male or female child between 18 months and 4 years of age (up to but not including 5th birthday) at the time of first vaccination.
- Written or oral, signed or thumb-printed and witnessed informed consent obtained from the parent(s)/guardian(s) of the child.
- Subjects who the investigator believes that their parents/guardians can and will comply with the requirements of the protocol (e.g. return for follow-up visits) should be enrolled in the study.

4.7. Exclusion criteria for enrolment

The following criteria should be checked at the time of study entry. If any apply, the subject must not be included in the study:

- Acute disease at the time of enrolment (acute disease is defined as the presence of a moderate or severe illness with or without fever). All vaccines can be administered to persons with a minor illness, such as diarrhea or mild upper respiratory infection without fever, i.e. axillary temperature < 37.5°C.

- Serious acute or chronic illness determined by clinical or physical examination and laboratory screening tests including, but not limited to:
  - Any confirmed or suspected immunosuppressive or immunodeficient condition, based on medical history and physical examination (no laboratory testing required)
  - A family history of congenital or hereditary immunodeficiency
  - History of splenectomy
  - Major congenital defects
  - History of any neurologic disorders or seizures
  - Moderate malnutrition at screening defined as weight for age Z-score less than -2.
- Laboratory screening tests out of range, specifically:
  - refer to Table 10
- Planned administration/administration of a vaccine not foreseen by the study protocol within 30 days of the first dose of vaccine(s) with the exception of tetanus toxoid.
- Use of any investigational or non-registered drug or vaccine within 30 days preceding the first dose of study vaccine, or planned use during the study period.
- Administration of immunoglobulins, blood transfusions or other blood products within the three months preceding the first dose of study vaccine or planned administration during the study period.
- Chronic administration (defined as more than 14 days) of immunosuppressants or other immune-modifying drugs within six months prior to the first vaccine dose (for corticosteroids, this will mean prednisone, or equivalent, $\geq$ 0.5 mg/kg/day. Inhaled and topical steroids are allowed).
- Previous participation in any other malaria vaccine trial.
- Simultaneous participation in any other clinical trial.
- Same sex twin.
- History of allergic reactions (significant IgE-mediated events) or anaphylaxis to previous immunizations.
- History of allergic disease or reactions likely to be exacerbated by any component of the vaccine.
- Any other findings that the investigator feels would increase the risk of having an adverse outcome from participation in the trial.

4.8. Elimination criteria during the study

The following criteria should be checked at each visit subsequent to the first visit. If any become applicable during the study, it will not require withdrawal of the subject from the

study but may determine a subject's evaluability in the according-to-protocol (ATP) analysis.

- Administration of a vaccine (except tetanus toxoid) not foreseen by the study protocol during the period starting from 30 days before Dose 1 and ending 30 days after Dose 3.
- Use of any investigational or non-registered product (drug or vaccine) other than the study vaccines during the study period.
- Administration of immunoglobulins and/or any blood products during the study period.
- Chronic administration (defined as more than 14 days) of immunosuppressants or other immune-modifying drugs during the study period (for corticosteroids, this will mean prednisone, or equivalent, ≥ 0.5 mg/kg/day. Inhaled and topical steroids are allowed).

4.9. Contraindications to subsequent vaccination

4.9.1. Indications for deferral of vaccination

The following AEs constitute contraindications to administration of RTS,S/AS02D or RTS,S/AS01E at that point in time; if any one of these AEs occur at the time scheduled for vaccination, the subject may be vaccinated at a later date, within the time window specified in the protocol, or withdrawn at the discretion of the investigator. AEs should be followed-up according to the instructions in Section 8.6:

- Acute disease at the time of administration of investigational product (acute disease is defined as the presence of a moderate or severe illness with or without fever). All vaccines can be administered to persons with a minor illness such as diarrhea or mild upper respiratory infection without fever, i.e. axillary temperature < 37.5°C.
- Axillary temperature of ≥ 37.5°C.

4.9.2. Absolute contraindications to further vaccination

The following AEs constitute absolute contraindications to further administration of RTS,S/AS02D or RTS,S/AS01E; if any of these AEs occur during the study, the subject must not receive additional doses of vaccine, but may continue other study procedures at the discretion of the investigator. AEs should be followed-up according to the instructions in Section 8.6:

- Acute allergic reaction (significant IgE-mediated events) or anaphylaxis following the administration of vaccine investigational product.
- Any confirmed or suspected immunosuppressive or immunodeficient condition, including human immunodeficiency virus (HIV) infection.
- Failure to thrive.

5. CONDUCT OF STUDY

5.1. Ethics and regulatory considerations

The study will be conducted according to Good Clinical Practice (GCP), the Declaration of Helsinki (Protocol Appendix A), and local rules and regulations of the country.

Submission of the protocol and any protocol amendments to regulatory agencies will occur in accordance with local regulatory requirements. The timing of the submission relative to IEC/IRB submission or approval and whether or not the authority will provide their approval of or favorable opinion on the protocol or amendment before it can be implemented will depend on local regulatory requirements.

5.1.1. Institutional Review Board/Independent Ethics Committee (IRB/IEC)

Each IRB/IEC will be constituted according to the local laws/customs of each participating country. The ICH Harmonized Tripartite Guideline for Good Clinical Practice recommends that IRBs/IECs should include:

a. At least five members.

b. At least one member whose primary area of interest is in a non-scientific area.

c. At least one member who is independent of the institution/ study site.

Only those IRB/IEC members who are independent of the investigator and the sponsor of the study will vote/ provide opinion on a study-related matter.

A list of the professions of the IRBs'/IECs' members will be obtained by the PI or delegate.

This protocol and any other documents that the IRBs/IECs may need to fulfill their responsibilities, including subject recruitment procedures and information about payments and compensation available to subjects, will be submitted to each IRB/IEC by the PI or delegate. Written and dated unconditional approval/favorable opinion from each IRB/IEC of the protocol and amendment (if any and applicable), written informed consent form ICF), consent form updates (if any), subject recruitment procedure(s) (e.g. advertisements), and any other written information to be provided to subjects will be in the possession of the investigator and GSK before commencement of the study. This approval/favorable opinion will refer to the study by study title and number with exact protocol version and date, and will identify the documents reviewed and state the date of review. Relevant GSK Biologicals' data will be supplied by the GSK Biologicals' Central Study Coordinator to the independent IRBs/IECs for review and approval of the protocol. Verification of IRBs/IECs unconditional approval of the protocol and the written informed consent statement will be transmitted by the PI to the GSK Biologicals' Central Study Coordinator, using the standard notification form, prior to shipment of vaccine supplies and CRFs to the site.

No deviations from, or changes to, the protocol will be initiated without prior written sponsor and IRBs'/IECs' approval/ favorable opinion of an appropriate amendment, except when necessary to eliminate immediate hazards to the subjects or where permitted by all applicable regulatory requirements or when the change(s) involves only logistical or administrative aspects of the study (e.g., change of monitor[s], telephone number[s].) Administrative changes and amendments not submitted for approval will be submitted to the IRB/IEC for information only. However, written verification that such documents were submitted will be obtained. Approvals/ verifications will be transmitted in writing by the PI.

The IRB/IEC will be informed by the GSK Biologicals' Central Study Coordinator of:

- all subsequent protocol amendments, informed consent changes or revisions of other documents originally submitted for review,
- serious and/or unexpected adverse events occurring during the study,
- all subsequent protocol administrative changes (for information),
- new information that may affect adversely the safety of the subjects or the conduct of the study,
- regular updates and/or request for re-approval,
- when the study has been completed.

If the trial is prematurely terminated or suspended for reasons including, but not limited to, safety or ethical issues or severe non-compliance, the sponsor will promptly inform the regulatory authorities of the termination or suspension and the reason(s) for the termination or suspension. If required by applicable regulations, the investigator will inform the IEC/IRB promptly and provide the reason for the suspension or termination (see Appendix B for further details).

5.1.2. Informed consent

The details of the informed consent process are provided in Appendix C. The following principles will also apply.

In obtaining and documenting informed consent, the investigator will comply with the applicable regulatory requirement(s), and will adhere to GCP and to the ethical principles that have their origin in the appended Declaration of Helsinki. Prior to the beginning of the trial, the investigator will have the IRBs'/IECs' written approval/favorable opinion of the written informed consent form and any other written information to be provided to the subjects' parents/guardians.

Information will be given in both oral and written form whenever possible and as deemed appropriate by the IRBs/IECs.

An investigator or designate will describe the protocol to potential subjects' parents/guardians face to face. The ICF may be read to the subjects' parents/guardians, but, in any event, the investigator or designate shall give the subjects' parents/guardians

ample opportunity to inquire about details of the study and ask any questions before dating and signing the ICF.

ICFs will be in a language fully comprehensible to the prospective subjects' parents/guardians. Informed consent shall be documented by the use of a written ICF approved by the IRBs/IECs and signed/thumbprinted and dated by the subjects' parents/guardians and by the person who conducted the informed consent discussion. The parent(s)/guardian(s) signature/thumbprint confirms the consent is based on information that has been understood. All individuals will have the study and the ICF explained to them point by point by the interviewer. Where parent(s)/guardian(s) are illiterate, an impartial witness will be present. The subjects' parents/guardians will sign (or thumbprint, where illiterate) the consent form. When parent(s)/guardian(s) are illiterate, the witness will also sign and date the consent form.

Each subject's signed informed consent form will be kept on file by the investigator for possible inspection by Regulatory Authorities and/or GSK Biologicals' Professional and Regulatory Compliance persons. The subjects parent(s)/guardian(s) will receive a copy of the signed and dated written informed consent form and any other written information provided to the parent(s)/guardian(s) and will receive copies of any signed and dated consent form updates. Any amendments to the written information will be provided to subjects parent(s)/guardian(s).

Both the informed consent discussion and the written informed consent form and any other written information to be provided to the subjects' parent(s)/guardian(s) will include explanations of the following:

a. That the trial involves research.

b. The purpose of the trial.

c. The trial vaccine regimens and the probability for random assignment to each vaccination regimen.

d. The trial procedures to be followed, including all invasive procedures.

e. The subject's parents'/guardians' responsibilities.

f. Those aspects of the trial that are experimental.

g. The reasonably foreseeable risks or inconveniences to the subjects.

h. The reasonable expected benefits.

i. The alternative procedure(s) or course(s) of treatment/ methods of prevention that are available to subjects, and their important potential benefits and risks.

j. The compensation and/or treatment available to subjects in the event of trial-related injury.

k. The anticipated expenses to subjects' parents/guardians for participating in the trial.

l. That the subjects' participation in the trial is voluntary and subjects' parents/guardians may refuse to participate or withdraw from the trial, at any time, without penalty or loss of benefits to which subjects are otherwise entitled.

m. That the monitor(s), the auditor(s), the IRBs/IECs, and the regulatory authority(ies) will be granted direct access to the subject's original medical records for verification of clinical trial procedures and/or data, without violating the confidentiality of subjects, to the extent permitted by the applicable laws and regulations and that, by signing a written informed consent, the subject's parents/guardians are authorizing such access.

n. That records identifying subjects will be kept confidential and, to the extent permitted by the applicable laws and/or regulations, will not be made publicly available. If the results of the trial are published, subjects' identity will remain confidential.

o. That the subjects' parents/guardians will be informed in a timely manner if information becomes available that may be relevant to the subjects' parents/guardians willingness for continued participation in the trial.

p. The person(s) to contact for further information regarding the trial and the rights of trial subjects, and who to contact in the event of trial-related injury.

q. The foreseeable circumstances and/or reasons under which a subject's participation in the trial may be terminated.

r. The expected duration of a subject's participation in the trial.

s. The approximate number of subjects involved in the trial.

GSK Biologicals will prepare a model ICF which will embody all the elements described above. While it is strongly recommended that this model document be followed as closely as possible, the informed consent requirements given in this document are not intended to pre-empt any local regulations which require additional information to be disclosed for informed consent to be legally effective. Clinical judgment, local regulations and requirements will guide the final structure and content of the document.

The investigator has the final responsibility for the final presentation of ICF, respecting the mandatory requirements of local regulations. The ICF generated by the investigator with the assistance of the sponsor's representative, will be approved (along with the protocol, and any other necessary documentation) by the IRBs/IECs and be acceptable to GSK Biologicals.

5.1.3. Safety monitoring plan

This trial is overseen by a Data Safety Monitoring Board (DSMB), operating under a charter, assisted by a Local Safety Monitor (LSM).

The DSMB will be notified of all SAEs within 24 hours. In addition there are defined points during the trial at which cumulative safety data will be reviewed by the LSM and the DSMB. There will be two reports as defined in Section 5.1.3.3.

The PI, LSM and DSMB are empowered to suspend the trial for any safety concern. To supplement this, the protocol defines criteria for the suspension of vaccination (refer to Section 5.1.3.4).

5.1.3.1. Data Safety Monitoring Board (DSMB)

An independent committee consisting of experts in malaria, pediatrics, statistics and other appropriate disciplines has been appointed to oversee ethical and safety aspects of the study conduct. A quorum of 3 members is required at scheduled meetings.

The role of the DSMB includes the review of the implementation and progress of the study. It provides initial, regular, and closing advice on safety-related issues to GSK Biologicals. Its advice is based on the interpretation of study data with reference to the study protocol.

The DSMB will confer before the initiation of the study (pre-initiation review), during the study at points of safety review and at the close of the study. They will review the Protocol and Report and Analysis Plan (RAP). Other unscheduled meetings may be required. Meetings must be documented and minutes made available to the sponsors. The DSMB may, if deemed necessary, convene a meeting with, or request further information from the Principal Investigators, the Medical Monitor/Local Safety Monitor and GSK Biologicals' and MVI at PATH's designated project representatives at any stage of the study.

The DSMB is empowered to suspend the enrollment to the trial and/or vaccination on the trial pending review of potential safety issues; complete details of this process are given in Section 5.1.3.4.

5.1.3.1.1. Data Reviewed by the DSMB

The DSMB must be informed by the Local Safety Monitor (LSM) of the following safety data on an 'as received' basis:

- All SAEs;
- All withdrawals of study subjects by the Principal Investigator or the parent(s)/guardian(s) of a subject due to adverse events.

The DSMB will receive from the sponsor, GSK Biologicals:

- Safety summary reports at two predefined timepoints as defined in Section 5.1.3.3;
- New information that may affect adversely the safety of the subjects or the conduct of the study;
- All subsequent protocol amendments, ICF changes or revisions of other documents originally submitted for review;
- All subsequent protocol modifications (for information).

5.1.3.2. Local Safety Monitor (LSM)

The overall role of the Local Safety Monitor (LSM), who is an experienced clinician based in Lambaréné, will be to support the clinical investigators and to act as a link between the investigators and the DSMB.

The LSM's role will include:

- Acting as the study volunteer's advocate;
- Promptly communicating relevant safety information to the DSMB;
- Providing advice to the investigators on whether a set of clinical circumstances in a study warrants formal notification to the DSMB;
- Unblinding a subject if deemed necessary to allow for adequate treatment;
- Liaising closely with the chair of the DSMB throughout the course of the trial;
- Suspension of vaccination for a major safety concern pending discussion with the DSMB (see Section 5.1.3.4 for full details).

5.1.3.2.1. Data Reviewed by the LSM

The LSM must be informed by the investigator on an 'as received' basis of:

- All SAEs;
- All withdrawals of study subjects by the Principal Investigator or the parent(s)/ guardian(s) of a subject due to adverse events.

5.1.3.3. Safety monitoring reports

Safety reports will be produced for the DSMB on all data collected until two points in the study; after 40 subjects have been enrolled and have completed the first 7 days of follow-up post Dose 1 and after 40 subjects have completed the first 7 days of follow-up post Dose 2. An independent statistician will analyze the data and prepare an unblinded report for the DSMB thereby maintaining the blind of the malaria project team at GSK Biologicals and the investigator group. If a criterion for the suspension of progression to the next sequential vaccine dose is met, or the DSMB have any safety concerns about the vaccines, they may suspend the progression to the next vaccine dose. The process outlined in Section 5.1.3.4 will be followed.

The reports will contain for the first 40 children to receive each sequential vaccine dose:

- All SAEs and any relationship to vaccine (RTS,S/AS02D or RTS,S/AS01E) to date;
- For each of the first two doses (i.e. after Dose 1 and Dose 2) of RTS,S/AS02D and RTS,S/AS01E, all solicited AEs tabulated by severity grading (any and Grade 3 alone – refer to Table 9) and relationship to vaccine;
  - Should the investigator judge a case of Grade 3 fever to be unrelated to vaccination, an alternative explanation for the cause of the fever will be provided
- For each of the first two doses (i.e. after Dose 1 and Dose 2) of RTS,S/AS02D and RTS,S/AS01E, all unsolicited AEs tabulated by severity grading (any and Grade 3 alone– refer to Table 9) and relationship to vaccine;

- All laboratory values up to 30 days post Dose 2 presented as number of subjects out of range (above and below normal range) at each sampling time point (post doses 1 and 2 of RTS,S/AS02D or RTS,S/AS01E) tabulated by toxicity grading scale (refer to Table 10);
  - For all subjects with toxicity grading scale ≥ 3, full clinical details will be provided
- All withdrawals of study subjects by the Principal Investigator due to adverse events recorded from the children or withdrawals of children by the parent(s)/guardian(s) following doses of RTS,S/AS02D or RTS,S/AS01E (expressed as percentage of subjects enrolled).

5.1.3.4. Process for the suspension of progression to the next sequential vaccine dose

Suspension of the next sequential vaccine dose, pending full review of available data by the DSMB will take place if:

- The Principal Investigator suspends vaccination for any of the following SAEs pending review by the DSMB;
  - Death or life-threatening SAE which is judged to be related to the study vaccine;
  - Anaphylactic shock reaction in an enrolled subject following vaccination.
- The DSMB recommend suspension of all vaccination for any one SAE or pattern of SAEs. The DSMB will communicate their recommendation to the Principal Investigator who will enact it. The DSMB will notify the sponsors of their decision immediately;
- A safety report shows > 5% of subjects vaccinated with RTS,S/AS01E, or > 5% of subjects vaccinated with RTS,S/AS02D are withdrawn by the investigator for local or systemic reactogenicity in recipients;
  - In making their recommendation the DSMB will take into account the full clinical history of each withdrawn child.
- A safety report shows > 5% of doses of RTS,S/AS02D or > 5% of doses of RTS,S/AS01E are followed by fever > 39.0°C judged to be related to vaccination in recipients of RTS,S/AS01E;
  - In making their recommendation the DSMB will review the investigators assessment of relatedness of all Grade 3 fevers (refer to Table 9).
- The DSMB recommend suspension of enrollment following their review of a safety summary reports.

5.1.3.5. Process if the trial is suspended on the basis of safety reports on first 40 subjects to receive each of the first two vaccine doses

If the trial is suspended, the DSMB will review all available information (which will include the experience of all children to have been vaccinated) and make a recommendation to the study sponsor (GSK Biologicals) whether to recommence the trial

and proceed to the next sequential vaccine dose, or to stop the trial permanently. In the event that the DSMB recommend to stop the trial permanently, the FDA will be informed by GSK Biologicals that the trial is suspended.

Although the trial may be suspended by the DSMB, the LSM or the Principal Investigator, it is the responsibility of the sponsor (GSK Biologicals) to make the recommendation whether or not the trial should be stopped permanently.

In the event that the trial is suspended on the recommendation of the DSMB the sponsor (GSK Biologicals) will evaluate the information. If the sponsor concurs with the DSMB's recommendation to suspend the trial, GSK Biologicals will inform the FDA that the trial has been stopped permanently. If the sponsor's recommendation is to continue, then a report will be submitted to the FDA detailing the rationale used in reaching this decision. The agreement of the FDA will be obtained prior to restarting the trial.

5.2. Storage of study documentation at investigator's site

All study documentation containing personal information relating to study subjects will be kept in a secure locked area at the investigator's site. Such documentation will only be made available to authorized personnel. All electronic data kept at the investigator's site are kept secure. Computer access is only available to authorized personnel.

5.3. Recruitment/Screening

5.3.1. Community information

The community in which the study will take place will be informed about the nature and design of the study. Refer to Appendix C for an overview of the recruitment plan of the study.

5.3.2. Screening of volunteers

Only children with a written ICF, signed/thumb-printed and dated by parents/guardians will be screened.

Comprehension of the information contained within the ICF will be checked prior to screening by an oral interview with the volunteer's parent(s)/guardian(s). If consent is not available from both parents or guardians, the reason for the unavailability of one of the parents or guardians will be specified on the ICF form. When the parent(s)/guardian(s) of the child are illiterate, the ICF will also be signed and dated by a witness.

Subject numbers will be allocated to all volunteers who are consented for screening by their parent(s)/guardian(s). Subject numbers will be issued consecutively. Once consent is obtained, then per-protocol eligibility criteria will be checked, which will necessitate a physical examination and blood sampling for assessment of hematology, renal and liver function. This will be documented on clinic forms, which are prepared and filled in by the investigator. Each form will contain the subject number, information about the

volunteer's date of birth, household, date of screening visit, medical history, physical and laboratory screening examination. After reviewing the medical history, physical examination and laboratory results, any reasons for non-eligibility will be documented in the CRF.

The parent(s)/guardian(s) of children who have been consented for the study and found to be ineligible will receive a full explanation by a study clinician. Any clinically relevant finding will be treated appropriately by a physician. Where necessary the child will be referred to a specialist at Centre Hospitalier de Libreville for evaluation and treatment as described in Section 5.8. An overview of the laboratory assays can be found in Appendix D and further details can be found in HAS SOPs.

A study identification card will be prepared for the parent(s)/guardian(s) of each screened subject. At screening a photograph of each screened subject being held by their parent(s)/guardian(s) will be taken and attached to the study identification card. This card will also bear the name of the study to which the child is enrolled, and the child's subject number.

5.4. Vaccination process

The vaccines RTS,S/AS02D and RTS,S/AS01E will be packaged in identical boxes and will be identified by a treatment number. The unique treatment number will identify all doses of these vaccines administered to each subject. After randomization the treatment number assigns the subject to one group or another in a blinded way. Each subject will retain the same treatment number for their subsequent vaccine doses. The treatment number will be recorded on the subject's Clinic Form after the vaccine has been administered; information from the Clinic Form is subsequently transferred to the Case Report Form. The Clinic Form and Case Report Form link the subject number and the treatment number.

All vaccines will be administered by the intramuscular route to the left deltoid. Subjects who receive their vaccination in the incorrect arm will continue to receive subsequent blinded vaccinations as normal. The fact that the vaccine was administered in the wrong arm will be documented.

Vaccinations will take place at the HAS MRU or the outpatient clinic of HAS. All vaccinations will be given by a qualified person; a nurse or a doctor. A staff member experienced in the resuscitation of children will be available at all vaccination sessions. Facilities and equipment will be available to give emergency treatment in the case of an anaphylactic reaction following administration of vaccines. All children will be observed for an hour after the administration of vaccine to evaluate and treat any acute adverse events.

The process for each vaccination is as follows. The identity of the child will be confirmed using the study identification card. The subject number on this card will be cross-checked with that on the subject's clinic form, ensuring that the subject number on the clinic form matches that of the study identification card. On the day of the first vaccination the contraindications to vaccination, inclusion/exclusion criteria and ICF will be checked prior to vaccination. On the days of the subsequent vaccinations, elimination criteria and

contraindications to vaccination will be checked prior to vaccination. A vaccine clinic form will be initiated, which will give the child's identifiers, subject number and treatment number assigned after the administration of the vaccine.

Vaccines will be administered in a blinded fashion. For each vaccination during the course of the study, the Vaccine Preparer will prepare the vaccine and the Vaccinator administer the vaccine for a specific subject. Since the vaccines used in this study are of distinct appearance, the Vaccine Preparers are not blinded and together with the Vaccinators perform no other function in the study (refer to Section 6.5). The Vaccine Preparer will select the sealed box labeled with the subject's treatment number, containing the vials numbered with the treatment number, remove the vaccine vials and fill a syringe according to this study protocol (refer to Section 6.2). The Vaccine Preparer will then place a numbered opaque label with the subject's treatment number on the syringe. The purpose of masking the syringe is to blind the parent(s) or guardians(s) of the subject. The Vaccine Preparer will then pass the syringe to the Vaccinator in an adjacent room who will administer the vaccination. After administering the vaccination to the subject the Vaccinator will enter the treatment number administered to the subject on the clinic form.

Subjects who cannot be vaccinated on the originally scheduled date (see Section 4.9.1) will be vaccinated within 7 days and undergo all study procedures for the visit on the same day as vaccination. In the particular case of any child found to be febrile (axillary temperature ≥ 37.5°C), a blood slide will be taken to investigate for malaria. Children will be treated as appropriate for their condition and will be followed up until resolution of any symptoms and be vaccinated if their clinical symptoms resolve within 7 days.

Those who cannot be re-vaccinated within 7 days of their scheduled date will continue all study procedures apart from receiving further study vaccinations.

5.5. Home follow-up visits for assessment of reactogenicity (7-day follow-up period)

Trained field workers under the supervision of the Principal Investigator will visit each enrolled child at daily intervals for Days 1 to 5 post Dose 1; a study clinician will examine the child in the clinic on Day 6. For all other doses, the field workers will visit each enrolled child at daily intervals for Days 1 to 6 post each vaccination (see below in detailed study procedure; Section 5.11). In the event that the field worker finds any Grade 3 solicited general or unsolicited symptoms, the volunteer will be brought to HAS for examination by a study clinician. Any further clinical data, including treatment provided, will be written on diary cards and HAS clinic forms and transcribed onto the CRF. If the physician finds that the volunteer has experienced an SAE the appropriate measures will be taken to report this (See Section 8.7).

Diary cards will be checked and verified by the Principal Investigator or designate before transcription onto CRFs after the 7-day follow-up. The Principal Investigator has a primary responsibility for the data transcribed onto the CRFs. Unresolved AEs will be followed-up according to the instructions in Section 8.6 by field workers under the

supervision of the Principal Investigator and data will be entered onto the CRF. The procedures and frequency of visits will be outlined in a SOP at the investigator's site.

Analgesics/antipyretics will be provided to field workers for the treatment of children with injection site pain and fever and their use will be documented. Parent(s)/guardian(s) will not routinely be provided with these medications.

5.6. Monitoring of hematological and biochemical laboratory parameters

Blood for safety monitoring of hematology, renal and hepatic function will be measured at screening, one week post Dose 1, one month post Dose 3 and 12 months post Dose 3.The hematological and biochemical parameters will be documented on the CRF. For all values outside the normal range (refer to Table 10), the reason and/or clinical condition will be documented. Results of hematological and biochemical laboratory tests will be reviewed as soon as they are generated. Any value outside the normal range will be managed as appropriate by a medically qualified individual under the supervision of the Principal Investigator (refer to Section 8.6). Guidance on when to report abnormalities as SAEs is given in Section 8.3.

5.7. Surveillance for SAEs (all subjects)

5.7.1. At health facilities

Morbidity surveillance will be in place for this trial. The HAS will be the source of primary outpatient and inpatient care for the study participants. The surveillance system will provide a comprehensive recording of all outpatient attendances, the investigational results, diagnosis and management. Prior to the start of the study, all parent(s)/guardian(s) of volunteers will be educated on the appropriate action they should take if their child becomes unwell at any time during the study period. They will be asked not to medicate their child at home, but to seek medical care at the HAS.

A physician will be available 24 hours per day at HAS to receive and identify study participants when they present and to ensure complete investigation and documentation of the attendance.

All children attending as outpatients within the age range of the trial will be asked if the child is in this study and to provide the child's vaccine study identity card (see Section 5.3.2 for details of identity cards). If the identity cards are not available, the identity of the child will be confirmed against details collected at enrollment to the trial.

The child will be fully assessed by an appropriately qualified physician. This information will be recorded on a HAS clinic form. This form will provide a record of the child's name and study number, key symptoms and signs, axillary temperature, results of the laboratory tests and imaging examinations available at the time of form completion, the diagnosis, the treatment prescribed and will state whether hospital admission was required.

These forms will be retained by the study personnel in the HAS MRU Outpatient Department until they are sent to the HAS MRU data unit at the end of each working day. At the HAS MRU data unit, the questionnaires will be reviewed by the Principal Investigator or delegate. If it is necessary to report any of these consultations as a serious adverse event, the Principal Investigator or delegate will review the child as necessary and complete an SAE form to report the event (as specified in Section 8.5).

Children requiring inpatient care will be admitted to HAS, their clinical course, treatment and results of further investigations will be documented in their hospital records. The hospital records and inpatient surveillance will be the source documents for the SAE reports. When necessary, children will be followed to resolution of SAEs as outpatients.

5.7.2. In the community

Up to Clinic Visit 6 (approximately one month post Dose 3) volunteers will be seen at least monthly at clinic visits, and for 6 days following each dose. After Clinic Visit 6 (i.e. during the single-blind phase of the study) capture of SAEs will be enhanced by means of monthly visits by field workers and a final Clinic Visit at Month 14 (Clinic Visit 7).

During the field worker visits, the children's parent(s)/guardian(s) will be asked retrospectively if any SAEs occurred since the last visit and this information will be recorded. Unreported SAEs detected in this way will be investigated and reported by the Principal Investigator or delegate on the corresponding SAE forms (see Section 5.8). In the case of a death which has occurred at home, supplementary information will be gained using the verbal autopsy technique. The verbal autopsy will be conducted according to previously published methods and detailed in the SOPs on file with the investigators [Smith 1991].

If any child is reported to be unwell at the time of a visit, the field worker will advise the parent(s)/guardian(s) to seek care at HAS. In the event that a child is seriously ill, the field worker will inform the Principal Investigator or designate, and transport will be arranged to HAS, if judged appropriate by the responsible clinician.

5.8. Provision of health care

In order to maximize capture of adverse events parents/guardians will be encouraged to bring their children for all primary health care to the MRU of HAS. To facilitate this, the study will reimburse parents/guardians for the costs of bringing their children to the hospital. This strategy has been successfully implemented in trials carried out by HAS MRU.

Contact details for the PI will be provided to parent(s)/guardians(s) on the ICF. Medical attention is available on a 24-hour basis, seven days a week at HAS. Children requiring hospitalization will be admitted to the pediatric ward of the HAS. Care there will be provided collaboratively by MRU physicians and HAS pediatric clinical staff. The diagnostic and treatment facilities available at HAS are described in Section 4.4.

Children requiring specialized care or investigation unavailable at HAS such as treatment of complex renal insufficiency, endocrine abnormalities or malignancies will be transported Centre Hospitalier de Libreville. If this is considered necessary by the supervising clinician, HAS will facilitate the transfer of study subjects to Centre Hospitalier de Libreville.

If a volunteer is found to have a medical condition that excludes them from participation in the trial (whether diagnosed during screening or during the trial), their parent(s)/guardian(s) will be informed at a private appointment with a member of the clinical staff of the research team. The clinical staff member will take as much time as is required to explain the condition, including its severity, potential causes, long-term implications, impact on current and future lifestyle of both the child and the parent(s)/guardian(s) and evaluation and treatment options. Once it is clear that the parent(s)/guardian(s) understand the medical condition, the clinical staff member will develop an evaluation and treatment plan with the parent(s)/guardian(s) and ensure that the options are understood.

If the clinical staff member feels that the subject requires specialty or sub-specialty care, HAS will coordinate with the relevant physician and parent(s)/guardian(s) to make an appointment and provide transportation to the appointment if necessary. All history, physical examination and laboratory findings available will be provided to the parent(s)/guardian(s) and the referral physician.

Treatment for all medical conditions will be given according to the standard treatment regimens of Gabon. Any expenses — including transport — incurred by the parents/guardians of study participants for clinical care related to acute conditions will be borne by the study. Long-term care for chronic conditions unrelated to study procedures will be delivered following local guidelines with no financial support from the study.

A detailed description of the healthcare system available in the study area is provided in Section 4.4.

5.8.1. Management and treatment of malaria in all subjects

All children with suspected malaria will have a blood sample taken for confirmation of infection. Children with malaria that can be treated with oral medication will receive a 6-dose regimen of Coartem® (artemether/lumefantrine, Novartis AG, Zurich, Switzerland), as advocated by WHO [World Health Organization 2005] and the government of Gabon.

Children who require inpatient care or systemic treatment will receive treatment with intravenous quinine, which is standard practice and effective therapy in Gabon.

5.8.1.1. Insecticide impregnated bednets

An insecticide impregnated bednet will be offered to all parent(s)/guardian(s) who present their child for screening regardless of whether or not their child takes part in the trial. Parents/guardians will be instructed on the proper use and maintenance of insecticide impregnated bednets.

5.9. Subject identification

Subject numbers will be issued sequentially to subjects at screening. Treatment numbers are assigned at the time of Dose 1 in order of administration of dose.

Subject numbers will be issued consecutively to all children who are consented for screening. To identify the child at subsequent contacts, each child's parent(s)/guardian(s) will be issued with an identification card with the picture of the parent(s)/guardian(s) with the child attached to it.

Vials of RTS,S/AS02D and RTS,S/AS01E will be identified by a treatment number. The unique treatment number will identify all doses of the vaccine administered to each subject. The treatment number will be issued sequentially at Clinic Visit 2. The treatment number will be recorded on the clinic form and subsequently the case report form documenting the linkage between the subject number and the treatment number.

5.10. Outline of study procedures

Table 3 List of study procedures

	SCREEN	DOUBLE-BLIND PHASE								SINGLE-BLIND PHASE	
**Study Month**		Month 0			Month 1		Month 2		Month 3	Month 4 to 13	Month 14
**Study Day**	-28 to 0	0	1 to 5	6	30	31 to 36	60	61 to 66	90		
**Clinic Visit**	1	2		3	4		5		6		7
**Field Worker Visit Code #**			21 to 25			26 to 31		32 to 37		38 to 47	
**STUDY PROCEDURES**											
Informed consent	●										
Medical history	○	●									
Record HBV immunization status	●										
Vital signs	○	●		○	○		○		○		○
Complete physical examination	○	●									
Measure body weight	○	●			○		○		○		○
Assign Subject Number	●										
Prevaccination temperature		●			●		●				
Check inclusion/exclusion criteria	●	○									
Check elimination criteria		●		●	●		●		●		●
Check contraindications to vaccination		●			●		●				
Randomization		●									
Administer RTS,S/AS01E or RTS,S/AS02D vaccine		●			●		●				
Recording of concomitant medication		● [c]	● [c]	● [c]	● [c]	● [c]	● [c]	● [c]	● [c]	● [d]	● [d]
**SAFETY DATA COLLECTION**											
Recording of solicited symptoms (Investigator)		●		●	●		●				
Recording of solicited symptoms (Field Workers)			●			●		●			
Recording of unsolicited AEs within 1 month post-vaccination (Investigator)		●		●	●		●		●		
Morbidity surveillance/recording of SAEs	●[b]	●	●	●	●	●	●	●	●	●	●
Monthly field worker home visits										●	
**SAFETY LABS**											
Complete blood count [a]	●			●					●		●
Creatinine, ALT, bilirubin	●			●					●		●
**INVESTIGATIONAL ASSAYS**											
Anti-HBs antibodies	●						●		●		●
Anti-CS antibodies	●						●		●		●
Cell-Mediated Immunity and B-cell Memory	●						●		●		●
**FINAL ANALYSIS**									●		
**STUDY CONCLUSION**											●

#: Field Worker visit code numbers not necessarily sequential. • is used to indicate a study procedure that requires documentation in the individual CRF and ○ is used to indicate a study procedure that does not require documentation in the individual CRF.
a: includes analysis of hemoglobin, total white cell count and platelets
b: SAEs related to study procedures will be collected
c: all medications required by protocol to be recorded at these visits
d: record administration of immunosuppressants or other immune-modifying drugs during this period (for corticosteroids this means prednisone or equivalent, ≥ 0.5 mg/kg/day. Inhaled or topical steroids are allowed and should not be recorded. ALSO all immunoglobulins and blood products should be recorded during this period.

It is the investigator's responsibility to ensure that the intervals between visits/contacts are strictly followed. These intervals determine each subject's evaluability in the according to protocol analyses (see Sections 4.6 to 4.9 and Section 10.6 for details of criteria for evaluability and cohorts to be analyzed). The intervals are tabulated in Table 4.

Table 4 Intervals between study stages/visits

Interval	Length of interval
(Visit 1→Visit 2)	Screening –28 to Day –0
(Visit 2→Visit 4)	21 to 35 days
(Visit 4→Visit 5)	21 to 35 days
(Visit 2→Visit 7)	14 months ± 1 month

5.11. Detailed description of study stages/visits

When materials are provided by GSK Biologicals, it is **MANDATORY** that all clinical samples (including serum samples) will be collected and stored using exclusively those materials in the appropriate manner. The use of other materials could result in the exclusion of the subject from the ATP analysis (See Section 10.6 for definition of study cohorts to be evaluated). The investigator must ensure that his/her personnel and the laboratory(ies) under his/her supervision comply with this requirement. However, when GSK Biologicals does not provide material for collecting and storing clinical samples, then appropriate materials from the investigator's site are to be used. Refer to Appendix D and Appendix E.

Clinic Visit 1: Screening
Week -4 to Day 0

- Obtain signed, dated, thumb printed informed consent from the parent(s)/guardians
- Check inclusion/exclusion criteria
- Record HBV immunization status
- Take medical history and carry out physical examination
- Record any SAEs that may have occurred as a result of study procedures
- Venous blood sample to collect 5 mL blood for analysis of:
  - hematology (complete blood count)
  - biochemistry (creatinine, ALT and bilirubin)
  - serology (antibodies to CS, HBs)
  - cell-mediated immunity and B-cell memory

Clinic Visit 2: Vaccination 1
Day 0

- Check study identification card of vaccinee
- Take medical history and carry out physical examination, measure body weight
- Check contraindications to vaccination
- Check elimination criteria
- Record pre-vaccination body temperature

- Randomize subjects
- Administer the first dose of RTS,S/AS01E or RTS,S/AS02D intramuscularly in the left deltoid

Each child will be assessed for at least 60 minutes after vaccination to evaluate and treat any acute adverse events.

- Record any post-vaccination solicited symptoms
- Record any post-vaccination unsolicited adverse events
- Record any post-vaccination SAEs
- Record concomitant medication

Field worker post vaccination follow-up visits 21 to 25
Days 1, 2, 3, 4, and 5

- Record axillary temperature of subject
- Record local (pain and swelling at the injection site) and general (fever, irritability / fussiness, drowsiness, loss of appetite) solicited adverse events
- Record SAEs experienced by the vaccinee since the last visit
- Record unsolicited adverse events experienced by the vaccinee since the last visit
- Record concomitant medication

Clinic Visit 3: Post Dose 1 follow-up visit in Clinic
Day 6

- Check study identification card of vaccinee
- Carry out physical examination
- Record axillary temperature of subject
- Record local (pain and swelling at the injection site) and general (fever, irritability / fussiness, drowsiness, loss of appetite) solicited adverse events
- Record SAEs experienced by the vaccinee since the last visit
- Record unsolicited adverse events experienced by the vaccinee since the last visit
- Check elimination criteria
- Record concomitant medication
- Blood sample to collect a minimum 1 mL blood for analysis of:
  - hematology (complete blood count)
  - biochemistry (creatinine, ALT and bilirubin)

Clinic Visit 4: Vaccination 2
Day 30

- Check study identification card of vaccinee
- Check contraindications to vaccination
- Check elimination criteria
- Record unsolicited adverse events experienced by the vaccinee since the last visit
- Record pre-vaccination body temperature
- Administer the second dose of RTS,S/AS01E or RTS,S/AS02D intramuscularly in the left deltoid

Each child will be assessed for at least 60 minutes after vaccination to evaluate and treat any acute adverse events.

- Record any post-vaccination solicited symptoms
- Record any post-vaccination unsolicited adverse events
- Record any post-vaccination SAEs
- Record concomitant medication

Field worker post vaccination follow-up visits 26 to 31
Days 31, 32, 33, 34, 35 and 36

- Record axillary temperature of subject
- Record local (pain and swelling at the injection site) and general (fever, irritability / fussiness, drowsiness, loss of appetite) solicited adverse events
- Record SAEs experienced by the vaccinee since the last visit
- Record unsolicited adverse events experienced by the vaccinee since the last visit
- Record concomitant medication

Clinic Visit 5: Vaccination 3
Day 60

- Check study identification card of vaccinee
- Check contraindications to vaccination
- Check elimination criteria
- Record unsolicited adverse events experienced by the vaccinee since the last visit
- Record pre-vaccination body temperature
- Administer the third dose of RTS,S/AS01E or RTS,S/AS02D intramuscularly in the left deltoid

Each child will be assessed for at least 60 minutes after vaccination to evaluate and treat any acute adverse events.

- Record any post-vaccination solicited symptoms
- Record any post-vaccination unsolicited adverse events
- Record any post-vaccination SAEs
- Record concomitant medication
- Blood sample to collect 5 mL blood for analysis of:
  - serology (antibodies to CS, HBs)
  - cell-mediated immunity and B-cell memory

Field worker post vaccination follow-up visits 32 to 37
Days 61, 62, 63, 64, 65 and 66

- Record axillary temperature of subject
- Record local (pain and swelling at the injection site) and general (fever, irritability / fussiness, drowsiness, loss of appetite) solicited adverse events
- Record SAEs experienced by the vaccinee since the last visit
- Record unsolicited adverse events experienced by the vaccinee since the last visit
- Record concomitant medication

Clinic Visit 6
Day 90

- Check study identification card of vaccinee
- Carry out physical examination
- Record axillary temperature of subject
- Record SAEs experienced by the vaccinee since the last visit
- Record unsolicited adverse events experienced by the vaccinee since the last visit
- Check elimination criteria
- Record concomitant medication
- Venous blood sample to collect 5 mL blood for analysis of:
  - hematology (complete blood count)
  - biochemistry (creatinine, ALT and bilirubin)
  - serology (antibodies to CS, HBs)
  - cell-mediated immunity and B-cell memory

Field worker post vaccination follow-up visits 38 to 47
Months 4, 5, 6, 7, 8, 9, 10, 11, 12, 13

- Record axillary temperature of subject
- Record concomitant medication

- Record SAEs experienced by the vaccinee since the last visit

Clinic Visit 7: final study visit
Month 14

- Record axillary temperature of subject
- Check elimination criteria
- Record concomitant medication
- Record SAEs experienced by the vaccinee since the last visit
- Venous blood sample to collect 5 mL blood for analysis of:
  - hematology (complete blood count)
  - biochemistry (creatinine, ALT and bilirubin)
  - serology (antibodies to CS, HBs)
  - cell-mediated immunity and B-cell memory

5.12. Sample handling and analysis

5.12.1. Treatment and storage of biological samples

See Appendix D of the protocol for details of treatment and storage of biological samples.

See Appendix E for instructions for shipment of biological samples.

5.12.2. Laboratory assays

At the investigator center (trial site), the following will be performed:

- Hematology and biochemistry determination
- Separation of serum from the blood samples, and freezing for shipment
- Freezing of sample for CMI, for shipment
- B-cell memory assays

Table 5 Laboratory immunological assays to be performed

Assay	Marker	Assay method	Test Kit/ Manufacturer	Assay unit	Assay cut-off	Laboratory
Anti-CS antibodies	R32LR	ELISA	In-house ELISA*	EU/mL	0.5	Leroux-Roels Laboratory, CEVAC, Ghent, Belgium
Anti-HBs antibodies		EIA	AUSAB EIA ABBOTT*	mIU/mL	10**	GSK Biologicals†, Rixensart, Belgium
Cell-mediated immunity		ICS	In-house ICS	% cytokine positive cells		GSK Biologicals†, Rixensart, Belgium
B-cell memory		B-cell Elispot	In-house assay	% antibody producing cells		HAS MRU

*: or equivalent
**: seroprotective level
†: or designated validated laboratory
ELISA: Enzyme-linked Immunoabsorbent Assay
EIA: Enzyme immunoassay
CEVAC: Center for Vaccinology, Ghent University
ICS: Intra-cellular cytokine staining
ELISPOT: Enzyme-linked Immuno-Spot

The GSK Biologicals' laboratory at Rixensart has established Quality Control Procedures and an established Quality System. Both are audited regularly for quality assessment by an internal (sponsor-dependent) but laboratory-independent Quality Department to document the competency of the facility to perform the required tests and support the reliability of the results. Methods and equipment are validated, where required.

5.12.3. Serology, CMI and B-cell memory plan

Serum for antibody determination, CMI and B-cell memory will be collected by blood sample. Details of the assay methodology can be found in Appendix F. Samples for safety will be analyzed at the time they are collected. All samples will be kept in a secure locked area at the investigator's site or GSK Biologicals (or designated validated laboratory). All samples will only be made available to authorized personnel.

In order to spare blood volumes collected from the children, CMI and B-cell memory immune responses will not be determined in the same children. Half of the children will have CMI assessed, and the other half will have B-cell memory immune response characterized. Attribution will be determined by a randomization list (1:1), balanced by study group.

Serology will be assessed in all children.

Serum and CMI samples will be shipped to GSK Biologicals, Rixensart, Belgium.

Any serum and cells not immediately used in antibody assays will be stored at -20°C or less. Stored samples could be anonymized and used to assess the immune response to vaccination or to assess any potential toxicity of the vaccine (details of serology testing

are given in Appendix F). All serum/cells samples will be destroyed within ten years of study completion.

Table 6 Summary of blood sampling time points/immunological assays

Blood sampling time point			Test	No. subjects	Laboratory	Priority ranking
**Timing**	**Study Month**	**Clinic Visit No.**				
Screening	Screening	1	Anti-CS antibodies	180	GSK Biologicals*	1
			Anti-HBs antibodies	180	GSK Biologicals*	2
			CMI	90	GSK Biologicals*	3
			B-cell memory	90	HAS MRU	4
Post Dose 2	Study Month 2	5	Anti-CS antibodies	180	GSK Biologicals*	1
			Anti-HBs antibodies	180	GSK Biologicals*	2
			CMI	90	GSK Biologicals*	3
			B-cell memory	90	HAS MRU	4
Post Dose 3	Study Month 3	6	Anti-CS antibodies	180	GSK Biologicals*	1
			Anti-HBs antibodies	180	GSK Biologicals*	2
			CMI	90	GSK Biologicals*	3
			B-cell memory	90	HAS MRU	4
Post Dose 3	Study Month 14	7	Anti-CS antibodies	180	GSK Biologicals*	1
			Anti-HBs antibodies	180	GSK Biologicals*	2
			CMI	90	GSK Biologicals*	3
			B-cell memory	90	HAS MRU	4

*: or designated laboratory

6. INVESTIGATIONAL PRODUCTS AND ADMINISTRATION

6.1. Study vaccines

The candidate vaccines to be used have been developed and manufactured by GSK Biologicals. A summary of details relating to the study vaccines can be found in Table 7.

The Quality Control Standards and Requirements for the candidate vaccine are described in separate release protocols and the required approvals have been obtained.

Refer to Appendix G for details of vaccine supplies.

Table 7 Details of study vaccines to be administered during trial

Vaccine	Formulation	Presentation	Volume	N° doses*
RTS,S/AS01E	RTS,S 25 µg<br>MPL 25 µg<br>QS21 25 µg<br>Sucrose<br>Liposomes	RTS,S and sucrose pellet in 3 mL monodose vial<br>Adjuvant provided in monodose vial<br>Opalescent reconstituted liquid	0.5 mL	Sufficient for 90 subjects
RTS,S/AS02D	RTS,S 25 µg<br>MPL 25 µg<br>QS21 25 µg<br>Sucrose<br>Oil-in-water emulsion	RTS,S and sucrose pellet in 3 mL monodose vial. Prefilled adjuvant syringes. Milky reconstituted liquid	0.5 mL	Sufficient for 90 subjects

*: In addition to the vaccine doses provided for the planned number of enrolled subjects, 3% additional doses will be supplied in case a vaccine dose is broken or unusable

6.1.1. RTS,S/AS01E vaccine

The final RTS,S/AS01E vaccine consists of two fractions: the lyophilized fraction containing the RTS,S antigen in one vial and the liquid fraction, consisting of AS01E adjuvant in the other vial to be used for reconstitution just prior to injection. A dose of 0.5 mL will be injected. The presentation of the reconstituted RTS,S/AS01E candidate malaria vaccine is an opalescent liquid.

6.1.1.1. RTS,S antigen presentation:

- The RTS;S antigen presents as a lyophilized pellet containing approximately 25 µg of antigen with sucrose as cryoprotectant per 3 mL monodose vial.

6.1.1.2. AS01E adjuvant:

- AS01E contains 25 µg of MPL, 25 µg QS21 (QS21 is a triterpene glycoside purified from the bark of *Quillaja saponaria*) with liposomes.

6.1.2. RTS,S/AS02D vaccine

The final RTS,S/AS02D vaccine consists of two fractions: the lyophilized fraction containing the RTS,S antigen in one vial and the liquid fraction, consisting of AS02D adjuvant in pre-filled syringes to be used for reconstitution just prior to injection. A dose of 0.5 mL will be delivered. The presentation of the reconstituted RTS,S/AS02D candidate malaria is an opaque milky liquid.

6.1.2.1. RTS,S antigen presentation:

- The RTS;S antigen presents as a lyophilized pellet containing approximately 25 µg of antigen with sucrose as cryoprotectant per 3 mL monodose vial.

6.1.2.2. AS02D adjuvant:

- AS02D contains 25 µg of MPL, 25 µg QS21 (QS21 is a triterpene glycoside purified from the bark of *Quillaja saponaria*) and 125 µl of a proprietary oil-in-water emulsion and phosphate buffered saline per 0.5 mL, presented in prefilled syringes (PFS).

6.2. Dosage and administration

The vaccinees will be observed closely for at least 60 minutes following the administration of vaccines, with appropriate medical treatment readily available in case of an anaphylactic reaction.

6.2.1. RTS,S/AS01E

RTS,S/AS01E will be supplied such that the reconstituted vaccine volume will provide a 0.5 mL pediatric dose. One 0.5 mL dose will be withdrawn from each vial and used.

Disinfect top of vaccine vial (pellet) and adjuvant vial with alcohol swabs and let dry. Withdraw the contents of the adjuvant vial in a syringe and inject adjuvant into the vial of lyophilized antigen. Remove and discard the syringe and needle under appropriate safety precautions. The pellet is then dissolved by gently shaking the vial. Wait for 1 minute to ensure complete dissolution of vial contents before withdrawing a sufficient volume to provide a 0.5 mL dose (volume required for RTS,S/AS01E) of the reconstituted vaccine solution using a fresh needle and syringe for injection. The reconstituted vaccine should be administered by slow intramuscular (IM) injection, using a 25G needle with length of 1 inch (25 mm), in the left deltoid muscle within 4 hours of reconstitution (storage at +2°C to +8°C).

6.2.2. RTS,S/AS02D

RTS,S/AS02D will be supplied such that the reconstituted vaccine volume will provide a 0.5 mL pediatric dose. One 0.5 mL dose will be withdrawn from each vial and used.

Disinfect top of vaccine vial (pellet) with alcohol swabs and let dry. Inject complete contents of one PFS of adjuvant into vial of lyophilized vaccine. Remove and discard the syringe and needle under appropriate safety precautions. The pellet is then dissolved by gently shaking the vial. Wait for 1 minute to ensure complete dissolution of vial contents before withdrawing a sufficient volume to provide a 0.5 mL dose (volume required for RTS,S/AS02D) of the reconstituted vaccine solution using a fresh needle and syringe for injection. The reconstituted vaccine should be administered by slow IM injection, using a 25G needle with length of 1 inch (25 mm), in the left deltoid muscle within 4 hours of reconstitution (storage at 2°C to 8°C).

6.3. Storage

ALL VACCINE VIALS/PRE-FILLED SYRINGES AND ADJUVANTS MUST BE STORED IN THE REFRIGERATOR (+2°C to +8°C) AND MUST NOT BE FROZEN.

All vaccines will be stored in a safe and locked place with no access for unauthorized personnel. Storage temperature will be monitored three times daily, according to SOPs at the investigator's site. An alarm system and a back-up refrigerator will be available in case of power failure/breakdown.

The study monitor must be contacted if the cold chain is broken (e.g. vaccines become frozen or refrigeration fails).

Storage conditions for transport of vaccines from country medical department or dispatch center to study sites or between sites are described in Appendix D.

6.4. Treatment allocation and randomization

6.4.1. Randomization of supplies

A randomization list will be generated at GSK Biologicals, Rixensart, using a standard SAS® (Statistical Analysis System) program and will be used to assign the vaccines in a 1:1 fashion to treatment numbers.

6.4.2. Randomization of subjects

Subjects will be allocated sequentially to treatment numbers in the order that they present for vaccination.

6.5. Method of blinding and breaking the study blind

Data pertaining to RTS,S/AS02D or RTS,S/AS01E will be collected in a double blinded (observer blinded) manner. 'Double blinded (observer blinded)' means that the vaccine recipient and their parent(s)/guardian(s) as well as those responsible for the evaluation of safety and immunogenicity endpoints will all be unaware which treatment, RTS,S/AS02D or RTS,S/AS01E, was administered to a particular subject. The only study staff aware of the vaccine assignment for RTS,S/AS02D or RTS,S/AS01E will be those responsible for the storage and preparation of vaccines; these staff will play no other role in the study.

Code break envelopes, for each study enrolled subject and associating each treatment number with a specific vaccine, will be kept by the Local Safety Monitor in Gabon as well as by Central Safety at GSK Biologicals, Rixensart in a safe and locked place with no access for unauthorized personnel.

If deemed necessary for reasons such as safety, the Local Safety Monitor in Gabon as well as GSK Biologicals Central Safety will unblind the specific enrolled subject without revealing the study blind to the investigators.

As part of the safety monitoring plan, safety reports will be produced at two timepoints in the trial (refer to 5.1.3.3). An independent statistician will analyze the data, thereby maintaining the blind of the malaria project team at GSK Biologicals and the investigator group.

A formal reporting and analysis plan (RAP) will be developed. Once the study is completed and the GSK Biologicals reference database locked, GSK Biologicals will be responsible for initiating the execution of the statistical analysis plan in collaboration with HAS and MVI and breaking the blind.

GSK Biologicals' policy (incorporating ICH E2A guidance, EU Clinical Trial Directive and Federal Regulations) is to unblind any serious adverse event (SAE) report associated with the use of the investigational product, which is unexpected and attributable/suspected, prior to regulatory reporting. The Clinical Safety Physician is responsible for unblinding the treatment assignment in accordance with specified time frames for expedited reporting of SAEs (Refer to Section 8.8).

6.6. Replacement of unusable vaccine doses

Additional vaccine doses will be provided to replace those that are unusable (see Appendix G for details of supplies).

In addition to the vaccine doses provided for the planned number of enrolled subjects, 3% additional doses will be supplied. In case a vaccine dose is broken or unusable, the investigator should replace it with a replacement vaccine dose. If a vaccine dose needs replacement, the envelope with the corresponding treatment number will designate the replacement without unblinding the study using a coded letter system. Although the sponsor need not be notified immediately in these cases, documentation of the use of the replacement vaccine must be recorded by the investigator on the vaccine administration page of the CRF and on the vaccine accountability form.

6.7. Packaging

See Appendix G.

6.8. Vaccine accountability

See Appendix G.

6.9. Concomitant medication/treatment

At each study visit/contact, the investigator should question the enrolled subject's parent(s)/guardian(s) about any medication(s) taken.

All antipyretic, analgesic and antibiotic drugs, administered at ANY time during the period starting with administration of each dose and ending 30 days after each dose of vaccine are to be recorded with generic name of the medication (trade names are allowed for combination drugs, i.e., multi-component drugs), medical indication, total daily dose, route of administration, start and end dates of treatment.

Any treatments and/or medications which are listed as elimination criteria in Section 4.8, e.g., any immunoglobulins, other blood products and any immune modifying drugs administered within three months preceding the first dose or at any time during the study period are to be recorded with generic name of the medication (trade names are allowed for combination drugs only), medical indication, total daily dose, route of administration, start and end dates of treatment. Refer to Sections 4.7 to 4.9. The time periods between which each type of concomitant medication/treatment should be recorded is summarized in Table 8.

Table 8 Summary of time periods between which different classes of concomitant medication/treatment/vaccination must be recorded

**3 months prior to Dose 1 → Dose 1**	All treatments listed as elimination criteria in Section 4.8*
**Screening → 30 Days post Dose 3**	All antipyretic, analgesic, antibiotic and any treatments listed as elimination criteria in Section 4.8*<br>All vaccinations
**31 Days post Dose 3 → Final Study Visit**	All treatments listed as elimination criteria in Section 4.8*

* e.g. any immunoglobulins, other blood products and any immune modifying drugs

Any vaccine not foreseen in the study protocol administered in the period beginning 30 days preceding each dose and ending 30 days after each dose is to be recorded with trade name, route of administration and date(s) of administration. Refer to Sections 4.7 to 4.9.

Any concomitant medication administered prophylactically in anticipation of reaction to the vaccination must be recorded in the CRF with generic name of the medication (trade names are allowed for combination drugs only), total daily dose, route of administration, start and end dates of treatment and coded as ‘Prophylactic’.

7. HEALTH ECONOMICS

Not applicable

8. ADVERSE EVENTS AND SERIOUS ADVERSE EVENTS

The investigator is responsible for the detection and documentation of events meeting the criteria and definition of an adverse event (AE) or serious adverse event (SAE) as provided in this protocol. During the study, when there is a safety evaluation, the investigator or site staff will be responsible for detecting AEs and SAEs, as detailed in this section of the protocol.

Each subject/ subject’s parents/guardians will be instructed to contact the investigator immediately should they/ the subject manifest any signs or symptoms they perceive as serious.

8.1. Definition of an adverse event

An AE is any untoward medical occurrence in a clinical investigation subject, temporally associated with the use of a medicinal product, whether or not considered related to the medicinal product.

An AE can therefore be any unfavorable and unintended sign (including an abnormal laboratory finding), symptom, or disease (new or exacerbated) temporally associated with the use of a medicinal product.

Examples of an AE include:

- Exacerbation of a chronic or intermittent pre-existing condition including either an increase in frequency and/or intensity of the condition.
- New conditions detected or diagnosed after investigational product administration even though it may have been present prior to the start of the study.
- Signs, symptoms, or the clinical sequelae of a suspected overdose of either investigational product or a concurrent medication (overdose per se should not be reported as an AE/SAE).
- Signs, symptoms temporally associated with vaccine administration.

AEs may include pre- or post-treatment events that occur as a result of protocol-mandated procedures (i.e., invasive procedures, modification of subject's previous therapeutic regimen).

N.B. AEs to be recorded as endpoints (solicited events) are described in Section 8.4.1. All other AEs will be recorded as **UNSOLICITED AEs**.

Example of events to be recorded in the medical history section of the CRF:

- Pre-existing conditions or signs and/or symptoms present in a subject prior to the start of the study (i.e. prior to the first study procedure) should be recorded in the medical history section of the subject's CRF.

8.2. Definition of a serious adverse event

A serious adverse event (SAE) is any untoward medical occurrence that:

a. results in death;

b. is life-threatening;

NOTE: The term 'life-threatening' in the definition of 'serious' refers to an event in which the subject was at risk of death at the time of the event. It does not refer to an event, which hypothetically might have caused death, if it were more severe.

c. requires hospitalization or prolongation of existing hospitalization;

NOTE: In general, hospitalization signifies that the subject has been detained (usually involving at least an overnight stay) at the hospital or emergency ward for observation

and/or treatment that would not have been appropriate in the physician's office or out-patient setting. Complications that occur during hospitalization are AEs. If a complication prolongs hospitalization or fulfills any other serious criteria, the event is serious. When in doubt as to whether 'hospitalization' occurred or was necessary, the AE should be considered serious.

Hospitalization for elective treatment of a pre-existing condition that did not worsen from baseline is not considered an AE.

d. results in disability/incapacity;

NOTE: The term disability means a substantial disruption of a person's ability to conduct normal life functions. This definition is not intended to include experiences of relatively minor medical significance such as uncomplicated headache, nausea, vomiting, diarrhea, influenza, and accidental trauma (e.g. sprained ankle) which may interfere or prevent everyday life functions but do not constitute a substantial disruption.

e. medical or scientific judgment should be exercised in deciding whether reporting is appropriate in other situations, such as important medical events that may not be immediately life-threatening or result in death or hospitalization but may jeopardize the subject or may require medical or surgical intervention to prevent one of the other outcomes listed in the above definition. These should also be considered serious. Examples of such events are invasive or malignant cancers, intensive treatment in an emergency room or at home for allergic bronchospasm, blood dyscrasias or convulsions that do not result in hospitalization.

In this study all seizures occurring within a 30-day period of vaccination will be notified as SAEs [refer to Bonhoeffer 2004]. Key information pertaining to seizures will be documented in the CRF.

8.3. Clinical laboratory parameters and other abnormal assessments qualifying as adverse events and serious adverse events

Abnormal laboratory findings (e.g., clinical chemistry, hematology, urinalysis) or other abnormal assessments (e.g. blood film) that are judged by the investigator to be clinically significant will be recorded as AEs or SAEs if they meet the definition of an AE, as defined in Section 8.1 or SAE, as defined in Section 8.2. Clinically significant abnormal laboratory findings or other abnormal assessments that are detected during the study or are present at baseline and significantly worsen following the start of the study will be reported as AEs or SAEs.

The investigator will exercise his medical and scientific judgment in deciding whether an abnormal laboratory finding or other abnormal assessment is clinically significant.

8.4. Time period, frequency, and method of detecting adverse events and serious adverse events

All AEs occurring within 30 days following administration of each dose of vaccine must be recorded on the Adverse Event form in the subject's CRF, irrespective of severity or whether or not they are considered vaccination-related.

The standard time period for collecting and recording SAEs will begin at first receipt of vaccine and will end at the study conclusion. See Section 8.7 for instructions for reporting and recording SAEs.

Additionally, in order to fulfill international reporting obligations, SAEs that are related to study participation (e.g. procedures, vaccinations, invasive tests, transport to and from study visits) or are related to a concurrent medication will be collected and recorded from the time the subject consents to participate in the study until she/he is discharged: if a child is screened and enrolled, then discharge will be the end of the study as per study protocol; if a child is screened but not enrolled, then discharge will be the point at which the decision is taken not to enroll the child.

The investigator will inquire about the occurrence of AEs/SAEs at every visit/contact during the study and throughout the follow-up phase as appropriate.

The mechanism by which SAEs will be identified in the study are detailed in Section 5.7. The investigator or study clinician will fully document any such events on the Serious Adverse Event pages appended to the individual Case Report Form including, where applicable, information from relevant hospital case records, autopsy reports and verbal autopsies.

All AEs either observed by the investigator, study clinician, field worker or reported by the subject's parent/guardian spontaneously or in response to a direct question will be evaluated by the investigator. AEs not previously documented in the study will be recorded in the Adverse Event form within the subject's CRF. The nature of each event, date and time (where appropriate) of onset, outcome, intensity and relationship to vaccination will be established. Details of any corrective treatment will be recorded on the appropriate page of the CRF. Refer to Section 6.9.

As a consistent method of determining the occurrence of unsolicited AEs, the subject or the subject's parent/guardian will be asked a non-leading question such as:

'Has your child acted differently or felt different in any way since receiving the vaccine or since the last visit '

N.B. The investigator should record only those AEs having occurred within the time frame defined above.

AEs already documented in the CRF, i.e. at a previous assessment, and designated as 'not recovered/not resolved' or 'recovering/resolving' should be reviewed at subsequent visits, as necessary. If these have resolved, the documentation in the CRF should be completed.

When an AE/SAE occurs, it is the responsibility of the investigator to review all documentation (e.g., hospital progress notes, laboratory, verbal autopsies and diagnostics reports) relative to the event. The investigator will then record all relevant information regarding an AE/SAE on the CRF or SAE Report Form as applicable. It is not acceptable for the investigator to send photocopies of the subject's medical records to GSK Biologicals in lieu of the appropriate completed AE/SAE pages. However, there may be instances when copies of medical records and verbal autopsies for certain cases are requested by GSK Biologicals. In this instance, all subject identifiers will be blinded on the copies of the medical records prior to submission to GSK Biologicals.

The investigator will attempt to establish a diagnosis of the event based on signs, symptoms, and/or other clinical information. In such cases, the diagnosis should be documented as the AE/SAE and not the individual signs/symptoms.

8.4.1. Solicited adverse events

Local (injection site) adverse events

- Pain at injection site
- Swelling at injection site

General adverse events

- Fever (defined as axillary temperature ≥ 37.5°C)
- Drowsiness
- Loss of appetite
- Irritability/fussiness

The visiting field worker will record these adverse events according to detailed SOPs available on study site during the field worker visits.

N.B. Temperature will be recorded on days 1 to 6 following each vaccination by the field worker or Principal Investigator (or their delegate). Should additional temperature measurements be performed at other times of day, the highest temperature will be recorded.

8.5. Evaluating adverse events and serious adverse events

8.5.1. Assessment of intensity

Intensity of the following AEs will be assessed as described:

Table 9 Intensity scales for solicited symptoms in infants/toddlers and children less than 6 years of age

Adverse Event	Intensity grade	Parameter
Pain at injection site	0	Absent
	1	Minor reaction to touch
	2	Cries/protests on touch
	3	Cries when limb is moved/spontaneously painful
Swelling at injection site		Record greatest surface diameter in mm
Fever*		Record temperature in °C
Irritability/Fussiness	0	Behavior as usual
	1	Crying more than usual/ no effect on normal activity
	2	Crying more than usual/ interferes with normal activity
	3	Crying that cannot be comforted/ prevents normal activity
Drowsiness	0	Behavior as usual
	1	Drowsiness easily tolerated
	2	Drowsiness that interferes with normal activity
	3	Drowsiness that prevents normal activity
Loss of appetite	0	Appetite as usual
	1	Eating less than usual/ no effect on normal activity
	2	Eating less than usual/ interferes with normal activity
	3	Not eating at all

*Fever is defined as axillary temperature ≥ 37.5°C

The maximum intensity of local injection site swelling will be scored at GSK Biologicals as follows:

0 : None
1 : < 5 mm
2 : 5 to 20 mm
3 : > 20 mm

The maximum intensity of fever will be scored at GSK Biologicals as follows:

0 : < 37.5°C
1 : 37.5 – 38.0°C
2 : > 38 – 39.0°C
3 : > 39.0°C

Table 10 Acceptable/normal ranges for blood testing

	Acceptable limit/ normal range	Toxicity grading scale			
		Grade 1	Grade 2	Grade 3	Grade 4
Hemoglobin	≥ 8.0 g/dL	< ULN	< 6.0 g/dL	< 5.0 g/dL	< 5.0 g/dL & clinical signs of heart failure
Total white cell count†	≥ 4.0 x 10^3/µL<br>< 17 x 10^3/µL	2.5 to 4.0 x 10^3/µL	1.5 to 2.4 x 10^3/µL	1.0 to 1.4 x 10^3/µL	< 1.0 x 10^3/µL
Platelets†	≥ 100 x 10^3/µL	50 to 99 x 10^3/µL	25 to 49 x 10^3/µL	< 25 x 10^3/µL	< 25 x 10^3/µL & clinical signs of bleeding
ALT*	≤ 60 µmol/L	1.1 to 2.5 x ULN	2.6 to 5.0 x ULN	5.1 to 10.0 x ULN	> 10.0 x ULN
Creatinine*	≤ 60 µmol/L	1.1 to 1.5 x ULN	1.6 to 3.0 x ULN	3.1 to 6.0 x ULN	> 6.0 ULN or requires dialysis

† Grading scale adapted from Division of AIDS table for grading severity of adult and pediatric adverse events December 2004
* Grading scale adapted from WHO Toxicity Grading Scale for Determining Severity of Adverse Events, February 2003.
ULN: Upper Limit of Normal

The investigator will make an assessment of the maximum intensity that occurred over the duration of the event for all other AEs, i.e. unsolicited symptoms, including SAEs reported during the study. The assessment will be based on the investigator's clinical judgment. The intensity of each AE and SAE recorded in the CRF or SAE Report Form, as applicable, should be assigned to one of the following categories:

1 (mild) = An AE which is easily tolerated by the subject, causing minimal discomfort and not interfering with everyday activities.

2 (moderate) = An AE which is sufficiently discomforting to interfere with normal everyday activities.

3 (severe) = An AE which prevents normal, everyday activities. (In a young child, such an AE would, for example, prevent attendance at school/ kindergarten/ a day-care center and would cause the parents/ guardians to seek medical advice. In adults/ adolescents, such an AE would, for example, prevent attendance at work/ school and would necessitate the administration of corrective therapy.)

An AE that is assessed as Grade 3 (severe) should not be confused with a SAE. Grade 3 is a category utilized for rating the intensity of an event; and both AEs and SAEs can be assessed as Grade 3. An event is defined as 'serious' when it meets one of the pre-defined outcomes as described in Section 8.2.

8.5.2. Assessment of causality

The investigator is obligated to assess the relationship between investigational product and the occurrence of each AE/SAE. The investigator will use clinical judgment to

determine the relationship. Alternative causes, such as natural history of the underlying diseases, concomitant therapy, other risk factors and the temporal relationship of the event to the investigational product will be considered and investigated. The investigator will also consult the Investigator Brochure and/or Product Information, for marketed products, in the determination of his/her assessment.

There may be situations when a SAE has occurred and the investigator has minimal information to include in the initial report to GSK Biologicals. However, it is very important that the investigator always makes an assessment of causality for every event prior to transmission of the SAE Report Form to GSK Biologicals. The investigator may change his/her opinion of causality in light of follow-up information, amending the SAE Report Form accordingly. The causality assessment is one of the criteria used when determining regulatory reporting requirements.

In case of concomitant administration of multiple vaccines, it may not be possible to determine the causal relationship of general AEs to the individual vaccines administered. The investigator should, therefore, assess whether the AE could be causally related to vaccination rather than to the individual vaccines.

All solicited local (injection site) reactions will be considered causally related to vaccination. Causality of all other AEs should be assessed by the investigator using the following question:

Is there a reasonable possibility that the AE may have been caused by the investigational product ?

NO	:	The AE is not causally related to administration of the study vaccine(s). There are other, more likely causes and administration of the study vaccine(s) is not suspected to have contributed to the AE.
YES	:	There is a reasonable possibility that the vaccine(s) contributed to the AE.

Non-serious and serious AEs will be evaluated as two distinct events. If an event meets the criteria to be determined “serious” (see Section 8.2 for definition of serious adverse event), it will be examined by the investigator to the extent to be able to determine ALL contributing factors applicable to each serious adverse event.

Other possible contributors include:

- Medical history
- Other medication
- Protocol required procedure
- Other procedure not required by the protocol
- Lack of efficacy of the vaccine(s), if applicable

- Erroneous administration
- Other cause (specify).

8.6. Follow-up of adverse events and serious adverse events and assessment of outcome

After the initial AE/SAE report, the investigator is required to proactively follow each subject and provide further information to GSK Biologicals on the subject's condition.

All AEs and SAEs documented at a previous visit/contact and designated as not recovered/not resolved or recovering/resolving will be reviewed at subsequent visits/contacts.

Investigators will follow-up subjects:

- with SAEs or subjects withdrawn from the study as a result of an AE, until the event has resolved, subsided, stabilized, disappeared, the event is otherwise explained, or the subject is lost to follow-up;
- or, in the case of other non-serious AEs, until they complete the study or they are lost to follow-up.

All Grade 3, Grade 4 or clinically significant laboratory abnormalities will be followed up until they have returned to normal, or a satisfactory explanation has been provided. Additional information (including but not limited to laboratory results) relative to the subsequent course of such an abnormality noted for any subject must be made available to the Study Monitor.

GSK Biologicals may request that the investigator perform or arrange for the conduct of supplemental measurements and/or evaluations to elucidate as fully as possible the nature and/or causality of the AE or SAE. The investigator is obliged to assist. If a subject dies during participation in the study or during a recognized follow-up period, GSK Biologicals will be provided with a copy of any available post-mortem findings, including histopathology.

New or updated information will be recorded on the originally completed SAE Report Form, with all changes signed and dated by the investigator. The updated SAE report form should be resent to GSK Biologicals within 24 hours of receipt of the follow-up information as outlined in Section 8.7.1.

Outcome of any non-serious AE occurring within 30 days post-vaccination (i.e. unsolicited AE) or any SAE reported during the entire study will be assessed as:

- Recovered/resolved
- Not recovered/not resolved
- Recovering/resolving
- Recovered with sequelae/resolved with sequelae

- Fatal (SAEs only).

8.7. Prompt reporting of serious adverse events to GSK Biologicals

8.7.1. Time frames for submitting serious adverse event reports to GSK Biologicals

SAEs will be reported promptly to GSK once the investigator determines that the event meets the protocol definition of an SAE. The investigator or designate will fax the SAE reports to GSK Biologicals' Study Contact for Serious Adverse Event Reporting **WITHIN 24 HOURS OF HIS BECOMING AWARE OF THESE EVENTS**. Additional or follow-up information relating to the initial SAE report is also to be reported to the GSK Biologicals' Study Contact for Serious Adverse Event Reporting within 24 hours of receipt of such information.

8.7.2. Completion and transmission of serious adverse event reports to GSK Biologicals

Once an investigator becomes aware that a SAE has occurred in a study subject, she/he will report the information to GSK within 24 hours as outlined in Section 8.7.1. The SAE Report Form will always be completed as thoroughly as possible with all available details of the event, signed by the investigator (or designee), and forwarded to GSK within the designated time frames. If the investigator does not have all information regarding an SAE, he/she will not wait to receive additional information before notifying GSK of the event and completing the form. The form will be updated when additional information is received and forwarded to GSK **WITHIN 24 HOURS** as outlined in Section 8.7.1.

The investigator will always provide an assessment of causality at the time of the initial report as described in Section 8.5.2.

Facsimile (Fax) or electronic transmission of the SAE Report Form are the preferred methods to transmit this information to the Study Contact for Reporting SAEs. In rare circumstances and in the absence of facsimile equipment or electronic connection, notification by telephone is acceptable, with a copy of the SAE Report Form to follow. Initial notification via the telephone does not replace the need for the investigator to complete and sign the SAE Report Form within 24 hours as outlined in Section 8.7.1.

In the event of a death determined by the investigator to be related to vaccination, sending of the fax must be accompanied by telephone call to the Study Contact for Reporting SAEs.

Study Contact for Reporting of a Serious Adverse Event

All four of the contacts listed below must be informed of each SAE

Study Contact at GSK Biologicals for Reporting Serious Adverse Events Manager Clinical Safety Vaccines

GSK Biologicals Clinical Safety Physician,
GlaxoSmithKline Biologicals,
Rue de l'Institut 89, 1330 Rixensart,
Belgium.
Tel: +32.2.656.87.98
Fax: +32.2.656.80.09
Mobile phone for 7/7 day availability:
+32.477.40.47.13
email: rix.ct-safety-vac@gskbio.com

Central Study Coordinator

Isabelle Ramboer,
Central Study Coordinator,
GlaxoSmithKline Biologicals,
Rue de l'Institut 89, 1330 Rixensart,
Belgium.
Tel: +32.2.656.68.20
Fax: +32.2.656.80.44
email: isabelle.ramboer@gskbio.com

Malaria Vaccine Initiative

Carolyn Petersen,
Malaria Vaccine Initiative, PATH,
7500 Old Georgetown Road, 12th Floor,
Bethesda, MD 20814,
USA.
Tel: +1.240.395.27.16
or Tel: +1.240.395.27.00
Fax: +1.240.395.25.91
email: sae@malariavaccine.org

alternate email:
cpetersen@malariavaccine.org

Local Safety Monitor

~~Jenny Dörnemann,~~ Grégoire Adzoda
Hôpital Albert Schweitzer,
B.P.~~ 118, Lambaréné,~~ ***13901, Libreville***
Gabon.

Tel: ~~+241.077.79.225~~
+00 241 07 35 83 33.
Fax: +241.581.196
~~email: jennydoernemann@gmx.de~~
gadzoda@yahoo.fr

Administrative Change, 16 February 2006

8.8. Regulatory reporting requirements for serious adverse events

The investigator will promptly report all SAEs to GSK in accordance with the procedures detailed in Section 8.7. GSK Biologicals has a legal responsibility to promptly notify, as appropriate, both the local regulatory authority and other regulatory agencies about the safety of a product under clinical investigation. Prompt notification of SAEs by the investigator to the Study Contact for Reporting SAEs is essential so that legal obligations and ethical responsibilities towards the safety of other subjects are met.

The investigator, or responsible person according to local requirements, will comply with the applicable local regulatory requirements related to the reporting of SAEs to the IRB/IEC and, if required, to the applicable government authority.

Investigator safety reports are prepared according to the current GSK policy and are forwarded to investigators as necessary. An investigator safety report is prepared for a SAE(s) that is both attributable to investigational product and unexpected. The purpose of the report is to fulfil specific regulatory and Good Clinical Practice (GCP) requirements, regarding the product under investigation.

An investigator who receives an investigator safety report describing a SAE(s) or other specific safety information (e.g., summary or listing of SAEs) from GSK Biologicals will file it with the Investigator Brochure or other appropriate study documentation and will notify the IRB or IEC, if appropriate according to local requirements.

8.9. Post-study adverse events and serious adverse events

A post-study AE/SAE is defined as any event that occurs outside of the AE/SAE detection period defined in Section 8.4. Investigators are not obligated to actively seek AEs or SAEs in former study participants.

However, if the investigator learns of any SAE, including a death, at any time after a subject has been discharged from the study, and he/she considers the event reasonably related to the investigational product, the investigator will promptly notify the Study Contact for Reporting SAEs.

8.10. Pregnancy

Not Applicable

8.11. Treatment of adverse events

Treatment of any adverse event is at the sole discretion of the investigator and according to current good medical practice. Any medication administered for the treatment of an AE should be recorded in the subject's CRF. Refer to Section 6.9.

9. SUBJECT COMPLETION AND WITHDRAWAL

9.1. Subject completion

A subject who returns for the concluding visit/is available for the concluding contact foreseen in the protocol is considered to have completed the study.

A subject that comes for Clinic Visit 6 has completed the double-blind phase of the study. A subject that comes for Clinic Visit 7 has completed the single-blind phase of the study.

9.2. Subject withdrawal

Subjects who are withdrawn for AEs must be clearly distinguished from subjects who are withdrawn for other reasons. Investigators will follow subjects who are withdrawn as

result of a SAE/AE until resolution of the event (see Section 8.6). Withdrawals will not be replaced.

9.2.1. Subject withdrawal from the study

From an analysis perspective, a 'withdrawal' from the study is any subject who was not available for the concluding contact foreseen in the protocol.

A subject qualifies as a 'withdrawal' from the study when no study procedure has occurred, no follow-up has been performed and no further information has been collected for this subject from the date of withdrawal/last contact.

Investigators will make an attempt to contact those subjects who do not return for scheduled visits or follow-up.

Information relative to the withdrawal will be documented on the Study Conclusion page of the CRF. The investigator will document whether the decision to withdraw from the study was made by the subject's parent or guardian or the investigator and which of the following possible reasons was responsible for withdrawal:

- serious adverse event
- non-serious adverse event
- protocol violation (specify)
- consent withdrawal, not due to an adverse event
- moved from the study area
- lost to follow-up
- death
- other (specify).

9.2.2. Subject withdrawal from investigational product

A 'withdrawal' from the investigational product is any subject who does not receive the complete treatment, i.e. when no further planned dose is administered from the date of withdrawal. A subject withdrawn from the investigational product may not necessarily be withdrawn from the study as further study procedures or follow-up may be performed (safety or immunogenicity) if planned in the study protocol.

Information relative to premature discontinuation of the investigational product will be documented on the Vaccine Administration page of the CRF. The investigator will document whether the decision to discontinue further vaccination/treatment was made by the subject's parent or guardian or the investigator and which of the following possible reasons was responsible for withdrawal:

- serious adverse event;
- non-serious adverse event;

- other (specify).

10. DATA EVALUATION: CRITERIA FOR EVALUATION OF OBJECTIVES

10.1. Coprimary endpoints

10.1.1. Safety

- Occurrence of SAEs from the time of first vaccination (Study Month 0) until one month post Dose 3 (Study Month 3).

10.1.2. Immunogenicity

- Anti-CS antibody titers one month post Dose 3.

10.2. Secondary endpoints

10.2.1. Safety and reactogenicity

- Occurrence of solicited general and local reactions over a 7-day follow-up period (day of vaccination and 6 subsequent days) after each vaccination.
- Occurrence of unsolicited symptoms after each vaccination over a 30-day follow-up period (day of vaccination and 29 subsequent days).

10.2.2. Immunogenicity

- Anti-CS antibody titers prior to vaccination, one month post Dose 2 and one month post Dose 3.
- Anti-HBs antibody titers prior to vaccination, one month post Dose 2 and one month post Dose 3.

10.3. Tertiary endpoints

10.3.1. Immunogenicity

At Study Month 14:

- Anti-HBs antibody titers determined 12 months post Dose 3.
- Anti-CS antibody titers determined at 12 months post Dose 3.

10.3.2. Safety

- Occurrence of SAEs from 1 month post Dose 3 until 12 months post Dose 3.

10.4. Exploratory endpoints

- Frequency of CS-specific T-cells prior to vaccination, one month post Dose 2, one month post Dose 3 and 12 months post Dose 3.
- Frequency of CS-specific B-cell memory prior to vaccination, one month post Dose 2, one month post Dose 3 and 12 months post Dose 3.
- Anti-CS antibody titers determined at 12 months post Dose 3 according to documented HBV immunization status at screening

10.5. Estimated sample size

A sample size of 75 evaluable subjects per group will have 90% power to demonstrate non-inferiority of RTS,S/AS02D versus RTS,S/AS01E in terms of anti-CS immune response (upper limit of 95% CI of the GMC ratio RTS,S/AS02D versus RTS,S/AS01E below 3.0) assuming a log Standard Deviation of 0.9 in both groups, alpha=0.025 (Pass 2000). It will be considered that non-inferiority is reached when the upper limit of the 95% CI of the anti-CS GMC ratio of RTS,S/AS02D to RTS,S/AS01E is below 3.0.

10.5.1. Sample size for the primary safety endpoint

Safety analyses will be performed on the Total Cohort. A trial of this size has the power to detect only large differences in the frequencies of AEs with reasonable power. A sample size of 75 evaluable subjects per group has the power to detect differences in the rates of safety endpoints between groups as shown in Table 11. Comparisons between groups will be done using Fisher's Exact test for each AE coded by preferred term (alpha = 0.05, 2 sided).

Table 11 Differences in safety endpoints (between treatment groups that can be detected with 90% power for varying baseline rates)

Frequency of events for RTS,S/AS02D	Frequency of events for RTS,S/AS01E	Power to detect difference (number per group equals 75)
1%	13%	90%
3%	18%	90%
5%	21%	90%
10%	29%	90%

10.6. Study cohorts to be evaluated

10.6.1. Total Vaccinated Cohort

The Total Vaccinated Cohort will include all vaccinated subjects for whom data are available. Thus, the total analysis of safety will include all subjects with at least one vaccine administration documented and the total analysis of immunogenicity will include vaccinated subjects for whom data concerning immunogenicity endpoint measures are available. The Total Vaccinated Cohort analysis will be performed per treatment actually administered.

10.6.2. According to protocol (ATP) cohort for analysis of safety

The ATP cohort for analysis of safety will include all evaluable subjects;

- who have received at least one dose of study vaccine according to their random assignment
- have sufficient data to perform an analysis of safety (at least one vaccine dose with safety follow-up)
- for whom administration site of study vaccine is per protocol
- who have not received a vaccine not specified or forbidden in the protocol and for whom elimination criteria were not applied
- for whom the randomization code has not been broken except for when unblinding has been carried out by the DSMB for Safety Analysis.
- who meet all eligibility criteria.

10.6.3. According to protocol (ATP) cohort for analysis of immunogenicity

The ATP cohort for analysis of immunogenicity will include all evaluable subjects (i.e. those meeting all eligibility criteria, complying with the procedures defined in the protocol, with no elimination criteria during the study) for whom data concerning immunogenicity endpoint measures are available.

10.7. Derived and transformed data

- Seroprotection rate for anti-HBsAg is defined as the percentage of subjects with antibody titers greater than or equal to an established cut-off (anti-HBsAg ≥ 10 mIU/mL).
- A subject seropositive for anti-CS antibody is a subject whose antibody titer is greater than or equal to the cut-off value (anti-CS ≥ 0.5 EU/mL).
- The Geometric Mean Titers (GMTs) calculations are performed by taking the anti-log of the mean of the log10 titer transformations. Antibody titers below the cut-off of the assay will be given an arbitrary value of half the cut-off for the purpose of GMT calculation.

For a given subject and a given immunogenicity measurement, missing or non-evaluable measurements will not be replaced. Therefore, an analysis will exclude subjects with missing or non-evaluable measurements.

10.8. Final analyses

The final analysis will be conducted on data collected until Day 90 (30 days post Dose 3). The study will continue in a single blind manner for additional safety surveillance and immunogenicity assessment. This additional information will be appended to the study report.

10.8.1. Analyses of safety

10.8.1.1. Primary endpoint

For the safety primary endpoint, the occurrence of SAEs will be evaluated on the Total Vaccinated Cohort. The proportion of subjects with an SAE, classified by the MedDRA preferred term level, reported from study start until study conclusion (end of the double-blind phase) will be tabulated with exact 95% CI. Comparisons between groups will be done using Fisher's Exact test for each preferred term.

For the primary immunogenicity endpoint, for anti-CS GMTs 1 month post Dose 3, the assessment of non-inferiority of the RTS,S/AS01E group versus the RTS,S/AS02D group will be performed by comparing the upper limit of the 95% CI on the ratio of GMTs (RTS,S/AS02D / RTS,S/AS01E group) with a pre-defined non-inferiority limit, set at 3. The analysis will be performed on the ATP cohort for immunogenicity. If more than 5% of subjects are excluded from the ATP cohort for immunogenicity, a second analysis will be performed on the total vaccinated cohort.

10.8.1.2. Secondary safety endpoints

Safety will be analyzed on the Total Cohort.

The percentage of subjects with at least one local adverse event (solicited and unsolicited), with at least one general adverse event (solicited and unsolicited) and with any adverse event during the solicited follow-up period will be tabulated with exact 95% CI after each vaccine dose and overall. The percentage of doses followed by at least one local adverse event (solicited and unsolicited), by at least one general adverse event (solicited and unsolicited) and by any adverse event will be tabulated, overall vaccination course, with exact 95% CI. Similar tables will be generated for Grade 3 events, the relationship of the event to vaccination.

The percentage of subjects reporting each individual solicited local and general adverse event during the solicited follow-up period will be tabulated with exact 95% CI. The percentage of doses followed by each individual solicited local and general adverse event will be tabulated, overall vaccination course, with exact 95% CI. Similar tables will be generated for Grade 3 events, the relationship of the event to vaccination and for fever, temperature (in 0.5°C increments).

The proportion of subjects reporting an AE (unsolicited) 30 days post each vaccination, classified by the MedDRA preferred term level, reported from study start until the end of the double-blind phase (visit 6) will be tabulated with exact 95% CI.

Biochemistry (ALT, bilirubin, creatinine) and hematology (hemoglobin, WBC, platelets) values that are outside of the reference ranges will be described for Clinic Visit 6 (end of double-blind phase). Frequency distribution of results by toxicity grades will be tabulated by group.

10.8.2. Analyses of immunogenicity

The primary analysis will be based on the ATP cohort for analysis of immunogenicity. If the percent of enrolled subjects excluded from this ATP cohort is more than 5%, a second analysis based on the Total Vaccinated cohort will be performed to complement the ATP analysis.

For the secondary immunogenicity endpoint, for anti-HBs GMTs 1 month post Dose 3, the assessment of non-inferiority of the RTS,S/AS01E group versus the RTS,S/AS02D group will be performed by comparing the upper limit of the 95% CI on the ratio of GMTs (RTS,S/AS02D / RTS,S/AS01E group) with a pre-defined non-inferiority limit, set at 3. The analysis will be performed on the ATP cohort for immunogenicity. If more than 5% of subjects are excluded from the ATP cohort for immunogenicity, a second analysis will be performed on the total vaccinated cohort.

10.8.2.1. Anti-CS antibodies

The percentage of subjects with sero-positive levels of anti-CS (proportion of subjects with anti-CS antibody titers greater than or equal to 0.5 EU/mL) with 95% CI will be determined at each blood sampling time point. Antibody titers will be summarized by GMT with 95% CI at all time points at which serological samples are taken. Antibody titers after Dose 3 will also be investigated using reverse cumulative curves.

10.8.2.2. Anti-HBs antibodies

The percentage of subjects with sero-protective levels of anti-HBs (proportion of subjects with anti-HBs antibody titers greater than or equal to 10 mIU/mL) with 95% CI will be determined at each blood sampling time point. Antibody titers will be summarized by GMT with 95% CI at all time points at which serological samples are taken. Antibody titers after Dose 3 will also be investigated using reverse cumulative curves.

10.8.3. Exploratory analysis

The percentage of CS-specific CD4+, CD8+ cells and B-cell memory will be summarized for each group at each timepoint (descriptive statistics).

10.9. Annex analysis at study conclusion

10.9.1. Analyses of safety

The analysis of the occurrence of SAEs will be performed on the Total Vaccinated Cohort. The proportion of subjects with an SAE, classified by the MedDRA preferred term level, reported from study start until study conclusion (end of the single-blind phase) will be tabulated with exact 95% CI. Comparisons between groups will be done using Fisher's Exact test for each preferred term.

Serious adverse events and withdrawal due to adverse event(s) will be described in detail.

Biochemistry (ALT, creatinine) and hematology (hemoglobin, WBC, platelets) values that are outside of the reference ranges will be described for Clinic Visit 9 (end of single-blind phase).

10.9.2. Analyses of immunogenicity

The primary analysis will be based on the ATP cohort for analysis of immunogenicity. If the percent of enrolled subjects excluded from this ATP cohort is more than 5%, a second analysis based on the Total Vaccinated cohort will be performed to complement the ATP analysis.

10.9.2.1. Anti-HBs antibodies

The seroprotective level for anti-HBs is ≥ 10 mIU/mL; The percentage of subjects with protective levels of anti-HBs (≥ 10 mIU/mL) with 95% confidence interval (95% CI) will be determined at Month 14 (Clinic Visit 7). Antibody titers will be summarized by GMT with 95% CI at Month 14 (Clinic Visit 7). Antibody titers after Dose 3 will also be investigated using reverse cumulative curves.

10.9.2.2. Anti-CS antibodies

The percentage of subjects with sero-positive levels of anti-CS (proportion of subjects with anti-CS antibody titers greater than or equal to 0.5 EU/mL) with 95% CI will be determined at Clinic Visit 7. Antibody titers will be summarized by GMT with 95% CI. Antibody titers after Dose 3 will also be investigated using reverse cumulative curves.

10.9.2.3. Exploratory analysis

The percentage of CS-specific CD4+, CD8+ and B-cell memory will be summarized for each group at each timepoint (descriptive statistics).

The percentage of subjects with sero-positive levels of anti-CS (proportion of subjects with anti-CS antibody titers greater than or equal to 0.5 EU/mL) with 95% CI according to HBV immunization status at screening will be determined at Clinic Visit 7. Antibody titers will be summarized by GMT with 95% CI. Antibody titers after Dose 3 according to HBV immunization status will also be investigated using reverse cumulative curves.

10.10. Planned interim analysis

No interim analysis is planned in this study. Final analysis will be carried out at Day 90.

11. ADMINISTRATIVE MATTERS

To comply with Good Clinical Practice important administrative obligations relating to investigator responsibilities, monitoring, archiving data, audits, confidentiality and publications must be fulfilled. See Appendix B for details.

12. REFERENCES

Alloueche A, Milligan P, Conway DJ, Pinder M, Bojang K, Doherty T et al. Protective efficacy of the RTS,S/AS02 *Plasmodium falciparum* malaria vaccine is not strain specific. *American Journal of Tropical Medicine and Hygiene.* 2003; 68: 97-101.

Alloueche A, Bailey W, Barton S, Bwika J, Chimpeni P, et al. Comparison of chlorproguanil-dapsone with sulfadoxine-pyrimethamine for the treatment of uncomplicated falciparum malaria in young African children: double-blind randomised controlled trial. *Lancet*. 2004; 363: 1843-8.

Alonso PL, Sacarlal J, Aponte JJ, Leach A, Macete E et al. Efficacy of the RTS,S/AS02A vaccine against *Plasmodium falciparum* infection and disease in young African children: randomised controlled trial. *The Lancet*. 2004;364:1411-1420.

Alonso PL, Sacarlal J, Aponte JJ, Leach A, Macete E et al. Duration of protection with RTS,S/AS02A malaria vaccine in prevention of *Plasmodium falciparum* disease in Mozambican children: single-blind extended follow-up of a randomised controlled trial. *The Lancet.* 2005; *in press* http://www.sciencedirect.com/science?_ob=MImg&_imagekey=B6T1B-4HK04M5-1-1&_cdi=4886&_user=1001915&_orig=browse&_coverDate=11%2F15%2F2005&_sk=999999999&view=c&wchp=dGLbVzb-zSkWz&md5=b65c581bd880daa749f0f458977c6b5b&ie=/sdarticle.pdf. Accessed 22 November 2005.

Bojang KA, Milligan PJM, Pinder M, Vigneron L, Alloueche A, Kester KE et al. Efficacy of RTS,S/AS02 Malaria Vaccine against *Plasmodium falciparum* Infection in Semi-Immune Adult Men in the Gambia: A Randomized Trial. *The Lancet*. 2001;358: 1927-34.

Bojang KA, Olodude F, Pinder M, Ofori-Anyinam O, Vigneron L et al. Safety and immunogenicty of RTS,S/AS02A candidate malaria vaccine in Gambian children. *Vaccine*. 2005; 23: 4148–4157.

Bonhoeffer J, Menkes J, Gold, MS, de Souza-Brito G, Fisher MC et al. Generalized convulsive seizure as an adverse event following immunization: case definition and guidelines for data collection, analysis and presentation. *Vaccine*. 2004; 22: 557-562.

Borrmann S, Binder RK, Adegnika AA, Missinou MA, Issifou S, Ramharter M et al. Reassessment of the resistance of *Plasmodium falciparum* to chloroquine in Gabon: implications for the validity of tests in vitro vs. in vivo. *Trans R Soc Trop Med Hyg.* 2002; 96: 660-3.

Breman JG, Egan A, Keusch GT. Introduction and Summary. The Intolerable Burden of Malaria: A New Look at the Numbers. *Am J Trop Med Hyg*. 2001;64 (1, 2)S: iv-vii.

Breman, JG. The Ears of the Hippopotamus: Manifestations, Determinants and Estimates of the Malaria Burden. *Am J Trop Med Hyg*. 2001; 64:1-11.

Debat Zoguereh D, Milleliri JM, Lemardeley P, Boumah AJ. Assessment of information status, behaviours and perception of risk of AIDS in rural Gabon. *Trop Doct.* 2004; 34: 157-9.

Doherty JF, Pinder M, Tornieporth N, Carton C, Vigneron L, Milligan P et al. A Phase I Safety and Immunogenicity Trial with the Candidate Malaria Vaccine RTS,S.SBAS02 in Semi-Immune Adults in The Gambia. *J Am Soc Trop Med Hyg*. 1999; 61 (6): 865-68.

Epstein JE, Charoenvit Y, Kester KE, Wang R, Newcomer R, Fitzpatrick S et al. Safety, tolerability, and antibody responses in humans after sequential immunization with a PfCSP DNA vaccine followed by the recombinant protein vaccine RTS,S/AS02A. *Vaccine.* 2004; 22: 1592–1603.

Gallup JL, Sachs JD. The Economic Burden of Malaria. *Am J Trop Med Hyg*. 2001; 64: 85-96.

GSK data on file: Investigator Brochure GlaxoSmithKline Biologicals' Candidate Vaccines Against *Plasmodium falciparum* Malaria (RTS,S/AS02A, RTS,S/AS02D and RTS,S/AS01B Malaria Vaccines). 23 May 2005. Data on File at GlaxoSmithKline Biologicals.

GSK data on file: Safety, Immunogenicity and Efficacy of Three Adjuvant Preparations of a *Plasmodium falciparum* Vaccine (RTS,S) Which Utilizes the Hepatitis B Surface Antigen (HBsAg) as a Carrier for the Malaria Circumsporozoite (CS) Antigen. Report WRMAL-003. 24 August 1996. Data on File at GlaxoSmithKline Biologicals.

GSK data on file: Safety, Immunogenicity, and Preliminary Efficacy of a Lyophilized Formulation of SmithKline Beecham Biologicals' Candidate Malaria Vaccine RTS,S. Report WRMAL-005. 19 August 1999. Data on File at GlaxoSmithKline Biologicals.

GSK data on file: Dosage and Schedule Optimization of RTS,S Malaria Vaccine. Report WRMAL-004. 30 April 1998. Data on File at GlaxoSmithKline Biologicals.

GSK data on file: Phase IIb controlled randomized double-blind study to evaluate the efficacy, safety and immunogenicity of SmithKline Beecham Biologicals' candidate RTS,S malaria vaccine in semi-immune adult males in The Gambia. Protocol 257049/005 (Malaria-005) 12 December 1997. Data on File at GlaxoSmithKline Biologicals.

GSK data on file: Phase I/IIa safety, immunogenicity, and preliminary efficacy of two short-course schedules of SmithKline Beecham Biologicals' candidate malaria vaccine RTS,S in non-immune adult volunteers. Report 257049/012 (Malaria-012) 01 March 2002. Data on File at GlaxoSmithKline Biologicals.

GSK data on file: A Phase I Double Blind, Randomized, Controlled, Staggered Study to Evaluate the Safety, Reactogenicity and Immunogenicity of 1/5, 1/2 and Full Doses of GlaxoSmithKline Biologicals' RTS,S/AS02 Candidate Malaria Vaccine, Administered IM According to a 0, 1, 3-month Vaccination Schedule in Semi-Immune Children aged 6 to 11 Years in The Gambia, a Malaria Endemic Region. Report 257049/015 (Malaria-015) 14 October 2003. Data on File at GlaxoSmithKline Biologicals.

GSK data on file: Phase II b controlled randomized double-blind study to evaluate the efficacy, safety and immunogenicity of GlaxoSmithKline Biologicals' candidate RTS,S malaria vaccine in semi-immune adult males in The Gambia. Report 257049/016, 257049/017 & 257049/018 (Malaria-016, -017 & -018) 12 January 2005. Data on File at GlaxoSmithKline Biologicals.

GSK data on file: A Phase I Double Blind, Randomized, Controlled, Staggered Study to Evaluate the Safety, Reactogenicity and Immunogenicity of 1/5, 1/2 and Full Doses of GlaxoSmithKline Biologicals' RTS,S/AS02 Candidate Malaria Vaccine, Administered IM According to a 0, 1, 3-month Vaccination Schedule in Semi-Immune Children aged 1 to 5 Years in The Gambia, a Malaria Endemic Region. Report 257049/020 (Malaria-020) 27 February 2003. Data on File at GlaxoSmithKline Biologicals.

GSK data on file: A Phase I Double Blind, Randomized, Controlled, Staggered Study to Evaluate the Safety, Reactogenicity and Immunogenicity of GlaxoSmithKline Biologicals' RTS,S/AS02 Candidate Malaria Vaccine, Administered IM According to a 0, 1, 2-month Vaccination Schedule in Children aged 1 through 4 years in Mozambique, a Malaria Endemic Region. Report 257049/025 (Malaria-025) 10 October 2003. Data on File at GlaxoSmithKline Biologicals.

GSK data on file: A double-blind randomised controlled phase IIb study to evaluate the safety, immunogenicity and efficacy of GlaxoSmithKline Biologicals' candidate malaria vaccine RTS,S/AS02A, administered IM according to a 0, 1 and 2 month vaccination schedule in toddlers and children aged 1 to 4 years in a malaria-endemic region of Mozambique. Report 257049/026 (Malaria-026) 19 November 2004. Data on File at GlaxoSmithKline Biologicals.

GSK data on file: Double-blind, randomized phase I/IIa human challenge study, to evaluate the safety, reactogenicity, immunogenicity, and preliminary efficacy after primary sporozoite challenge and rechallenge, of GSK Biologicals' candidate malaria vaccines containing the antigen RTS,S adjuvanted with either AS02A or AS01B and administered intramuscularly at months 0, 1, 2 in healthy malaria-naive volunteers aged 18 - 45 years. Protocol 257049/027 (Malaria-027) 26 August 2005. Data on File at GlaxoSmithKline Biologicals.

GSK data on file: A Phase I/II randomized double-blind bridging study to evaluate the safety and immunogenicity of GlaxoSmithKline Biologicals' candidate vaccines RTS,S/AS02D (0.5 mL dose) and RTS,S/AS02A (0.25 mL dose) administered IM according to a 0, 1, 2 month vaccination schedule in children aged 3 to 5 years living in a malaria-endemic region of Mozambique. Report 257049/034 (Malaria-034) 06 December 2004. Data on File at GlaxoSmithKline Biologicals.

Kester KE, McKinney DA, Tornieporth N, Ockenhouse CF, Heppner DG, et al. Efficacy of recombinant circumsporozoite protein vaccine regimens against experimental *Plasmodium falciparum* malaria. *Journal of Infectious Diseases*. 2001; 15:183(4):640-7.

Lalvani A, Moris P, Voss G, Kester KE, Brookes R, Koutsoukos M. Potent induction of focused Th-1 type cellular and humoral immune responses by RTS,S/SBAS2

recombinant *P. falciparum* malaria vaccine. *Journal of Infectious Diseases*. 1999; 180:1656-64.

Lell B, May J, Schmidt-Ott RJ, Lehman LG, Luckner D, Greve B, Matousek P, Schmid D, Herbich K, Mockenhaupt FP, Meyer CG, Bienzle U, Kremsner PG. The role of red blood cell polymorphisms in resistance and susceptibility to malaria. Clin Infect Dis. 1999; 28:794-9.

Smith PG, Morrow RH. *Methods for Field Trials of Interventions against Tropical Diseases: A Toolbox*. Oxford University Press 1991: 152-155; 168-175.

Snow RW, Schellenberg, JR, Peshu, D, Forster, CR, Newton, PA et al. Periodicity and space-time clustering of severe childhood malaria on the coast of Kenya. *Trans. R. Soc. Trop. Med. Hyg*. 1993: 87 (4): 386-390.

Snow RW, de Azevedo Bastos, I, Lowe, EW, Kabiru, CG, Nevill, CG et al. Severe childhood malaria in two areas of markedly different falciparum transmission in east Africa. *Acta Trop*. 1994: 57 (4): 289-300, 1994.

Stoute JA, Slaoui M, Heppner DG, Momin P, Kester KE, Desmons P et al. A Preliminary Evaluation of a Recombinant Circumsporozoite Protein Vaccine against *Plasmodium falciparum* Malaria. *N Eng J Med* 1997; 336: 86-91

Stoute JA, Kester KE, Krzych U, Wellde BT, Hall T, White K et al. Long Term Efficacy and Immune Responses Following Immunization with the RTS,S Malaria Vaccine. *Journal of Infectious Diseases*. 1998; 178: 1139-44.

Sun P, Schwenk R, White K, Stoute JA, Cohen J, Ballou WR et al. Protective immunity induced with malaria vaccine, RTS,S, is linked to *Plasmodium falciparum* circumsporozoite protein-specific CD4(+) and CD8(+) T cells producing IFN-gamma. *Journal of Immunology*. 2003; 171 (12): 6961-7.

Sylla EH, Kun JF, Kremsner PG. Mosquito distribution and entomological inoculation rates in three malaria-endemic areas in Gabon. *Trans R Soc Trop Med Hyg*. 2000; 94:652-6.

Sylla EH, Lell B, Kun JF, Kremsner PG. *Plasmodium falciparum* transmission intensity and infection rates in children in Gabon. *Parasitol Res*. 2001; 87:530-3.

United Nations Children's Fund. At a Glance: Gabon; Statistics. http://www.unicef.org/infobycountry/gabon_statistics.html. Accessed 28 November 2005

Vryheid RE, Kane MA, Muller N, Schatz GC, Bezabeh S. Infant and Adolescent Hepatitis B Immunization up to 1999: A Global Overview. *Vaccine* 2001;19: 1026-1037.

Wildling E, Winkler S, Kremsner PG, Brandts C, Jenne L, Wernsdorfer WH. Malaria epidemiology in the province of Moyen Ogooue, Gabon. *Trop Med Parasitol.* 1995; 46:77-82.

World Health Organization. Hepatitis B Fact Sheet WHO/204. http://www.who.int/mediacentre/factsheets/fs204/en/index.html. Accessed 29 November 2005.

World Health Organization. Procurement of Artemether-Lumefantrine (Coartem®) through WHO. http://mosquito.who.int/cmc_upload/0/000/015/789/CoA_website5.pdf. Accessed 29 November 2005.

APPENDIX A WORLD MEDICAL ASSOCIATION DECLARATION OF HELSINKI

Recommendations guiding physicians in biomedical research involving human subjects

Adopted by the 18th World Medical Assembly
Helsinki, Finland, June 1964
and amended by the
29th World Medical Assembly
Tokyo, Japan, October 1975
35th World Medical Assembly
Venice, Italy, October 1983
41st World Medical Assembly
Hong Kong, September 1989
and the
48th General Assembly
Somerset West, Republic of South Africa, October 1996

INTRODUCTION

It is the mission of the physician to safeguard the health of the people. His or her knowledge and conscience are dedicated to the fulfillment of this mission.

The Declaration of Geneva of the World Medical Association binds the physician with the words, "The health of my patient will be my first consideration," and the International Code of Medical Ethics declares that, "A physician shall act only in the patient's interest when providing medical care which might have the effect of weakening the physical and mental condition of the patient."

The purpose of biomedical research involving human subjects must be to improve diagnostic, therapeutic and prophylactic procedures and the understanding of the etiology and pathogenesis of disease.

In current medical practice most diagnostic, therapeutic or prophylactic procedures involve hazards. This applies especially to biomedical research.

Medical progress is based on research which ultimately must rest in part on experimentation involving human subjects.

In the field of biomedical research a fundamental distinction must be recognized between medical research in which the aim is essentially diagnostic or therapeutic for a patient, and medical research, the essential object of which is purely scientific and without implying direct diagnostic or therapeutic value to the person subjected to the research.

Special caution must be exercised in the conduct of research which may affect the environment, and the welfare of animals used for research must be respected.

Because it is essential that the results of laboratory experiments be applied to human beings to further scientific knowledge and to help suffering humanity, the World Medical Association has prepared the following recommendations as a guide to every physician in biomedical research involving human subjects. They should be kept under review in the future. It must be stressed that the standards as drafted are only a guide to physicians all over the world. Physicians are not relieved from criminal, civil and ethical responsibilities under the laws of their own countries.

I. BASIC PRINCIPLES

1. Biomedical research involving human subjects must conform to generally accepted scientific principles and should be based on adequately performed laboratory and animal experimentation and on a thorough knowledge of the scientific literature.
2. The design and performance of each experimental procedure involving human subjects should be clearly formulated in an experimental protocol which should be transmitted for consideration, comment and guidance to a specially appointed committee independent of the investigator and the sponsor provided that this independent committee is in conformity with the laws and regulations of the country in which the research experiment is performed.
3. Biomedical research involving human subjects should be conducted only by scientifically qualified persons and under the supervision of a clinically competent medical person. The responsibility for the human subject must always rest with a medically qualified person and never rest on the subject of research, even though the subject has given his or her consent.
4. Biomedical research involving human subjects cannot legitimately be carried out unless the importance of the objective is in proportion to the inherent risk to the subject.
5. Every biomedical research project involving human subjects should be preceded by careful assessment of predictable risks in comparison with foreseeable benefits to the subject or to others. Concern for the interests of the subject must always prevail over the interests of science and society.
6. The right of the research subject to safeguard his or her integrity must always be respected. Every precaution should be taken to respect the privacy of the subject and to minimize the impact of the study on the subject's physical and mental integrity and on the personality of the subject.
7. Physicians should abstain from engaging in research projects involving human subjects unless they are satisfied that the hazards involved are believed to be predictable. Physicians should cease any investigation if the hazards are found to outweigh the potential benefits.
8. In publication of the results of his or her research, the physician is obliged to preserve the accuracy of the results. Reports of experimentation not in accordance with the principles laid down in this Declaration should not be accepted for publication.
9. In any research on human beings, each potential subject must be adequately informed of the aims, methods, anticipated benefits and potential hazards of the

study and the discomfort it may entail. He or she should be informed that he or she is at liberty to abstain from participation in the study and that he or she is free to withdraw his or her consent to participation at any time. The physician should then obtain the subject's freely-given informed consent, preferably in writing.

10. When obtaining informed consent for the research project the physician should be particularly cautious if the subject is in a dependent relationship to him or her or may consent under duress. In that case the informed consent should be obtained by a physician who is not engaged in the investigation and who is completely independent of this official relationship.
11. In case of legal incompetence, informed consent should be obtained from the legal guardian in accordance with national legislation. Where physical or mental incapacity makes it impossible to obtain informed consent, or when the subject is a minor, permission from the responsible relative replaces that of the subject in accordance with national legislation.
    Whenever the minor child is in fact able to give a consent, the minor's consent must be obtained in addition to the consent of the minor's legal guardian.
12. The research protocol should always contain a statement of the ethical considerations involved and should indicate that the principles enunciated in the present Declaration are complied with.

II. MEDICAL RESEARCH COMBINED WITH PROFESSIONAL CARE

(Clinical research)

1. In the treatment of the sick person, the physician must be free to use a new diagnostic and therapeutic measure, if in his or her judgment it offers hope of saving life, re-establishing health or alleviating suffering.
2. The potential benefits, hazards and discomfort of a new method should be weighed against the advantages of the best current diagnostic and therapeutic methods.
3. In any medical study, every patient—including those of a control group, if any—should be assured of the best proven diagnostic and therapeutic method. This does not exclude the use of inert placebo in studies where no proven diagnostic or therapeutic method exists.
4. The refusal of the patient to participate in a study must never interfere with the physician–patient relationship.
5. If the physician considers it essential not to obtain informed consent, the specific reasons for this proposal should be stated in the experimental protocol for transmission to the independent committee (I, 2).
6. The Physician can combine medical research with professional care, the objective being the acquisition of new medical knowledge, only to the extent that medical research is justified by its potential diagnostic or therapeutic value for the patient.

III. NON-THERAPEUTIC BIOMEDICAL RESEARCH INVOLVING HUMAN SUBJECTS

(Non-clinical biomedical research)

1. In the purely scientific application of medical research carried out on a human being, it is the duty of the physician to remain the protector of the life and health of that person on whom biomedical research is being carried out.
2. The subjects should be volunteers—either healthy persons or patients for whom the experimental design is not related to the patient's illness.
3. The investigator or the investigating team should discontinue the research if in his/her or their judgment it may, if continued, be harmful to the individual.

In research on man, the interest of science and society should never take precedence over considerations related to the well being of the subject.

APPENDIX B ADMINISTRATIVE MATTERS

I. Responsibilities of the Investigator

- To ensure that he/she has sufficient time to conduct and complete the study and has adequate staff and appropriate facilities and equipment which are available for the duration of the study and to ensure that other studies do not divert essential subjects or facilities away from the study at hand.
- To submit an up-to-date curriculum vitae and other credentials (e.g., medical license number in the United States) to GSK Biologicals and—where required—to relevant authorities.
- To acquire the normal ranges for laboratory tests performed locally and, if required by local regulations, obtain the Laboratory License or Certification.
- To ensure that no clinical samples (including serum samples) are retained on site or elsewhere without the approval of GSK Biologicals and the express written informed consent of the subject and/or the subject's legally authorized representative.
- To perform no other biological assays at the investigator site except those described in the protocol or its amendment(s).
- To prepare and maintain adequate case histories designed to record observations and other data pertinent to the study.
- To conduct the study in compliance with the protocol and appendices.
- To co-operate with a representative of GSK Biologicals in the monitoring process of the study and in resolution of queries about the data.

II. Protocol Amendments and Administrative changes

- No changes to the study protocol will be allowed unless discussed in detail with the GSK Biologicals' Clinical Development Manager/Medical Monitor and filed as an amendment/administrative change to this protocol.
- Any amendment/administrative change to the protocol will be adhered to by the participating center and will apply to all subjects. Written IRB/IEC approval of protocol amendments is required prior to implementation; administrative changes are submitted to IRBs/IECs for information only.
- Any amendment/administrative change to the protocol will be adhered to by the participating center and will apply to all subjects. Written IRB/IEC approval of protocol amendments/administrative changes is required prior to implementation.

III. Sponsor's Termination of Study

GSK Biologicals reserves the right to temporarily suspend or prematurely discontinue this study either at a single site or at all sites at any time for reasons including, but not limited to, safety or ethical issues or severe non-compliance. Reasons for suspension or early termination will be documented in the study file at GSK Biologicals.

If GSK Biologicals determines that suspension or early termination is needed, GSK Biologicals will discuss this with the Investigator (including the reasons for taking such action). When feasible, GSK Biologicals will provide advance notification to the investigator of the impending action prior to it taking effect.

GSK Biologicals will promptly inform, via written communication, all investigators and/or institutions conducting the study, if the study is suspended or terminated for safety reasons, and will also inform the regulatory authorities of the suspension or termination of the study and the reason(s) for the action. If required by applicable regulations, the investigator must inform the IEC/IRB promptly and provide the reason for the suspension or termination.

If the study is prematurely discontinued, all study data must be returned to GSK. In addition, arrangements will be made for all unused investigational product(s) in accordance with the applicable GSK procedures for the study. Financial compensation to investigators and/or institutions will be in accordance with the agreement established between the investigator and/or institutions and GSK.

IV. Case Report Form Instructions

Prior to screening the first potential participant, the investigator will provide the Site Monitor with a list (Site Staff Signature Sheet) showing the signature and hand-written initials of all individuals authorized to make or change entries on CRFs (already defined). If the authorized individuals should change during the study, the investigator is to inform GSK Biologicals of the specific change(s).

CRFs (and subject diary cards, if applicable), will be supplied by GSK Biologicals for recording all data. It is the responsibility of the investigator or co-investigator to ensure that CRFs (and subject diary cards) are legible and completely filled in with a black ink fountain or ballpoint pen.

Errors must be corrected by drawing a single line through the incorrect entry and writing in the new value/data positioned as close to the original as possible. The correction must then be initialed, dated and justified, where necessary, by the authorized individual making the change. The original entry must not be obliterated, overwritten or erased when a correction is made.

When a subject completes a visit, it is anticipated that relevant sections of the CRF will be completed by the investigator (or designated staff as documented in the Site Staff Signature Sheet) as soon as possible after the last data becoming available. Similarly, when a subject completes a study, it is anticipated that all relevant CRF pages will be completed promptly after the last data becoming available. This also applies to forms for potential study participants who were screened but not randomized to a study group.

As soon as the subject has completed/withdrawn from the study and the CRF is completed, the principal investigator or designated physicians under his/her supervision will sign the study conclusion pages of the CRF to confirm that they have reviewed the data and that the data are complete and accurate. In all cases the investigator remains accountable for the study data collected.

An original (top copy) CRF or log sheets must be submitted for all subjects who have undergone protocol specific procedures, whether or not the subject completed the study.

While completed CRFs are reviewed by a GSK Biologicals professional monitor at the study site, errors detected by subsequent in-house CRF review may necessitate clarification or correction of errors with documentation and approval by the investigator or appropriately qualified staff as documented on the Site Staff Signature Sheet. In all cases, the investigator remains accountable for the study data. Wherever possible the investigator should assist in the clarification or correction of errors detected after study finalization promptly after being brought to the attention of the investigator (preferably within 48 hours).

Any questions or comments related to the CRF should be directed to the assigned Site Monitor.

V. Monitoring by GSK Biologicals

Monitoring visits by a professional representative of the sponsor will be scheduled to take place as close as possible to entry of the first subject, during the study at appropriate intervals and after the last subject has completed the study. It is anticipated that monitoring visits will occur at a frequency defined before study start.

These visits are for the purpose of confirming that GSK Biologicals' sponsored studies are being conducted in compliance with the relevant Good Clinical Practice regulations/ guidelines, verifying adherence to the protocol and the completeness and accuracy of data entered on the CRF pages and Vaccine Inventory Forms. The monitor will verify CRF entries by comparing them with the source data/documents that will be made available by the investigator for this purpose. Data to be recorded directly into the CRF pages screens will be specified in writing preferably in the source documentation agreement form that is contained in both the monitor's and investigator's study file. The investigator must ensure provision of reasonable time, space and adequate qualified personnel for monitoring visits.

VI. Archiving of Data

Following closure of the study, the investigator must maintain all site study records in a safe and secure location. The records must be maintained to allow easy and timely retrieval, when needed (e.g., audit or inspection), and, whenever feasible, to allow any subsequent review of data in conjunction with assessment of the facility, supporting systems, and staff. Where permitted by applicable laws/regulations or institutional policy, some or all of these records can be maintained in a validated format other than hard copy (e.g., microfiche, scanned, electronic for studies with an eCRF, for example); however, caution needs to be exercised before such action is taken. The investigator must assure that all reproductions are legible and are a true and accurate copy of the original, and meet accessibility and retrieval standards, including re-generating a hard copy, if required. Furthermore, the investigator must ensure there is an acceptable back-up of these reproductions and that an acceptable quality control process exists for making these reproductions.

GSK will inform the investigator/ institution of the time period for retaining these records to comply with all applicable regulatory requirements. However, the investigator/ institution should seek the written approval of the sponsor before proceeding with the disposal of these records. The minimum retention time will meet the strictest standard applicable to that site for the study, as dictated by ICH GCP E6 Section 4.9, any institutional requirements or applicable laws or regulations, or GSK standards/procedures; otherwise, the minimum retention period will default to 15 years.

The investigator/ institution must notify GSK of any changes in the archival arrangements, including, but not limited to, the following: archival at an off-site facility, transfer of ownership of the records in the event the investigator leaves the site.

VII. Audits

For the purpose of compliance with Good Clinical Practice and Regulatory Agency Guidelines it may be necessary for GSK Biologicals or a Drug Regulatory Agency to conduct a site audit. This may occur at any time from start to after conclusion of the study.

When an investigator signs the protocol, he agrees to permit drug regulatory agencies and GSK Biologicals audits, providing direct access to source data/ documents. Furthermore, if an investigator refuses an inspection, his data will not be accepted in support of a New Drug Registration and/or Application, Biologics Licensing Application.

GSK Biologicals has a substantial investment in clinical studies. Having the highest quality data and studies are essential aspects of vaccine development. GSK Biologicals has a Regulatory Compliance staff who audit investigational sites. Regulatory Compliance assesses the quality of data with regard to accuracy, adequacy and consistency. In addition, Regulatory Compliance assures that GSK Biologicals sponsored studies are in accordance with GCP and that relevant regulations/guidelines are being followed.

To accomplish these functions, Regulatory Compliance selects investigational sites to audit. These audits usually take 1 to 2 days. The GSK Biologicals' audits entail review of source documents supporting the adequacy and accuracy of CRFs, review of documentation required to be maintained, and checks on vaccine accountability. The GSK Biologicals' audit therefore helps prepare an investigator for a possible regulatory agency inspection as well as assuring GSK Biologicals of the validity of the database across investigational sites.

The Inspector will be especially interested in the following items:

- Log of visits from the sponsor's representatives
- IRB/IEC approval
- Vaccine accountability
- Approved study protocol and amendments
- Informed consent of the subjects (written consent [or witnessed oral if applicable])

- Medical records and other source documents supportive of CRF data
- Reports to the IRB/IEC and the sponsor
- Record retention

GSK Biologicals will gladly help investigators prepare for an inspection.

VIII. Ownership, Confidentiality and Publication

Ownership:

All information provided by GSK and all data and information generated by the site as part of the study (other than a subject's medical records) are the sole property of GSK.

All rights, title, and interests in any inventions, know-how or other intellectual or industrial property rights which are conceived or reduced to practice by site staff during the course of or as a result of the study are the sole property of GSK, and are hereby assigned to GSK.

If a written contract for the conduct of the study which includes ownership provisions inconsistent with this statement is executed between GSK and the study site, that contract's ownership provisions shall apply rather than this statement.

Confidentiality:

All information provided by GSK and all data and information generated by the site as part of the study (other than a subject's medical records) will be kept confidential by the investigator and other site staff. This information and data will not be used by the investigator or other site personnel for any purpose other than conducting the study. These restrictions do not apply to: (1) information which becomes publicly available through no fault of the investigator or site staff; (2) information which it is necessary to disclose in confidence to an IEC or IRB solely for the evaluation of the study; (3) information which it is necessary to disclose in order to provide appropriate medical care to a study subject; or (4) study results which may be published as described in the next paragraph. If a written contract for the conduct of the study which includes confidentiality provisions inconsistent with this statement is executed, that contract's confidentiality provisions shall apply rather than this statement.

Publication:

For multicenter studies, the first publication or disclosure of study results shall be a complete, joint multicenter publication or disclosure coordinated by GSK. Thereafter, any secondary publications will reference the original publication(s).

Prior to submitting for publication, presentation, use for instructional purposes, or otherwise disclosing the study results generated by the site (collectively, a "Publication"), the investigator shall provide GSK and MVI at PATH with a copy of the proposed Publication and allow GSK and MVI at PATH a period of at least thirty (30) days [or, for abstracts, at least five (5) working days] to review the proposed Publication. Proposed

Publications shall not include either GSK confidential information other than the study results or personal data on any subject, such as name or initials.

At GSK's request, the submission or other disclosure of a proposed Publication will be delayed a sufficient time to allow GSK to seek patent or similar protection of any inventions, know-how or other intellectual or industrial property rights disclosed in the proposed Publication.

If a written contract for the conduct of the study, which includes publication provisions inconsistent with this statement is executed, that contract's publication provisions shall apply rather than this statement.

Any publication or presentation shall state the following in an appropriate location: 'Funded in part by PATH's Malaria Vaccine Initiative.

APPENDIX C OVERVIEW OF THE RECRUITMENT PLAN

Parent(s)/guardian(s) of children already taking part in trials at HAS will be approached and asked if they know of children whose parent(s)/guardian(s) might be interested in enrolling them in the trial. This method has been found to be successful in recruiting sufficient numbers of subjects for trials of similar size in the past.

If parent(s)/guardian(s) are interested will be visited and briefed in their home. During the briefing, study staff will explain the problem of malaria to this community, the current strategies for its control, as well as the limitations of these strategies. The needs and the difficulties of developing a vaccine against malaria will be discussed, as well as an outline of the proposed trial, including the rationale, the background data available and the study objectives. Particular attention will be paid to study procedures, immunization and blood collection. In that respect a full discussion on the purpose of blood collection and the associated risks will be carried out. Parent(s)/guardian(s) who express an interest in enrolling their child will be offered a sample ICF for them to review at home.

The site will compile a list of children whose parents/guardians have expressed an interest in enrolling them in the study. Study personnel will seek individual informed consent for each child from the parent(s)/guardian(s) in privacy. Parent(s)/guardian(s) will again be informed about the study objectives and procedures including immunization and blood collection and they will be encouraged to ask and clarify questions about the trial. The parent(s)/guardian(s) understanding of the Informed Consent form will be verified by use of an oral assessment questionnaire. The parent(s)/guardian(s) and the witness will sign the Informed Consent form. Children whose parent(s)/guardian(s) consent for them to enter the study will be screened according to the procedures outlined in Section 5.3.2 of the Study Protocol.

Health care workers will also be involved in giving further information about the study to those who need it and will facilitate the process of identifying potential study participants. Training sessions will be held for health care workers and staff in the study area. These sessions will explain again in detail the study rationale, objectives and procedures.

APPENDIX D HANDLING OF BIOLOGICAL SAMPLES COLLECTED BY THE INVESTIGATOR

Instructions for Handling of Serum Samples

When materials are provided by GSK Biologicals, it is MANDATORY that all clinical samples (including serum samples) will be collected and stored using exclusively those materials in the appropriate manner. The use of other materials could result in the exclusion of the subject from the ATP analysis. The investigator must ensure that his/her personnel and the laboratory(ies) under his/her supervision comply with this requirement. However, when GSK Biologicals does not provide material for collecting and storing clinical samples, then appropriate materials from the investigator's site are to be used.

1. Collection

The whole blood should be collected observing appropriate aseptic conditions. It is recommended that Vacutainer® tubes WITH integrated serum separator (e.g. Becton-Dickinson Vacutainer SST or Corvac® Sherwood Medical) be used to minimize the risk of hemolysis and to avoid blood cell contamination of the serum when transferring to standard serum tubes.

2. Serum separation

These guidelines aim to ensure high quality serum by minimizing the risk of hemolysis, blood cell contamination of the serum or serum adverse cell toxicity at testing.

- For separation of serum using Vacutainer tubes, the instructions provided by the manufacturer should be followed. Siliconized tubes should never be used (cell toxicity). Often the manufacturer's instruction states that the relative centrifugal acceleration known also as "G" must be "between 1000 and 1300 G" with tubes spinning for ten minutes. Error in calculation of centrifuge speed can occur when laboratory personnel confuse "G" acceleration with "RPM" (revolutions per minute). The speed of centrifugation must be calculated using the "G" rate provided in the manufacturer's instructions and the radius of the centrifuge head. After measuring the radius of the centrifuge machine, a speed/acceleration nomograph must be employed to determine the centrifuge speed in "RPM".
- Following separation, the serum should be aseptically transferred to the appropriate standard tubes using a sterile disposable pipette. The serum should be transferred as gently as possible to avoid blood cell contamination.
- The tube should not be overfilled (max. 3/4 of the total volume) to allow room for expansion upon freezing.
- The tube should be identified by the appropriate label provided by GSK Biologicals (see point 3).

3. Labeling

- The standard labels provided by GSK Biologicals should be used to label each serum sample.

- If necessary, any hand-written additions to the labels should be made using indelible ink.
- The label should be attached to the tube as follows (see diagram):
  - first attach the paper part of the label to the tube
  - then wrap the label around the tube so that the transparent, plastic part of the label overlaps with the label text and bar code and shields them.

  This will ensure optimal label attachment.

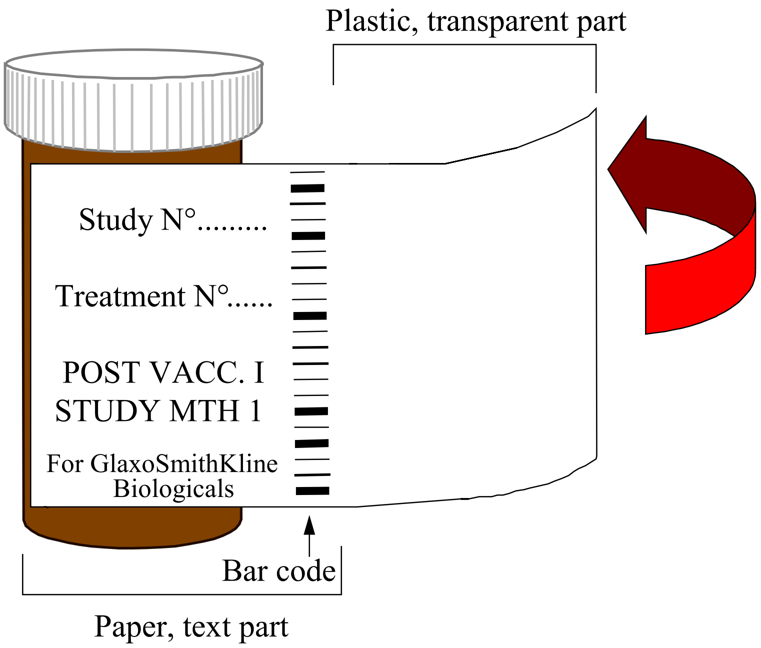

Labels should not be attached to caps.

4. Sorting and storage

- Tubes should be placed in the GSK Biologicals' cardboard boxes in numerical order from left to right, starting from the lower left hand corner, beginning with the pre-vaccination samples series, then with the post-vaccination sample series.
- The tubes of serum should be stored in a vertical position at approximately -20°C (alternatively at approximately -70°/80°C is also acceptable) until shipment to GSK Biologicals. The storage temperature should be checked regularly and documented. Wherever possible, a backup facility for storage of serum samples should be available.
- A standard Serum Listing Form, specifying the samples being shipped for individual subjects at each time point, should be prepared for each shipment. A copy of this list should be retained at the study site, while the original should be sealed in a plastic envelope and shipped with the serum samples.

- Once flight details are known, a standard Specimen Transfer Form must be completed and faxed to GSK Biologicals to the number provided below. A copy of the Specimen Transfer Form must be in the parcel[1]

GLAXOSMITHKLINE BIOLIGICALS
Attention Biospecimen Reception
Clinical Immunology
R & D Department/Building 44
Rue de l'Institut, 89
B-1330 Rixensart – Belgium

Telephone +32-2-656 8949 or +32-2-656 6130
or +32-2-656 8549 or +32-2-656 6108
Fax +32-2-656 6052
E-mail rix.ugbiospecimen-reception@gskbio.com

[1] The Serum Listing Form and the Specimen Transfer Form are standard documents used in GSK Biologicals' clinical trials. These documents are provided by GSK Biologicals' Clinical Trials' monitor at study initiation.

APPENDIX E SHIPMENT OF BIOLOGICAL SAMPLES

Instructions for Shipment of Serum or Plasma Samples

Serum/plasma samples should be sent to GSK Biologicals at regular intervals. The frequency of shipment of samples should be decided upon by the Site Monitor, Central Study Coordinator and the investigator prior to the study start.

Serum/plasma samples should always be sent by air, preferably on a Monday, Tuesday or Wednesday, unless otherwise requested by the sponsor.

Serum/plasma samples must be placed with dry ice (maximum -20°C) in a container complying with International Air Transport Association (IATA) requirements. The completed standard serum listing form should always accompany the shipment.

The container must be clearly identified with the labels provided by GSK Biologicals specifying the shipment address and the storage temperature (-20°C).

The airway bill should contain the instruction for storage of samples at maximum -20°C.

A 'proforma' invoice, stating a value for customs purposes only, should be prepared and attached to the container. This document should contain the instruction for storage of samples at maximum -20°C.

Details of the shipment, including:

* number of samples
* airway bill
* flight number
* flight departure and arrival times

should be sent by fax or email two days before shipment, to:

GLAXOSMITHKLINE BIOLOGICALS,
Attention Biospecimen Reception
Clinical Immunology
R & D Department/Building 44
Rue de l'Institut, 89
B-1330 Rixensart – Belgium

Telephone +32-2-656 8949 or +32-2-656 6130
or +32-2-656 8549 or +32-2-656 6108
Fax +32-2-656 6052
E-mail rix.ugbiospecimen-receiption@gskbio.com

The central study coordinator, Isabelle Ramboer and the local safety monitor, should be informed 2 days before any shipment

Isabelle Ramboer,
Central Study Coordinator,
Rixensart, Belgium
Telephone: +32.2.656.68.20
Fax: +32.2.656.80.44
email: isabelle.ramboer@gskbio.com

~~Jenny Dörnemann,~~ Grégoire Adzoda
Hôpital Albert Schweitzer,
B.P. ~~118, Lambaréné,~~ ***13901, Libreville***
Tel: ~~+241.077.79.225~~ ***+00 241 07 35 83 33***
Fax: +241.581.196
~~email: jennydoernemann@gmx.de~~
gadzoda@yahoo.fr

Administrative Change, 16 February 2006

Instructions for Handling Cells for B and T-Cell-Mediated Immunity Assay

When materials are provided by GSK Biologicals, it is mandatory that all clinical samples be collected and stored using exclusively those materials in the appropriate manner. The use of other materials could result in the exclusion of the subject from analysis. The investigator must ensure that his/her personnel and the laboratory(ies) under his/her supervision comply with this requirement.

1. Collection of whole blood

Collect blood by venipuncture in Terumo tubes with heparin (or equivalent) and record time of collection. The tubes should be kept at room temperature and shipped to a designated clinical site for separation of peripheral blood mononuclear cells (PBMCs). The shipment must be timed to ensure that PBMC separation will be performed within 24 hours. Use well closed Styrofoam boxes of 5 cm thickness for blood samples transport (see current version of GSK Biologicals SOP RD_HCI_001 for guidance).

2. Separation of PBMC

PBMCs will be separated on a density gradient.

3. Freezing of blood cell samples

Blood cells for CMI will aliquoted and frozen at –80°C for 24 hours and further cryopreserved in liquid nitrogen until testing (see GSK Biologicals SOP: RD_HCI_007 for guidance).

4. Labeling of cryotubes for blood cell samples

- If labels are provided by GSK, it is mandatory to use them.
- If necessary, any hand-written additions to the labels should be made using indelible ink.

5. Sorting and storage of blood cell samples

Samples should be stored in liquid nitrogen until shipment to GSK Biologicals, if needed. Wherever possible, a backup facility for storage of samples should be available.

A standard Cryotube Listing Form (see current version of GSK Biologicals SOP RD_HCI_009 for guidance), specifying the samples being shipped for individual subjects at each timepoint, should be prepared for each shipment. A copy of this list should be retained at the study site, while the original should be sealed in a plastic envelope and shipped with the cell samples.

APPENDIX F LABORATORY ASSAYS

Serology testing

Serological responses will be measured principally by evaluating antibody responses to HBs and to CSP repeats (anti R32LR). Serum for antibody determination will be collected at the time points defined in the flowchart in protocol Section 5.10.

Antibody levels against CS will be measured at GSK Biologicals (or a designated laboratory) by standard ELISA methodology using plate adsorbed R32LR antigen with a standard reference antibody as a control according to SOPs from the laboratory. Results will be reported in EU/mL.

Antibody to hepatitis B surface antigen will be measured at GSK Biologicals using a commercially available ELISA immunoassay (AUSAB EIA test kit from Abbott) or equivalent according to the assay instructions. Results will be reported in mIU/mL.

Biochemical and hematological analyses

Hematological and biochemical testing will be done at HAS in Gabon, following laboratory SOPs.

Intracellular Staining for Cytokines

Intracellular fluorescent staining for cytokines (ICS) is a method for detecting defined populations of cells containing cytokine within their cytoplasm. It allows identification of sub-populations (e.g. CD4 T-cells, CD8 T-cells) of cells expressing cytokines as well as the activation status of these cells.

Lymphocytes will be stimulated with CS-derived peptides for 20 hours and brefeldin added to prevent cytokine secretion. The cells will then be fluorescently labeled with antibody directed to a variety of cell surface markers (e.g. CD4 and CD8) to identify specific sub-populations of T-cells. Subsequently, the cells will be fixed with CytoFix (Pharmingen) and treated with a saponin-containing buffer to permeabilize the cell membrane. The cells are then incubated with fluorescently labeled, cytokine-specific or activation marker-specific antibody that forms complexes with the cytokine in the cytoplasm. The cytokine-expressing cells will then be detected by cytofluorometry.

Assessment of frequency of memory B-cells

Assessment of frequency of memory B-cells PBMCs will be cultivated for five days in medium (RPMI supplemented with 10% FCS, penicillin/streptomycin, and glutamine and B-memory maturation agents) to induce maturation of memory B-cells to antibody-secreting cells. After stimulation, cells will be seeded in CS (R32LR)- and anti-human Ig-coated 96-well plates and incubated for one to six hours without mechanical disturbance. Subsequently, plates will be developed and analyzed by microscopy. In addition plates will be sent to GSK Biologicals in Rixensart for analysis on an automated ELISPOT-counter. All results will be expressed as number of of CS-reactive cells per million total Ig-positive cells.

APPENDIX G VACCINE SUPPLIES, PACKAGING AND ACCOUNTABILITY

It is NOT permitted to use any of the supplies provided by GSK Biologicals for purposes other than those specified in the protocol. Unused supplies will be collected by GSK Biologicals on completion of the study. Used vaccine vials/pre-filled syringes/containers can be disposed on site according to local biosafety standard for disposal of biological waste material.

1. Vaccine supplies

GSK Biologicals will supply the following amounts of numbered doses of study vaccines, sufficient to administer 3 doses to all subjects as described in the present protocol.

- Sufficient doses for 90 recipients of the candidate vaccine RTS,S/AS02D (doses of RTS,S vaccine in monodose vials and doses of AS02D adjuvant in pre-filled syringes).
- Sufficient doses for 90 recipients of the candidate vaccine RTS,S/AS01E (doses of RTS,S vaccine in monodose vials and doses of AS01E adjuvant in pre-filled syringes).

An additional 3% of their respective amounts of RTS,S/AS02D and RTS,S/AS01E will be supplied for replacement in case of breakage, bad storage conditions or any other reason that would make the vaccine unusable (i.e., given by mistake to another subject).

All pre-filled syringes and vials must be accounted for on the form provided.

2. Vaccine packaging

The vaccines will be packed in labeled boxes. The box label will contain, as a minimum, the following information: study number, abbreviated title, treatment number, lot number (or numbers, when double-blind), instructions for vaccine administration.

3. Vaccine accountability

The investigator or pharmacist must sign a statement that he/she has received the clinical supplies for the study. At all times the figures on supplied, used and remaining vaccine doses should match. At the end of the study, it must be possible to reconcile delivery records with those of used and unused stocks. An explanation must be given of any discrepancies.

After approval from GSK Biologicals, used vaccine vials/syringes should be destroyed at the study site using locally approved biosafety procedures and documentation unless otherwise described in the protocol. If no adequate biosafety procedures are available at the study site, the used vaccine vials/syringes are to be returned to an appropriate GSK Biologicals site for destruction in accordance with current GSK SOP WWD-1102. Unused vaccine vials/syringes will be disposed at the local GSK Biologicals site in accordance with GSK SOP WWD-1102. If no processes for destruction of unused

vaccines are in place in the local GSK Biologicals site; the unused vials/syringes must be returned to GSK Biologicals in Rixensart, Belgium.

4. Transfers of clinical vaccines or products from country medical department or dispatch center to study sites or between sites

Storage temperatures must be maintained during transport and deviations must be reported to Logistics and Packaging for guidance. All transfers of clinical vaccines or products must be documented using the Clinical Supply Transfer Form. If the duration of the transfer is less than four hours, a transportable fridge or any suitable container (e.g. Styrofoam container) with a maximum of eight refrigerated cold packs (cooling elements) must be used in order to maintain the vaccines at 2°to 8°C during transport. If the duration is more than four hours, a transportable fridge or any suitable container (e.g. Styrofoam container) with a minimum of eight cold packs (cooling elements) must be used as well as a temperature monitoring system that must be placed as close as possible to the doses and checked upon reception at the final destination. Never place frozen cold packs or dry ice inside vaccine/product boxes for vaccine that must be kept at +4°C in order to avoid cold-chain deviation (e.g. frozen vaccines). Exceptions to these instructions are detailed in product-specific transport guidelines.

5. Labels for sample identification

The investigator will receive labels from GSK Biologicals to identify samples taken from each subject at each time point. Each label will contain the following information: study number, treatment number, sampling time point (e.g., post vaccination 3), timing (e.g., study Month 7).

6. Other supplies provided by GSK Biologicals

In addition to the vaccines, the study documentation and the sample labels, the investigator will receive the following supplies:

- tubes with screw caps for serum samples,
- racks for the tubes of serum.

APPENDIX H ADMINISTRATIVE CHANGE TO THE PROTOCOL

GlaxoSmithKline Biologicals	
**Clinical Research & Development**	
**Protocol Administrative Change Approval**	
**eTrack study number**	105874
**eTrack abbreviated title**	Malaria-046
**Protocol title**	A Phase II randomized, double-blind bridging study of the safety and immunogenicity of GlaxoSmithKline Biologicals' candidate *Plasmodium falciparum* malaria vaccine RTS,S/AS01E (0.5 mL dose) to RTS,S/AS02D (0.5 mL dose) administered IM according to a 0, 1, 2-month vaccination schedule in children aged 18 months to 4 years living in Gabon
**Administrative change number**	Administrative Change 1
**Administrative change date**	16 February 2006
**Co-ordinating author**	Conor Cahill, Scientific Writer

Rationale/background for changes:

Previous studies of the RTS,S antigen have been completed under BB-IND 10514 and BB-IND 11220. In December 2006, GlaxoSmithKline Biologicals applied to the FDA for a new IND under which to carry out studies with the RTS,S/AS01 vaccine. Since completion of the final version of the study protocol, an IND number has been allocated; BB-IND 12937.

The Final Version of the protocol dated 07 December 2005 stated 'BB-IND to be determined' in all sections where the BB-IND number was to be quoted. For this Administrative Change that text has been deleted, and the new BB-IND number added.

In addition, the Local Safety Monitor for the study has changed from Jenny Dörnemann to Grégoire Adzoda. The relevant details have been changed throughout the protocol.

Amended text has been indicated in bold italics in the following sections:

Cover pages: ~~'BB-IND to be determined'~~ ***BB-IND 12937***

~~Jenny Dörnemann,~~ Grégoire Adzoda
Hôpital Albert Schweitzer,
B.P.~~ 118, Lambaréné,~~ ***13090, Libreville***
Gabon.

Tel: ~~+241.077.79.225~~ ***+00 241 07 35 83 33.***
Fax: +241.581.196
~~email: jennydoernemann@gmx.de~~

Investigator Approval page: ~~'BB-IND to be determined'~~ ***BB-IND 12937***

Section 8.7.2:

~~Jenny Dörnemann,~~ Grégoire Adzoda
Hôpital Albert Schweitzer,
B.P.~~ 118, Lambaréné,~~ ***13901, Libreville***
Gabon.
Tel: ~~+241.077.79.225~~ ***+00 241 07 35 83 33.***
Fax: +241.581.196
~~email: jennydoernemann@gmx.de~~ ***gadzoda@yahoo.fr***

Appendix E:

~~Jenny Dörnemann,~~ Grégoire Adzoda
Hôpital Albert Schweitzer,
B.P.~~ 118, Lambaréné,~~ ***13901, Libreville***
Gabon.
Tel: ~~+241.077.79.225~~ ***+00 241 07 35 83 33.***
Fax: +241.581.196
~~email: jennydoernemann@gmx.de~~ ***gadzoda@yahoo.fr***

GlaxoSmithKline Biologicals
Clinical Research & Development
Protocol Administrative Change Approval

**eTrack study number**	105874
**eTrack abbreviated title**	Malaria-046
**Protocol title**	A Phase II randomized, double-blind bridging study of the safety and immunogenicity of GlaxoSmithKline Biologicals' candidate *Plasmodium falciparum* malaria vaccine RTS,S/AS01E (0.5 mL dose) to RTS,S/AS02D (0.5 mL dose) administered IM according to a 0, 1, 2-month vaccination schedule in children aged 18 months to 4 years living in Gabon
**Administrative change number**	Administrative Change 1
**Administrative change date**	16 February 2006
**Approved by:**	
	Opokua Ofori Anyinam<br>Clinical Development Manager — dd-mm-yyyy

GlaxoSmithKline Biologicals
Clinical Research & Development
Protocol Administrative Change Approval

**eTrack study number**	105874
**eTrack abbreviated title**	Malaria-046
**Protocol title**	A Phase II randomized, double-blind bridging study of the safety and immunogenicity of GlaxoSmithKline Biologicals' candidate *Plasmodium falciparum* malaria vaccine RTS,S/AS01E (0.5 mL dose) to RTS,S/AS02D (0.5 mL dose) administered IM according to a 0, 1, 2-month vaccination schedule in children aged 18 months to 4 years living in Gabon
**Administrative change number**	Administrative Change 1
**Administrative change date**	16 February 2006

Agreed by:

Investigator: Bertrand Lell

Investigator signature:

____

Date: dd-mm-yyyy

____
